# Genomic dissection of hoof and leg conformation in Swiss dairy cattle populations reveals polygenic architecture and a recessive *HYAL1* nonsense variant affecting longevity in Holstein cattle

**DOI:** 10.1186/s12711-026-01068-3

**Published:** 2026-07-05

**Authors:** Stefan Vasiljevic, Franz R. Seefried, Sarah Widmer, Naveen K. Kadri, Alexander S. Leonard, Qiongyu He, Hubert Pausch, Florian Besnard, Aurélien Capitan, Cord Drögemüller, Joana Jacinto

**Affiliations:** 1 Institute of Genetics, Vetsuisse Faculty, University of Bern, 3012 Bern, Switzerland; 2 Qualitas AG, 6300 Zug, Switzerland; 3 Institute of Agricultural Sciences, Animal Genomics, ETH Zürich, 8092 Zurich, Switzerland; 4 Université Paris-Saclay, INRAE, AgroParisTech, GABI, 78350 Jouy-en-Josas, France; 5 Clinic for Ruminants, Vetsuisse Faculty, University of Bern, 3012 Bern, Switzerland

## Abstract

**Background:**

The genetic architecture of bovine hoof and leg conformation traits remains incompletely understood, particularly in dairy cattle and with respect to non-additive effects. Moreover, the limited genetic resolution of previous studies hampered the identification of candidate variants. This study aimed to investigate traits in Swiss Holstein (HO) and Brown Swiss (BS), comprising bone structure, heel depth, foot angle, locomotion, rear leg rear view, and rear leg set in HO, and heel depth, foot angle, hock quality, and rear leg side view in BS, by (1) estimating trait heritabilities, (2) identifying QTL using additive and non-additive GWAS based on imputed whole-genome sequence (WGS) variants, (3) detecting candidate genes and variants using Bayesian fine-mapping, (4) assessing the effects of the recessive *HYAL1* on conformation, longevity, and production traits in French HO, and (5) clinically characterizing *HYAL1-*homozygous HO.

**Results:**

Heritability estimates ranged from 0.10 to 0.28 in HO and from 0.10 to 0.26 in BS. Additive and non-additive GWAS detected 25 significant associated regions (*P* ≤ 5 × 10^−8^) for bone structure and locomotion in HO and for heel depth and hock quality in BS. Conditional analyses reduced these to 15 unique QTL and Bayesian fine-mapping prioritised 17 candidate variants (3 in HO, 14 in BS), including 15 additive, one dominant, and one recessive effect. A nonsense variant in *HYAL1* (NP_001017941.1:p.(Gln93*)) showed a significant recessive effect on bone structure in HO (*P* = 1.62 × 10^–17^). The harmful allele was observed predominantly in HO at 4.4% in Swiss and 8.4% in French HO. Analyses in the French population further showed adverse effects on six-year survival, indicating early culling after the first lactation, and 14 routinely recorded conformation and production traits, most notably locomotion and rear leg rear view. Clinical examination of *HYAL1*-homozygotes revealed polysynovitis.

**Conclusions:**

This study indicates a predominantly polygenic architecture of hoof and leg conformation traits in HO and BS cattle and show that integrating imputed WGS data with non-additive analyses of proxy phenotypes improves the detection and refinement of QTL and candidate variants. In particular, the identification of a recessive *HYAL1* nonsense variant associated with bone structure in HO uncovered a hidden Mendelian disorder.

**Supplementary Information:**

The online version contains supplementary material available at 10.1186/s12711-026-01068-3.

## Background

Historically, the main goal of traditional dairy cattle breeding has been to maximize profitability, with a strong focus on improving production traits [1]. For example, intense selection for milk yield has nearly doubled the average per-cow production over the past forty years [2]. However, this production-oriented selection has often brought unintended negative consequences for functional traits, thereby limiting overall genetic progress [3, 4]. In fact, a cow’s lifetime profitability depends not only on milk yield, but also on her health, fertility, feed efficiency, welfare and longevity [5, 6].

Body conformation traits have been recognized as valuable indicators of functional traits [7, 8]. Hoof and leg conformation traits are of particular importance as they are associated with hoof health [9] and locomotion [10–12]. Furthermore, poor hoof health and impaired locomotion are both closely linked to lameness in dairy cows [13], which is one of the most prevalent health and welfare issues affecting dairy cattle [14, 15]. Therefore, hoof and leg conformation traits can serve as useful indicators of susceptibility to lameness [16]. Direct genetic improvement of hoof disorders remains difficult due to incomplete phenotypic recording at population scale and strong environmental influences [17–19]. Consequently, indirect selection based on hoof and leg conformation traits as proxy phenotypes has become a practical alternative, as these traits are routinely recorded in several dairy cattle breeding programs using standardized linear scoring systems [20], show moderate heritability (ℎ^2^) from 0.05 to 0.37 [10, 12, 16, 21–23], and have a positive genetic correlation with hoof health [10, 24].

Several genome-wide association studies (GWAS) have identified quantitative trait loci (QTL) for hoof and leg conformation traits in Holstein (HO) cattle [6, 16, 22, 23, 25, 26]. However, the interpretation of these associations remains challenging, as association signals are often heterogeneous and causal variants cannot be clearly resolved due to linkage disequilibrium (LD) [27]. In Brown Swiss (BS), previously reported associations were based on composite leg conformation scores rather than linear evaluations of individual hoof and leg traits [28]. Consequently, the genetic architecture of these traits remains incompletely characterized in dairy cattle [6, 16, 26, 28]. To date, no high-resolution GWAS has specifically targeted hoof and leg conformation traits in Swiss dairy cattle populations [28].

GWAS for quantitative traits primarily rely on the additive genetic model [27], which assumes that the phenotypic effect increases linearly with each copy of the tested allele. While this model is statistically powerful, captures a major portion of the additive heritability [29], and is well suited for detecting polygenic effects, it may be unable to identify loci that act under non-additive inheritance [30]. To overcome this limitation, dominant and recessive non-additive models can be applied as complementary approaches. In a dominant model, the presence of at least one copy of the tested allele is sufficient to alter the phenotype [31], resulting in similar phenotypic values for heterozygous and homozygous carriers of the tested allele [32]. In contrast, the recessive model evaluates whether the phenotypic effect manifests only in individuals homozygous for the tested allele, a pattern often observed for rare and deleterious variants causing Mendelian disorders [30, 33, 34]. Although these non-additive models can detect loci missed by additive GWAS, they often have lower power, particularly when rare genotype classes are involved. A key limitation of GWAS is that, even with the high resolution provided by imputed whole-genome sequence (WGS) variants, associated signals often span broad genomic intervals due to extended linkage disequilibrium [35, 36]. This restricts the ability to identify causal variants without further analysis. To refine these regions, fine-mapping approaches are required [37]. Classically, stepwise conditional analysis has been used to identify independent association signals by re-evaluating loci after including the lead single nucleotide variant (SNV) as a covariate [38]. However, this approach does not quantify the probability of any specific, highly linked variant being causal. To address this, Bayesian fine-mapping methods have been developed to estimate the posterior probability of causality for each variant. This enables the construction of credible sets containing candidate causal variants [39–43].

Previous GWAS have identified genomic regions associated with hoof and leg conformation traits in dairy cattle, but analyses based on medium-density marker panels and purely additive models may provide limited resolution [44] and overlooked non-additive genetic effects [36]. In contrast, combining imputed-WGS variants with complementary inheritance models and Bayesian fine-mapping approaches could improve the prioritization of candidate loci, variants, and genes underlying these traits, although this approach has not yet been systematically investigated.

The aims of this study were to (1) estimate the additive ℎ^2^ of hoof and leg conformation traits in Swiss populations of HO and BS cattle, (2) identify associated genomic regions using additive and non-additive GWAS based on imputed WGS data, (3) detect functional candidate genes and prioritize candidate variants in associated genome regions, (4) assess the effects of a recessive *HYAL1* nonsense variant on conformation, longevity, and production traits in French HO, and to (5) clinically characterize *HYAL1* homozygous animals in the Swiss HO population.

## Methods

### Genomic data

The genotypic datasets consisted of two distinct Swiss dairy cattle populations. Population 1 (113,510 animals) encompassed HO, Simmental (SI) and Swiss Fleckvieh (SF), while population 2 (74,832 animals) included BS and Original Braunvieh (OB). Animals were genotyped using ten versions of high throughput genotyping arrays encompassing between 20,000 and 777,000 markers. SNV positions were determined according to the ARS-UCD1.2 bovine reference genome (BosTau9) [45, 46]. Initial quality control (QC) of the array-typed data was performed using PLINK v1.9 [46, 47] separately for each breed-array combination; samples and autosomal SNVs with > 10% missing genotypes were excluded. Following initial QC, genotypes were imputed to the WGS level in a two-step procedure using Beagle v5.4 [48]. The imputation was performed within the two populations. First, array genotypes were imputed to high-density (HD) level (Illumina BovineHD BeadChip) using reference panels of 1,138 animals in population 1 and 1,183 in population 2. Subsequently, sequence variants were imputed using imputed HD genotypes: for HO, SF and SI, 37,153,418 variants were imputed using 1,464 reference animals from the 1000 Bull Genomes Project, Run 9 [49], while for BS and OB, 26,265,006 variants were imputed using a reference panel of 607 in-house sequenced animals [50]. Hardy–Weinberg equilibrium (HWE) and minor allele frequency (MAF) were calculated using PLINK v1.9 [47]. Ultimately, based on a pedigree-based breed assignment threshold of ≥ 87.5%, a core set of 87,806 HO and 52,202 BS animals were retained for this study, with final datasets containing 36,256,390 SNVs for HO and 24,068,946 SNVs for BS. From these final datasets, two specific subsets were extracted: a low-density subset of 6,748 SNVs for principal component (PC) analysis, comprising markers routinely available across genotyping platforms and selected for robust SNV-based relatedness checks, which were sufficient to capture the main population structure, and an HD subset of 777,962 SNVs corresponding to the Illumina BovineHD BeadChip. The HD subset was defined by positional matching of imputed sequence variants to BovineHD array markers, resulting in 653,835 SNVs in HO and 633,885 SNVs in BS, which were used for GWAS and ℎ^2^ estimation.

### Phenotypic data

Phenotypic data for hoof and leg conformation traits were provided by the corresponding breeding associations, namely Swissherdbook, Holstein Switzerland, and Braunvieh Switzerland [51–53]. Traits were scored according to breed-specific linear descriptive classification schemes defined by the corresponding breeding organizations under ICAR guidelines [20, 54, 55]. Therefore, traits with the same name were not necessarily scored identically across breeds. For HO, the six traits analysed were bone structure (BST), heel depth (HDE), foot angle (FAN), locomotion (LOC), rear leg rear view (RLR), and rear leg set (RLS). For BS, the four analysed traits included HDE, FAN, hock quality (HQU) and rear leg side view (RLV). Except for HDE in HO, which was measured in millimetres, all traits were scored on a scale from 1 to 9 [20].

To prepare the phenotype data for analysis, a series of filtering steps was applied. First, only data recorded between September 2011 and August 2022 were considered since the number of cows with both phenotypes and genotypes was low from earlier periods. Second, the dataset was restricted to first lactation phenotype data since records from the later lactations may be biased due to on-farm selection decisions and are therefore not population representative. Records with missing values for the target phenotype were removed, and only the earliest available record for each animal within the first lactation was retained. To ensure adequate data for model fitting, only farms with at least five records were retained.

For subsequent phenotype correction breed-specific linear mixed-effects models were fitted to adjust phenotypes using the lme4 package v1.1–34 [56] in R v 4.3.1 [57]. Models were fitted with the lmer function using maximum likelihood estimation [56]. Model selection was performed by backward elimination based on deviance minimization [58]. This procedure was repeated for all traits, and the model most frequently selected across phenotypes was retained. For each HO trait, the selected model included as fixed effects the classifier-by-year, age at scoring grouped into months (< 24 months-old, from 25 to 40 months-old, and > 40 months-old), days in milk (grouped in a 60-day windows from day 5 to 305), and udder fullness. Udder fullness describes how much milk the udder contains at the time of assessment, and it was categorised as one of three levels ranging from empty to full. A similar correction model was applied to BS traits, except for udder fullness, which was replaced by the time of recording during the day grouped into five-time windows of 2-h intervals (08h00-10h00, 10h01-12h00, 12h01-14h00, 14h01-16h00, and 16h01-18h00). In both models, the farm was included as a random intercept. The residuals obtained from the fitted models were subsequently used as phenotypes for the GWAS and are referred to as corrected phenotypes.

Finally, the number of animals with both genotype and phenotype data was 21,535 for all HO traits, except for HDE, which included 21,530 animals, and 15,724 for all BS traits.

### Heritability estimation and GWAS

An additive genomic relationship matrix (GRM) was constructed using the HD panel and calculated using GCTA v1.94.1 [59]. PC analyses were performed in PLINK v1.9 using the low-density 6748 SNV subset [47].

Heritability was estimated in GCTA using the restricted maximum likelihood (REML) method [59]. The additive GRM was fitted as a random effect, with PC1 to PC4 included as fixed covariates. Computations were performed separately for each trait within breed. For comparison, routine heritability values from the national breeding value evaluations (ℎ^2^_EBV_) were also reported [52, 53].

GWAS were carried out using the mixed linear model association (MLMA) method implemented in GCTA v1.94.1 [59]. The MLMA model used for association testing is presented in Eq. (1):

1 $$ {\mathbf{y}}_{{{\mathbf{res}}}} = {\mathbf{{\rm X}\beta }} + {\mathbf{x}}_{{{\mathbf{snv}}}} \beta_{snv} + {\mathbf{g}} + {\mathbf{e}}, $$

where **y**_**res**_ denotes the vector of corrected phenotypes, defined as the residuals from the preliminary linear mixed model fitted with the lme4 package. The matrix **Χ** specifies the fixed covariates, comprising the first four PC, with **β** representing their corresponding vector of fixed effects coefficients. The variable **x**_**snv**_ is the vector of genotypes for the tested SNV, with its effect represented by *β*_*snv*_. Population structure and relatedness were accounted for through the vector of random polygenic effect **g**, assumed to follow the distribution shown in Eq. (2):

2 $$ {\mathbf{g}} \sim N\left( {0,{\mathbf{G}}_{g}^{2} } \right), $$

where **G** represents the additive GRM and *σ*_*g*_^*2*^ denotes the additive genetic variance. Finally, Residual effects were modeled by the vector **e**, assumed to follow the distribution shown in Eq. (3):

3 $$ {\mathbf{e}} \sim N\left( {0, {\mathbf{I}}_{e}^{2} } \right). $$

In Eq. (3), **I** denotes the *n* × *n* identity matrix (*n* representing the sample size) and *σ*_*e*_^*2*^ the residual variance. The statistical significance of each SNV was assessed using a Wald test implemented in GCTA.

An additive and two non-additive (recessive and dominant) genetic models were considered by varying the coding of **x**_**snv**_. In the additive model, genotypes were coded as 0 (homozygous for the reference allele, ref/ref), 1 (heterozygous, ref/var), and 2 (homozygous for the alternative allele, var/var). In the dominant model, genotypes were recoded as 0 (ref/ref) and 1 (ref/var and var/var), whereas in the recessive model they were recoded as 0 (ref/ref and ref/var) and 1 (var/var) [30]. In all models, the additive GRM was included as a random effect, and PC1 to PC4 were fitted as fixed covariates to correct for relatedness and population structure. Independent GWAS were conducted for each trait within breed. As an initial genome-wide screening step, the HD subset was analysed using a suggestive significance threshold of 1 × 10^−6^ to identify genomic regions for further analysis. This threshold was chosen as an approximate significance cutoff based on the number of largely independent SNVs in the HD subset after LD pruning with PLINK (window size = 5 SNVs, step size = 200 SNVs, r^2^ threshold = 0.3) [47], which yielded approximately 50,000 independent markers and corresponded to a Bonferroni-corrected threshold of 1 × 10^−6^. For these HD-subset GWAS, genomic inflation factors (λ) were calculated to assess inflation or deflation of test statistics.

### Fine-mapping, candidate gene selection and variant prioritisation

Chromosomes with suggestively associated (*P* ≤ 1 × 10^−6^) variants from the HD subset were re-analysed using a second MLMA that incorporated all available imputed SNVs. Within each chromosome, joint conditional analyses were performed with GCTA-COJO v1.94.1 [38] to identify independent association signals, using a genome-wide significance threshold of 5 × 10^−8^ and a LD window of 10 Mb. The threshold of 5 × 10^−8^ was used as a conventional genome-wide significance cutoff, corresponding to a 5% Bonferroni-corrected significance threshold for 1,000,000 independent tests [36]. The 10 Mb window was retained for the COJO analysis as the standard setting in GCTA, ensuring that a sufficiently broad LD region was considered when identifying independent lead SNVs [38, 59]. Lead SNVs were defined as the most significant SNVs within each independent signal that remained significant after conditioning on previously selected SNV.

For each lead SNV, a 2 Mb region (1 Mb upstream and 1 Mb downstream) was extracted for fine-mapping, as a pragmatic local interval to capture nearby linked variants [36, 60]. Regional association plots for these regions were generated in R using the locuszoomr package [61]. When additive and non-additive models produced overlapping windows, only the additive model was retained for interpretation, because it provides the most robust and best-powered representation of the association signal, capturing the largest share of genotype-related variation with stable effect estimates. SNVs within these regions were annotated with SnpEff v5.0 [62] on ARS-UCD1.2 (BosTau9) [45, 46]. Fine-mapping was conducted with PAINTOR v3.0 [42], a Bayesian fine-mapping tool, to estimate the posterior inclusion probability (PIP) of each SNV under the default parameter settings. Functional priors included binary indicators for the SnpEff impact categories LOW, MODERATE, and HIGH using k minus 1 coding with MODIFIER as the reference. The Functional and Evolutionary Trait Heritability (FAETH) score v1.1 [63] was calculated as the average of per-variant ℎ^2^ values across multiple genome partitions, thereby capturing information on gene regulation, evolutionary conservation, and trait ℎ^2^. In this study, the FAETH scores was min–max scaled, and subsequently incorporated into PAINTOR as functional priors, together with the categorical SnpEff impact annotations. FAETH scores were initially reported on the UMD3.1 bovine genome assembly [63]. To match them with our genotype data based on ARS-UCD1.2, variant coordinates were converted using UCSC liftOver, with the bosTau6ToBosTau9.over.chain.gz chain file obtained from the UCSC Genome Browser [64].

The term "candidate gene" was used to describe genes based on their known function and/or associated limb, foot, bone, connective tissue or nail-related phenotypes in mammalian species. A literature review was also conducted on the positional genes to evaluate their biological plausibility with the associated phenotype. Furthermore, positional genes overlapping or nearest to the filtered variants were then prioritised using VarElect v5.23 (LifeMap Sciences Inc.) [65] with the phenotype query “limb OR foot OR bone OR connective tissue OR nail”. The VarElect relevance score from this query served as a phenotype-based gene score.

The term "candidate variant" was used to describe a variant considered biologically plausible based on the function of the affected gene and/or its associated phenotype in mammalian species. Credible sets were defined by retaining SNVs with PIP ≥ 0.95. Credible variants were then filtered to retain only those in strong LD with the lead SNV (r^2^ ≥ 0.75), with dosage R^2^ ≥ 0.6, MAF ≥ 0.01, and -log_10_(P) ≥ 6. Dosage R^2^ was used as a measure of imputation accuracy, representing the squared correlation between imputed and true genotypes. This filtering step was intended to retain only well-imputed variants with strong statistical and LD support for the local association signal. Candidate variants were subsequently ranked according to the following criteria, in order: predicted impact severity (HIGH > MODERATE > LOW > MODIFIER), -log_10_(P), LD (r^2^), FAETH score, and PIP. Finally, candidate variants were selected from the highest-ranked positions after this filtering and prioritization procedure.

### Characterization and genotyping of the HYAL1 variant

The identified recessive candidate nonsense variant in *HYAL1* associated with BST in HO cattle was further characterized and genotyped for population-based and clinical follow-up analyses. The variant had been added earlier to this study to the custom part of a recent version of the Swiss routine Axiom HD genotyping array [66]. At the time of analysis, Axiom HD genotypes were available for 2604 animals from five main Swiss dairy breeds (BS, HO, OB, SF, SI) routinely genotyped within the framework of national genomic evaluations including 923 HO animals.

The *HYAL1* variant was also added earlier to the Illumina EuroGMD SNP array as part of several reverse genetic projects in France [34, 67]. It was included, with a specific probe designed as follows ACCAAACACAGGCTCCCCAGCAGATGTATAGTAAGGGTAGGTACCCAGCT[G/A]. At the time of analysis, EuroGMD SNP array genotypes were available for 2,252,399 animals from 21 French cattle breeds routinely genotyped within the framework of national genomic evaluations including 1,159,426 HO animals. This dataset was used to monitor the occurrence of the *HYAL1* variant across breeds and to evaluate its phenotypic effects in French HO cattle, following previously described approaches [68, 69].

The comprehensive variant catalogue with 5116 bovine genomes of more than 130 breeds from run 9 of the 1000 Bull Genomes Project was available to further investigate the allelic distribution of the identified candidate variant in *HYAL1* within a global control cohort [49].

PCR and Sanger sequencing were used to validate and genotype the *HYAL1* nonsense variant in seven var/var animals that underwent clinical examination. PCR products from genomic DNA, using the following primers: 5′-ACCAGCCCTTCACTACCATC-3′ (forward primer) and 5′- CTTCTGTACCAATGCCCGTG 3′ (reverse primer),were amplified using AmpliTaq Gold 360 Master Mix (Thermo Fisher Scientific, Waltham, MA, USA) and the PCR amplicons were directly sequenced on an ABI3730 capillary sequencer (Thermo Fisher Scientific, Darmstadt, Germany).

### Survival analysis and effect estimation for the HYAL1 genotypes

The effects of the three *HYAL1* genotypes (ref/ref, ref/var, and var/var) were estimated for 34 traits routinely recorded for selection in France, including production, morphological, health and fertility traits. The analyses were based on yield deviation data provided by GenEval [70] and used linear mixed animal models, including the fixed effects for *HYAL1* genotype and year of recording, with individual polygenic effects fitted as random. Models were implemented in blupf90 + [71], and statistical significance was assessed using t-tests with Benjamini–Hochberg correction [72]. Effects were expressed in genetic standard deviations (GSD) to facilitate comparison among traits.

In addition, the effect of the *HYAL1* nonsense variant on mortality and culling was investigated over a six-year period in 328,505 female cattle born at least six years before the analysis date, for which data on date of birth, date of death, and cause of death were available in the French National Bovine Database. The population included 271,211 ref/ref, 54,629 ref/var, and 2,665 var/var animals. For each *HYAL1* genotype, daily counts and proportions of animals that were alive, dead (natural causes or euthanasia), or slaughtered were computed. To investigate longevity, animals were classified as alive or removed, with removed animals defined as those that were either dead or slaughtered. *HYAL1* genotype differences in the distribution between alive and removed animals were tested using Fisher’s exact test with Monte Carlo P-value estimation in R v 4.3.1 [73], performed per year of life. P-values were adjusted for multiple testing using the Benjamini–Hochberg false discovery rate procedure [72].

### Clinical examination

Alive HO var/var animals for the *HYAL1* recessive candidate variant were identified with the help of Swiss breeding organizations. Seven of them (cases 1–7) were selected for an on-farm clinical examination. The animals examined were selected based on being alive and their owners’ consent to participate in the study. The cohort included three 7-month-old heifers (cases 1–3), one first-lactation cow aged 3 years (case 4), two second-lactation cows aged 3.5 years (cases 5 and 6), and one third-lactation cow aged 4 years (case 7). All animals underwent a standardized general clinical examination. This included assessment of general condition (body condition score, behaviour, and posture) and recording of vital parameters (heart rate, respiratory rate, rectal temperature, mucous membrane colour, and capillary refill time). A systematic, organ-based examination was then conducted, comprising external evaluation (hair coat, skin, lymph nodes, eyes, and ears), cardiovascular system (cardiac auscultation and jugular vein assessment), and respiratory system (lung auscultation and cough evaluation). The digestive system was assessed through rumen fill scoring, rumen motility and stratification, abdominal auscultation and percussion, and faecal evaluation. In addition, the urinary and reproductive systems (including macroscopic urine assessment and udder examination), nervous system (level of consciousness, cranial and spinal reflexes, and proprioception), and musculoskeletal system (joint evaluation, limb function, and skeletal assessment, locomotion) were examined. Additionally, ultrasonography of the hock and fetlock joints using a 7.5Mz linear probe was performed.

## Results

### Hoof and leg conformation traits are low to moderately heritable in Swiss dairy cattle

The descriptive statistics for raw and corrected phenotypes are reported in Table 1. The estimated additive ℎ^2^ for HO and BS varied from low to moderate. For HO, the ℎ^2^ estimates ranged from 0.10 (standard error, SE = 0.007) for LOC to 0.28 (SE = 0.01) for BST. For BS, ℎ^2^ ranged from 0.10 (SE = 0.009) for HDE to 0.26 (SE = 0.01) for HQU (Table 1). These estimates were also broadly consistent with the corresponding ℎ^2^ values from national genetic evaluations, which ranged from 0.09 to 0.27 across the analysed traits (Table 1).

**Table 1:** Descriptive statistics and estimated heritability of hoof and leg conformation traits in HO and BS

Breed	Trait	Raw phenotype				Corrected phenotype			ℎ^2^ (SE)	ℎ^2^_EBV_
		Mean (SD)	Min	Max	Ideal	Mean (SD)	Min	Max		
HO	BST	5.71 (1.26)	1	9	8	0.00 (1.14)	− 4.82	3.84	0.28 (0.01)	0.27
	HDE	35.58 (5.20)	10*	60*	45*	0.00 (4.76)	− 25.06	25.57	0.13 (0.008)	0.15
	FAN	5.46 (1.18)	1	9	7	0.00 (1.10)	− 4.98	25.57	0.14 (0.008)	0.12
	LOC	5.62 (1.26)	1	9	9	0.00 (1.14)	− 4.88	3.95	0.10 (0.007)	0.09
	RLR	5.73 (1.39)	1	9	9	0.00 (1.25)	− 5.05	3.93	0.21 (0.009)	0.19
	RLS	5.12 (1.12)	1	9	5	0.00 (1.06)	− 4.50	3.82	0.24 (0.009)	0.23
BS	HDE	5.40 (1.08)	1	9	8	0.00 (0.95)	− 4.18	3.97	0.10 (0.009)	0.10
	FAN	5.34 (1.20)	1	9	6	0.00 (1.10)	− 5.29	4.11	0.14 (0.01)	0.13
	HQU	5.40 (1.87)	1	9	9	0.00 (1.71)	− 5.31	5.57	0.26 (0.01)	0.24
	RLV	4.86 (1.12)	1	9	5	0.00 (1.04)	− 4.15	4.61	0.21 (0.01)	0.21

^*^measured in mm

*SD* standard deviation, *Min* minimum, *Max* maximum, *Ideal* ideal score or measurement according to the breed-specific trait definition, ℎ^2^ additive heritability estimated by GCTA, *SE* standard error, ℎ^2^_EBV_ routine heritability from the national breeding value evaluations, *BST* bone structure, *HDE* Heel depth, *FAN* foot angle, *LOC* locomotion, *RLR* rear leg rear view, *RLS* rear leg set, *HQU* hock quality, *RLV* rear leg side view, *HO* Holstein, *BS* Brown Swiss

### Identification of several breed specific association signals using different models

The MLMA of the additive and two different non-additive models using the HD SNV panel identified a total of 45 suggestive QTL (*P* ≤ 1 × 10^–6^) across hoof and leg conformation traits in HO and BS. The complete overview of all suggestive regions, including genomic range, top SNV positions, and corresponding P-values, is provided in Supplementary Table S1. Furthermore, λ were close to 1, indicating no substantial inflation of test statistics (Supplementary Fig. S28-S37).

**Fig. 1:**
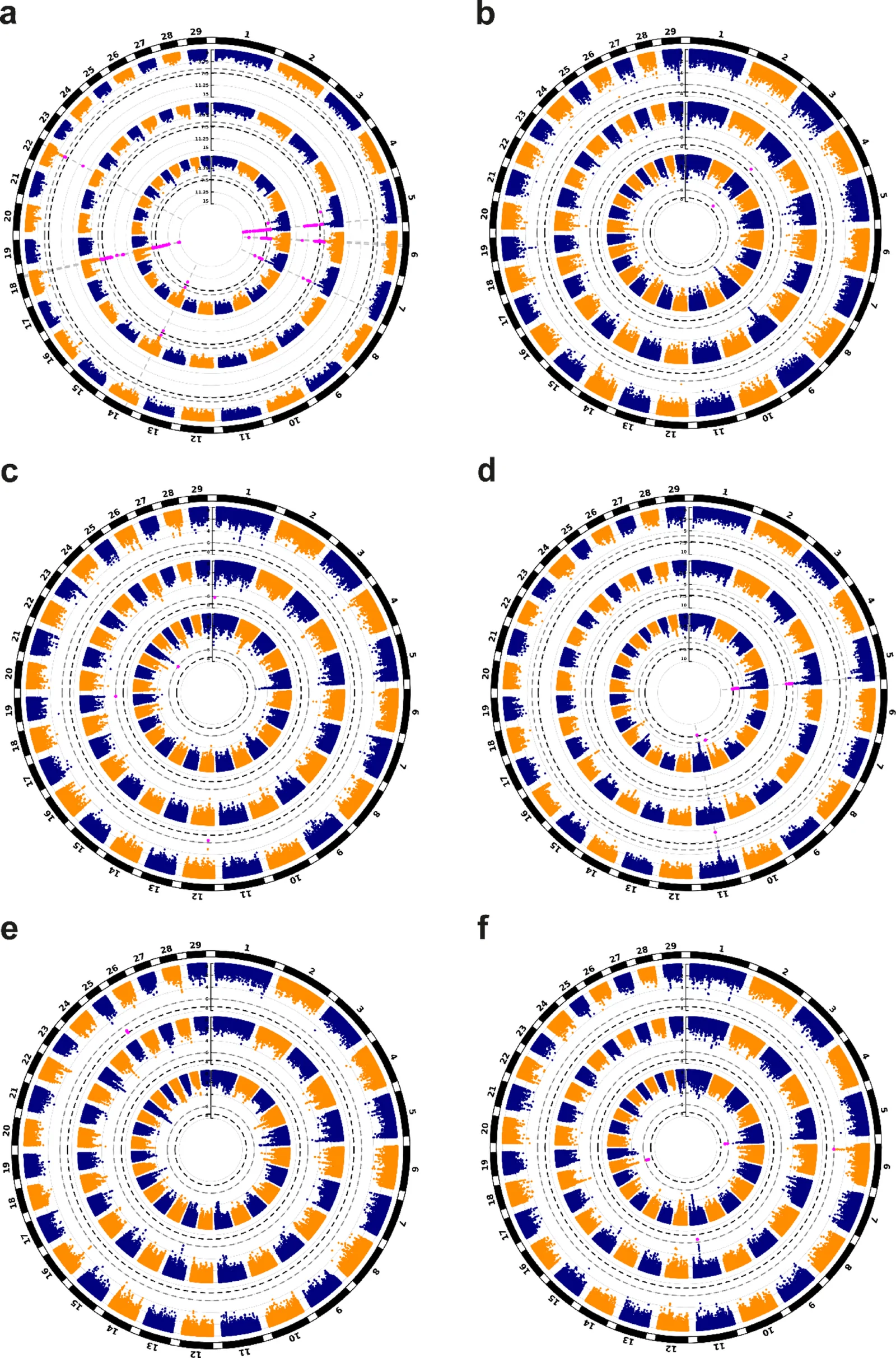
Overview of additive, dominant and recessive GWAS for six hoof and leg conformation traits in HO. Circular Manhattan plot showing the results of GWAS using the high-density SNV panel for **a** BST **b** HDE **c** FAN **d** LOC **e** RLR **f** RLS in HO, across the 29 autosomes, under three genetic models: recessive (outer ring), dominant (middle ring) and additive (inner ring). SNVs are plotted by genomic position, with the radial axis showing -log_10_(P). Suggestive (*P* ≤ 1 × 10^–6^; − log_10_(P) = 6.0) and genome-wide (*P* ≤ 5 × 10^–8^; − log_10_(*P*) = 7.3) significance thresholds were applied. SNVs exceeding the suggestive threshold are highlighted in magenta

In HO, a total of 27 suggestive QTL were detected across BST, HDE, FAN, LOC, RLR, and RLS (Fig. 1a–f). Of these, 12 were identified using the additive model located on chromosomes (chr) 3, 5, 6, 7, 10, 11, 14, 18 and 23; five were identified using the recessive model on chr 6, 11, 12, 22 and 25; and 10 were found using the dominant model on chr 1, 3, 5, 6, 7, 11, 14, 18 and 19. Overlapping suggestive QTL were observed for BST on chr 5, 6, 7, 14 and 18 between the additive and dominant models (Fig. 1a), and for LOC on chr 5 between the additive and dominant models and on chr 11 between the additive and recessive models (Fig. 1d).

**Fig. 2:**
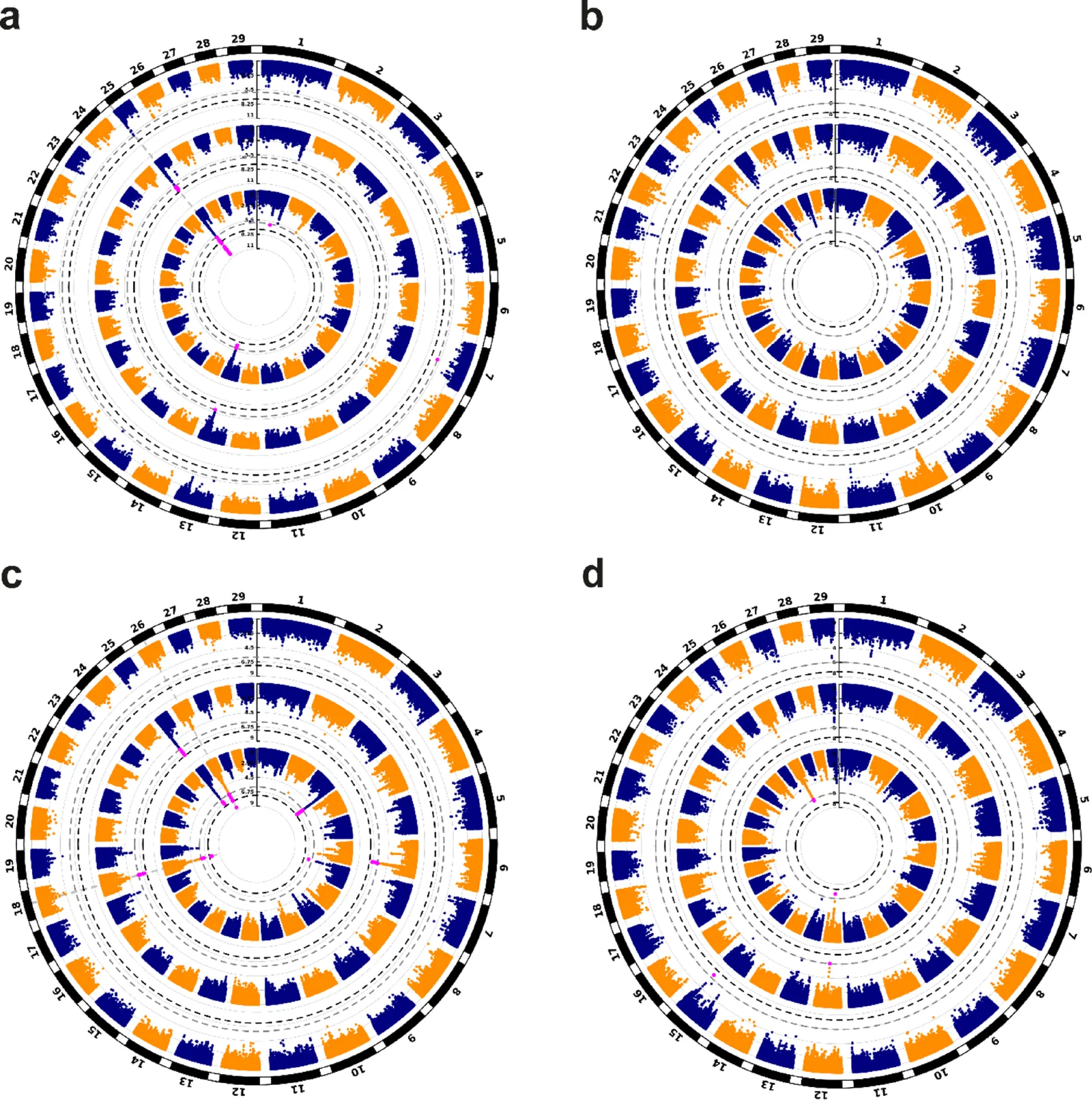
Overview of additive, dominant and recessive GWAS for four hoof and leg conformation traits in BS. Circular Manhattan plot showing the results of GWAS using the high-density SNV for **a** HDE **b** FAN **c** HQU **d** RLV in BS, across the 29 autosomes, under three genetic models: recessive (outer ring), dominant (middle ring) and additive (inner ring). SNVs are plotted by genomic position, with the radial axis showing –log_10_(P). Suggestive (*P* ≤ 1 × 10^–6^; − log10(P) = 6.0) and genome-wide (*P* ≤ 5 × 10^–8^; − log10(P) = 7.3) significance thresholds were applied. SNVs exceeding the suggestive threshold are highlighted in magenta

For BS, 18 suggestive QTL were identified across HDE, HQU, and RLV (Fig. 2a, c and d). These included 10 regions under the additive model on chr 1, 3, 7, 12, 13, 18, 25 and 26; two regions under the recessive model on chr 7 and 15; and six regions under the dominant model on chr 6, 12, 13, 18 and 25. Overlapping of the suggestive QTL between the additive and dominant models were observed for HDE on chr 13 and 25 (Fig. 2a), for HQU on chr 18 and 25 (Fig. 2c), and for RLV on chr 12 (Fig. 2d).

To refine the 45 suggestive QTL initially identified with the HD SNV panel, a second MLMA was conducted using all imputed SNVs on the corresponding chromosomes. This follow-up analysis identified a total of 25 significant QTL (*P* ≤ 5 × 10^–8^) (Table S2).

Fifteen significant genomic regions were identified for two different traits in HO: 11 were associated with BST and four with LOC. For BST, regions on chr 5, 6, 7, 14, 18, and 22 were significant under both the additive and non-additive models. However, the region on chr 22 was only significant under the recessive model. For LOC, the additive model identified regions on chr 5 and 11. The region on chr 5 was significant under the dominant model, and the region on chr 11 was significant under the recessive model. No overlapping regions were observed between BST and LOC (Table 2).

**Table 2:** Significant association signals and positional candidate genes for hoof and leg conformation traits in HO and BS

Breed	Trait	Model	Chr	Lead SNV				
				N independent signal(s)	Position (bp)	*P*-value	QTL interval	Candidate gene
HO	BST	Additive	5	1	105,547,292	3.93 × 10^–16^	104,547,532–106,547,170	*NTF3, KCNA1, NDUFA9, FGF6, FGF23, CCND2*
HO	BST	Additive	6	2	37,048,588	7.07 × 10^–24^	36,048,590–38,048,179	*PIGY, PKD2, SPP1, MEPE, IBSP*
					37,298,520	1.82 × 10^–8^	36,298,633–38,298,421	*PKD2, SPP1, MEPE, IBSP*
HO	BST	Additive	7	1	90,811,882	5.52 × 10^–11^	89,812,839–91,811,827	*ADGRV1*
HO	BST	Additive	14	1	24,325,400	3.21 × 10^–9^	23,325,792–25,325,229	*PLAG1, BPNT2*
HO	BST	Additive	18	1	57,574,852	2.11 × 10^–22^	56,574,893–58,574,848	*POLD1, ETFB, MIR99B, MIR125A, PPP2R1A*
HO	BST	Dominant	5	1	105,547,292	3.83 × 10^–14^	104,547,532–106,547,170	*NTF3, KCNA1, NDUFA9, FGF6, FGF23, CCND2*
HO	BST	Dominant	6	2	37,048,588	7.07 × 10^–24^	36,048,590–38,048,179	*PIGY, PKD2, SPP1, MEPE, IBSP*
					37,298,520	1.82 × 10^–8^	36,298,633–38,298,421	*PKD2, SPP1, MEPE, IBSP*
HO	BST	Dominant	7	1	90,811,882	4.57 × 10^–11^	89,812,839–91,811,827	*ADGRV1*
HO	BST	Dominant	14	1	24,325,400	3.21 × 10^–9^	23,325,792–25,325,229	*PLAG1, BPNT2*
HO	BST	Dominant	18	1	57,574,852	3.11 × 10^–20^	56,574,893–58,574,848	*POLD1, ETFB, MIR99B, MIR125A, PPP2R1A*
HO	BST	Recessive	22	1	50,135,713	3.44 × 10^–18^	49,135,960–51,134,158	*HYAL1, TRAIP, GMPPB, DAG1, AMT, RHOA, QARS1, MIR191, NDUFAF3, SLC25A20*
HO	LOC	Additive	5	1	108,760,988	5.42 × 10^–9^	107,761,169–109,760,788	*CACNA1C, IL17RA, ADA2, ATP6V1E1, PEX26, TUBA8, SOX10*
HO	LOC	Additive	11	1	44,806,402	1.22 × 10^–15^	43,806,510–45,806,350	*EDAR, RANBP2, SLC5A7, GCC2*
HO	LOC	Dominant	5	1	109,321,159	4.89 × 10^–8^	108,321,648–110,321,054	*CACNA1C, IL17RA, ADA2, ATP6V1E1, PEX26, TUBA8, SOX10, PLA2G6*
HO	LOC	Recessive	11	1	43,892,230	5.23 × 10^–10^	42,892,261–44,892,159	*EDAR, RANBP2, GCC2*
BS	HDE	Additive	13	1	61,329,100	5.94 × 10^–9^	60,329,758–62,328,857	*CSNK2A1, RBCK1, ID1, BCL2L1, POFUT1, ASXL1, DNMT3B, TPX2*
BS	HDE	Additive	25	1	1,483,751	5.30 × 10^–11^	484,002–2,483,588	*PIGQ, STUB1, GNPTG, CLCN7, TELO2, IFT140, MRPS34, IGFALS, GFER, TSC2, PKD1, ABCA3, CCNF, TBC1D24, KREMEN2, MAPK8IP3, TRAF7*
BS	HDE	Dominant	13	1	61,329,852	1.97 × 10^–8^	60,330,202–62,329,546	*CSNK2A1, RBCK1, ID1, BCL2L1, POFUT1, ASXL1, DNMT3B, TPX2*
BS	HDE	Dominant	25	1	1,815,922	6.94 × 10^–9^	816,482–2,815,881	*GNPTG, CLCN7, TELO2, IFT140, MRPS34, IGFALS, GFER, TSC2, PKD1, ABCA3, CCNF, TBC1D24, KREMEN2, MEFV, MAPK8IP3, TRAF7*
BS	HQU	Additive	3	1	118,733,137	1.45 × 10^–8^	117,733,467–119,733,028	*TWIST2, HDAC4, NDUFA10*
BS	HQU	Additive	18	1	35,316,184	2.09 × 10^–8^	34,316,206–36,314,919	*CBFB, MIR328, CTCF, ACD, LCAT, PRMT7, CDH3, CDH1, RIPOR1*
BS	HQU	Additive	25	1	1,226,363	2.35 × 10^–8^	226,786–2,226,229	*AXIN1, PIGQ, STUB1, GNPTG, CLCN7, TELO2, IFT140, MRPS34, IGFALS, GFER, TSC2, PKD1, ABCA3, CCNF, TBC1D24*
BS	HQU	Additive	26	1	23,248,278	3.23 × 10^–9^	22,249,367–24,248,244	*BTRC, POLL, DPCD, FBXW4, FGF8, GBF1, NFKB2, MIR146B, SUFU, TRIM8, CYP17A1, CNNM2, NT5C2*
BS	HQU	Dominant	6	1	88,208,422	5.42 × 10^–11^	87,208,448–89,208,308	*ADAMTS3, ALB, CXCL8, PPBP, PF4, ANKRD17*
BS	HQU	Dominant	18	1	35,316,184	5.08 × 10^–9^	34,316,206–36,314,919	*CBFB, MIR328, CTCF, ACD, LCAT, PRMT7, CDH3, CDH1, RIPOR1*

*Chr* chromosome, *P-value* nominal significance level of the association, *BST* bone structure, *HDE* Heel depth, *LOC* locomotion, *HQU* hock quality, *HO* Holstein, *BS* Brown Swiss

Ten significant genomic regions were detected for two different traits in BS: HQU and HDE. Analysis of HQU revealed six significant regions: four under the additive model on chr 3, 18, 25, and 26, and two under the dominant model on chr 6 and 18. Four significant regions were identified for HDE on chr 13 and 25, with two regions detected under the additive model and two under the dominant model (Table S2). An overlapping significant region between HQU and HDE was observed on chr 25 (Table 2).

Only the most significant regions were retained for further investigation. We performed a joint analysis of these regions using GCTA-COJO to identify independent signals and lead SNVs. In most instances, one lead SNV was identified per genomic region. The only exception was the BST region on chr 6, where two independent signals were detected under both the additive and dominant models. These distinct lead SNVs were located at positions 37,048,588 bp and 37,298,520 bp. A QTL window of ± 1 Mb was then defined around each lead SNV. Where additive and non-additive QTL windows overlapped, the additive model was prioritized, resulting in 15 QTL regions in HO and BS in total. All QTL and lead SNVs are reported in Table 2. At least one candidate gene was identified for all QTL (Table 2).

### Sequence resolution fine-mapping identifies several candidate causal variants for hoof and leg conformation traits in HO and BS

Of the identified 15 QTL, 10 contained at least one candidate variant, giving a total of 17 variants: 15 detected under the additive model, one under the recessive model, and one under the dominant model. In terms of functional annotation, the candidate variants included seven intronic, three upstream gene, two missense, two synonymous, one stop-gained, one frameshift, and one downstream gene variant. Full details of all variants included in each QTL region are provided in Supplementary Table S3. Specifically, three variants were identified in HO (one for BST and two for LOC), and 14 were found in BS (nine for HQU and five for HDE).

**Fig. 3:**
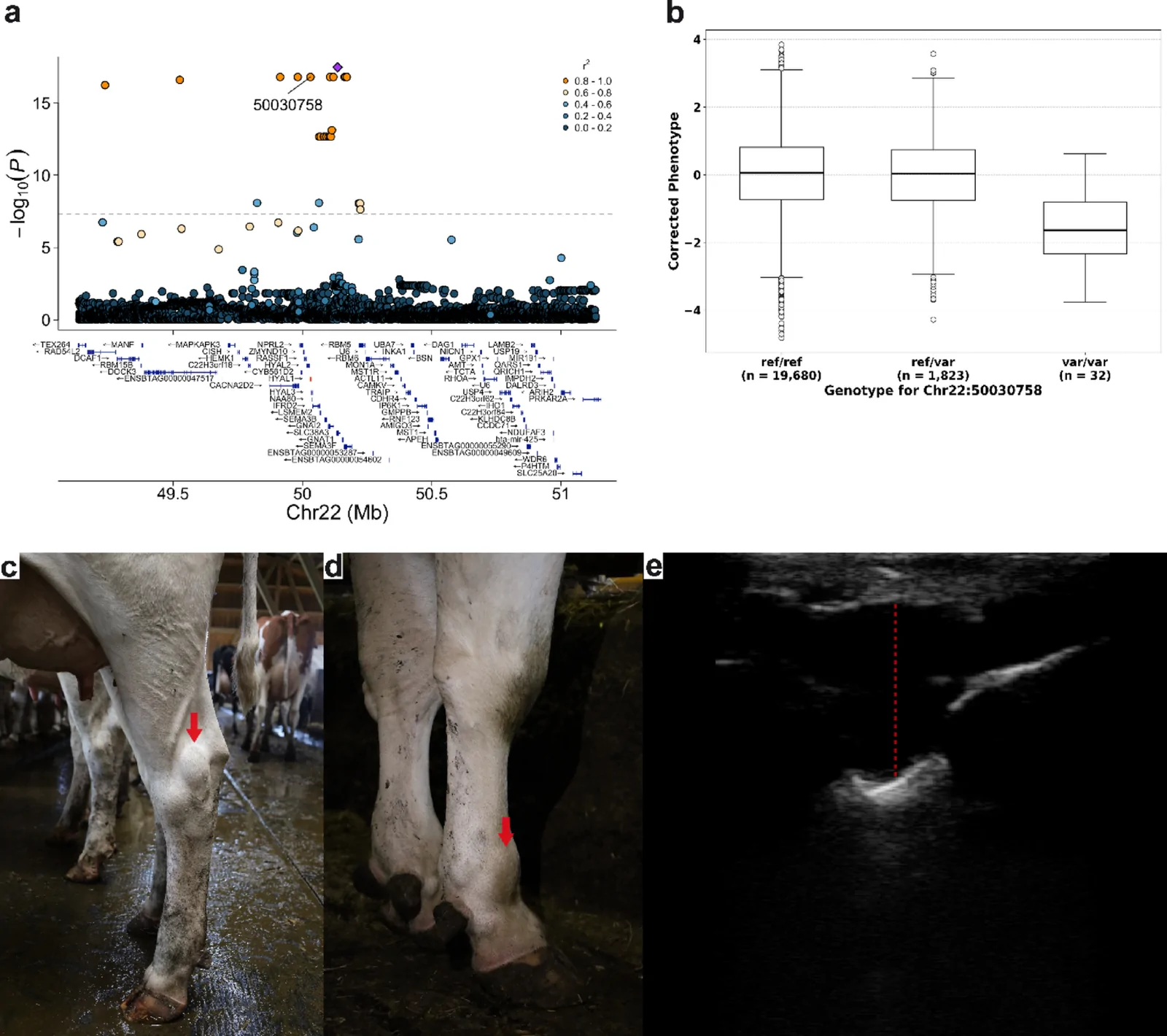
Features of the recessive QTL for bone structure on chromosome 22 in HO. **a** Regional association plot for BST on chromosome 22 in HO under the recessive model within a ± 1 Mb window around the lead SNV. Dot colours indicate linkage disequilibrium (r^2^) relative to the lead SNV. The candidate variant in *HYAL1* (chr22:g.50,030,758C > T) is labeled, and the lead SNV is shown as a violet diamond. The candidate gene *HYAL1* is indicated in red. **b** Boxplot showing the distribution of corrected BST phenotypes by genotype class for the candidate variant chr22:50,030,758 bp. **c** Rear leg lateral view of a HO cow carrying the deleterious homozygous alternative allele (case 4) showing enlargement of the fedlock joint (arrow). **d** Rear leg lateral view of a HO cow carrying the deleterious homozygous alternative allele (case 7) showing enlargement of the metacarpophalangeal joint. **e** Longitudinal sonogram (7.5-MHz linear transducer) of the dorsal aspect of the left tarsocrural joint showing the dorsal joint pouch with abundant serous content with a distended joint recess with a mainly anechoic effusion (dotted line = 7 cm) in a HO cow carrying the deleterious homozygous alternative allele (case 4)

Regarding BST in HO, a single candidate variant was prioritized under the recessive model on chr 22 at 50,030,758 bp, corresponding to a *HYAL1* nonsense variant (NP_001017941.1:p.(Gln93*)) (Fig. 3a,b). This variant showed the strongest allelic substitution effect among all candidate variants, with a highly significant (*P* = 1.62 × 10^–17^) estimated effect of -1.594 ± 0.187 phenotypic standard deviations of the corrected phenotype (Fig. 3c; Table 3).

**Table 3:** Characterization of candidate variants after QTL fine-mapping for hoof and leg conformation traits in HO and BS

Breed	Trait	Model	Chr	Position (bp)	MAF	r^2^	Effect size	SE	*P*-value	Dosage R^2^	Gene	Variant type	Feature ID: HGVS.c	HGVS.p
HO	BST	Recessive	22	50,030,758	0.043	0.991	− 1.594	0.187	1.62 × 10^–17^	0.93	*HYAL1*	Stop gained	NM_001017941.1: c.277C > T	NP_001017941.1:p.(Gln93*)
HO	LOC	Additive	5	108,760,988	0.31	1	0.089	0.015	5.42 × 10^–9^	0.95	*CACNA1C*	Intronic	XM_024992764.2: c.4143 + 1833 T > A	NA
HO	LOC	Additive	11	44,828,627	0.343	0.944	0.096	0.012	7.31 × 10^–15^	1	*GCC2*	Missense	NM_001192259.1: c.2612 T > G	NP_001179188.1:p.(Phe871Cys)
BS	HDE	Additive	13	61,320,050	0.117	0.995	− 0.115	0.020	1.99 × 10^–8^	0.98	*TPX2*	Synonymous	XM_005214599.5: c.675A > G	XM_005214599.5:p.(Glu225Glu)
BS	HDE	Additive	25	1,171,241	0.453	0.951	− 0.091	0.015	1.55 × 10^–9^	1	*TELO2*	Upstream	XM_010819183.4: c.-2974A > C	NA
BS	HDE	Additive	25	1,340,769	0.45	0.908	− 0.097	0.015	1.55 × 10^–10^	1	*MAPK8IP3*	Intronic	XM_010819109.3: c.1569-41C > G	NA
BS	HDE	Additive	25	1,690,471	0.464	0.959	− 0.098	0.015	1.15 × 10^–10^	1	*TRAF7*	Upstream	XM_024984795.2: c.-3491 T > G	NA
BS	HDE	Additive	25	1,836,217	0.466	0.945	− 0.096	0.015	2.04 × 10^–10^	1	*ABCA3*	Upstream	XM_010819068.4: c.-1003 T > G	NA
BS	HQU	Additive	3	118,271,945	0.348	0.919	− 0.171	0.033	3.12 × 10^–7^	1	*HDAC4*	Intronic	XM_024990281.2: c.1633-1227G > A	NA
BS	HQU	Additive	3	118,888,292	0.348	0.975	− 0.181	0.033	5.38 × 10^–8^	1	*NDUFA10*	Intronic	NM_176655.2: c.964-2960 T > C	NA
BS	HQU	Additive	18	35,124,569	0.456	0.794	0.175	0.032	6.7 × 10^–8^	1	*RIPOR1*	Frameshift	XM_024978978.2: c.539delC	XM_024978978.2:p.(Pro180fs)
BS	HQU	Additive	25	1,148,147	0.295	0.978	0.174	0.033	9.85 × 10^–8^	1	*CLCN7*	Synonymous	NM_001025331.1: c.465G > A	NM_001025331.1:p.(Leu155Leu)
BS	HQU	Additive	26	22,262,227	0.272	0.845	− 0.191	0.035	5.76 × 10^–8^	1	*BTRC*	Intronic	XM_005225560.5: c.433-15074A > C	NA
BS	HQU	Additive	26	22,433,281	0.273	0.851	− 0.194	0.035	3.81 × 10^–8^	0.98	*FBXW4*	Intronic	XM_059881836.1: c.782 + 6628 T > G	NA
BS	HQU	Additive	26	22,503,920	0.273	0.852	− 0.2	0.035	1.44 × 10^–8^	1	*FGF8*	Missense	NM_001206678.1: c.718G > A	NM_001206678.1:p.(Ala240Thr)
BS	HQU	Additive	26	23,248,278	0.303	1	− 0.2	0.034	3.24 × 10^–9^	1	*SUFU*	Downstream	NM_001098083.2: c.*1157G > A	NA
BS	HQU	Dominant	6	88,208,422	0.154	1	− 0.297	0.045	5.42 × 10^–11^	0.84	*ANKRD17*	Intronic	XM_024993124.2: c.7021 + 1564_7021 + 1566delTTT	NA

*Chr* chromosome, *MAF* minor allele frequency, r^2^ Squared correlation coefficient of linkage disequilibrium (LD) between the SNV and the lead variant, *Effect size* estimated allele substitution effect, *SE* standard error of the allele substitution effect, *P-value* nominal significance level of the association, Dosage R^2^ imputation accuracy metric representing the squared correlation between imputed and true genotypes, *Gene* overlapping gene to the SNV, *Variant type* functional type predicted by SNVEff, *Feature ID* Ensembl or RefSeq identifier of the affected transcript or gene, *HGVS.c* cDNA-level variant description (HGVS format), *HGVS.p* Protein-level variant description (HGVS format), *BST* bone structure, *HDE* Heel depth, *LOC* locomotion, *HQU* hock quality, *HO* Holstein, *BS* Brown Swiss

**Fig. 4:**
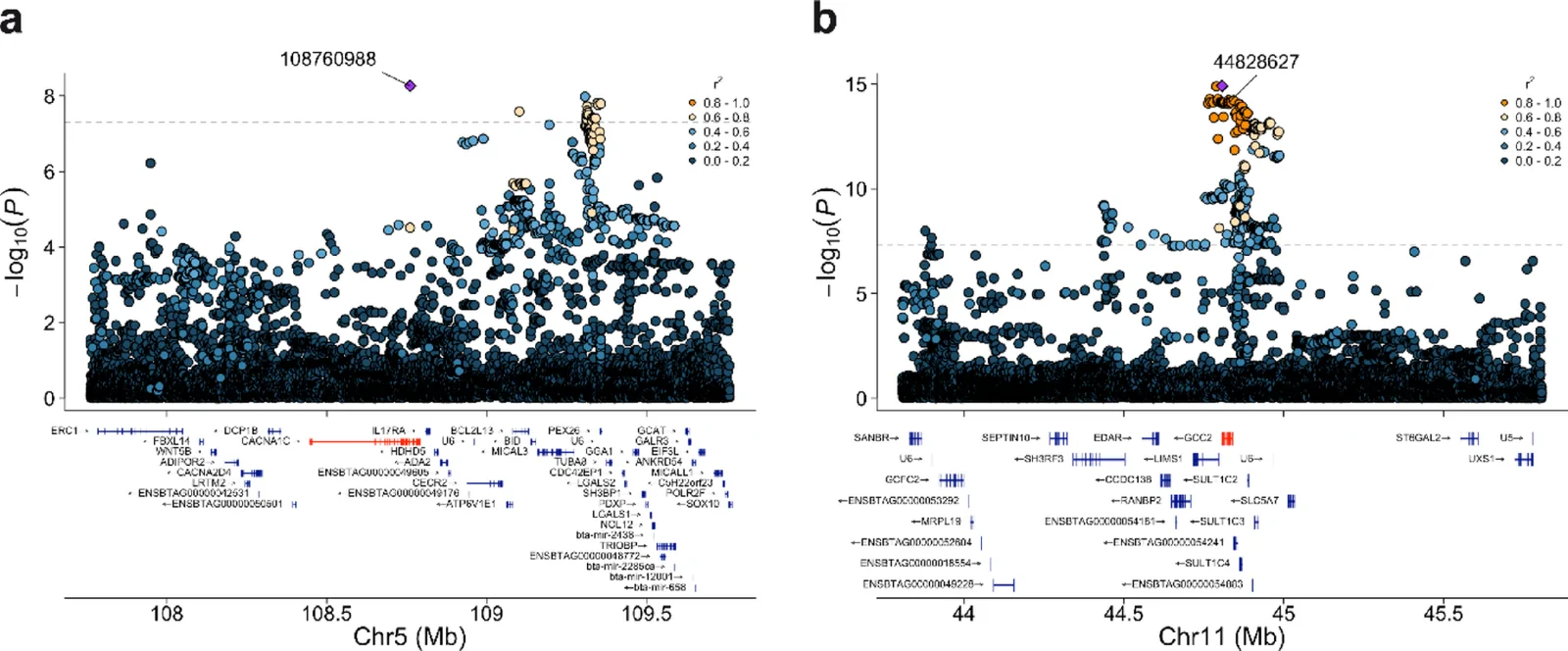
Fine-mapped additive QTLs for locomotion in HO on chromosomes 5 and 11. Regional association plots for LOC in HO within a ± 1 Mb window around the lead SNV. The dot colors indicating linkage disequilibrium (r^2^) relative to the lead SNV. The candidate variants are labeled, and the lead SNVs are shown as a violet diamond. Candidate genes are indicated in red. **a** Chromosome 5 under the additive model displaying candidate variant in *CACNA1C* (chr5:g.108,760,988 T > A). **b** Chromosome 11 under the additive model displaying candidate variant in *GCC2* (chr11:g.44,828,627 T > G)

For LOC in HO, analysis under the additive model revealed two candidate variants (Fig. 4a,b): a *CACNA1C* intron variant at position 108,760,988 bp on chr 5 (*P* = 5.42 × 10^–9^) (Fig. 4a), and a *GCC2* missense variant at position 44,828,627 bp on chr 11 (*P* = 7.31 × 10^–15^) (Fig. 4b).

**Fig. 5:**
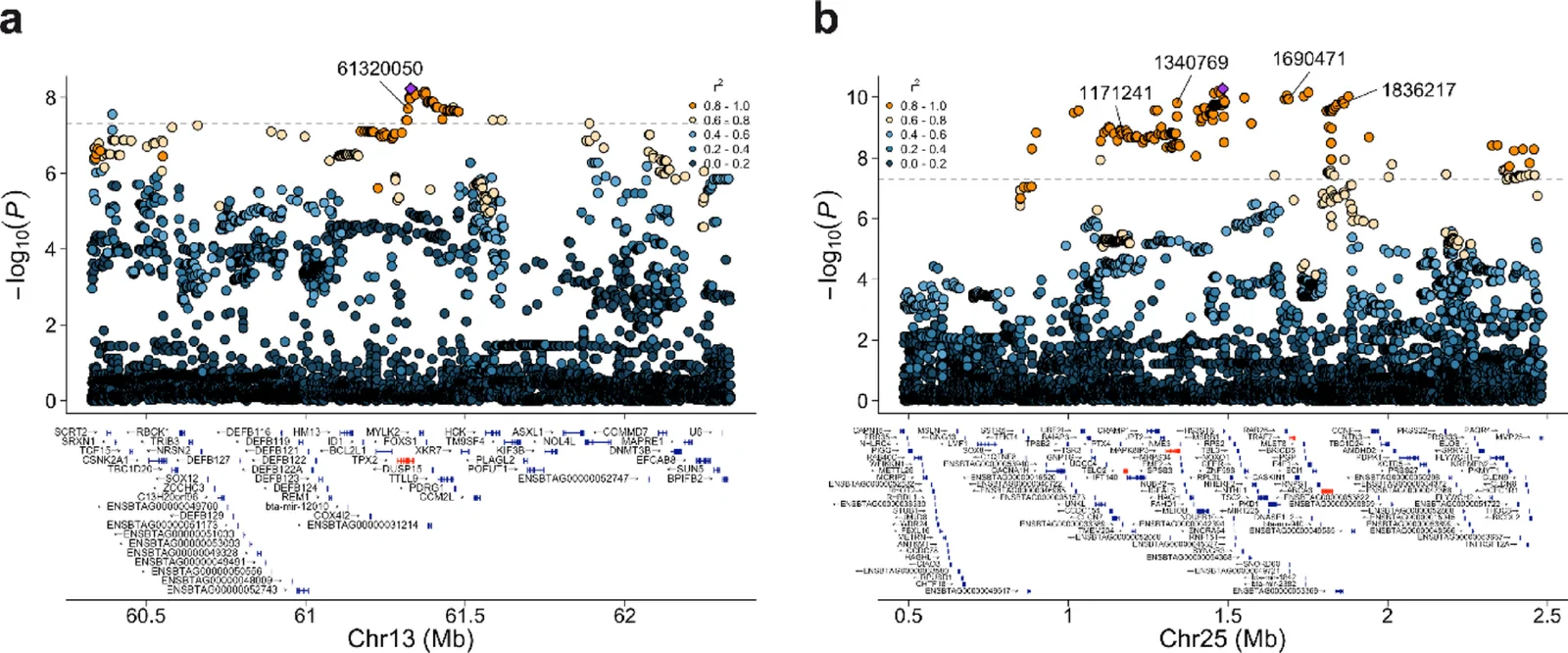
Fine-mapped additive QTL for heel depth in BS on chromosomes 13 and 25. Regional association plots for HDE in BS within a ± 1 Mb window around the lead SNV. The dot colors indicating linkage disequilibrium (r^2^) relative to the lead SNV. The candidate variants are labeled, and the lead SNVs are shown as a violet diamond. Candidate genes are indicated in red. **a** Chromosome 13 under the additive model displaying candidate variant in *TPX2* (chr13:g.61,320,050A > G). **b** Chromosome 25 under the additive model displaying candidate variants in *TELO2* (chr25:g.1,171,241A > C), *MAPK8IP3* (chr25:g.1,340,769C > G), *TRAF7* (Chr25:g.1,690,471 T > G) and *ABCA3* (chr25:g.1,836,217 T > G)

In BS, the additive model identified five candidate variants for HDE (Fig. 5a, b). One was a *TPX2* synonymous variant at 61,320,050 bp on chr 13 (*P* = 1.99 × 10^–8^) (Fig. 5a). Four others were on chr 25 (Fig. 5b): upstream of *TELO2* at position 1,171,241 bp (*P* = 1.55 × 10^–9^), in an intron of *MAPK8IP3* at 1,340,769 bp (*P* = 1.55 × 10^–10^), upstream of *TRAF7* at 1,690,471 bp (*P* = 1.15 × 10^–10^), and upstream of *ABCA3* at 1,836,217 bp (*P* = 2.04 × 10^–10^).

**Fig. 6:**
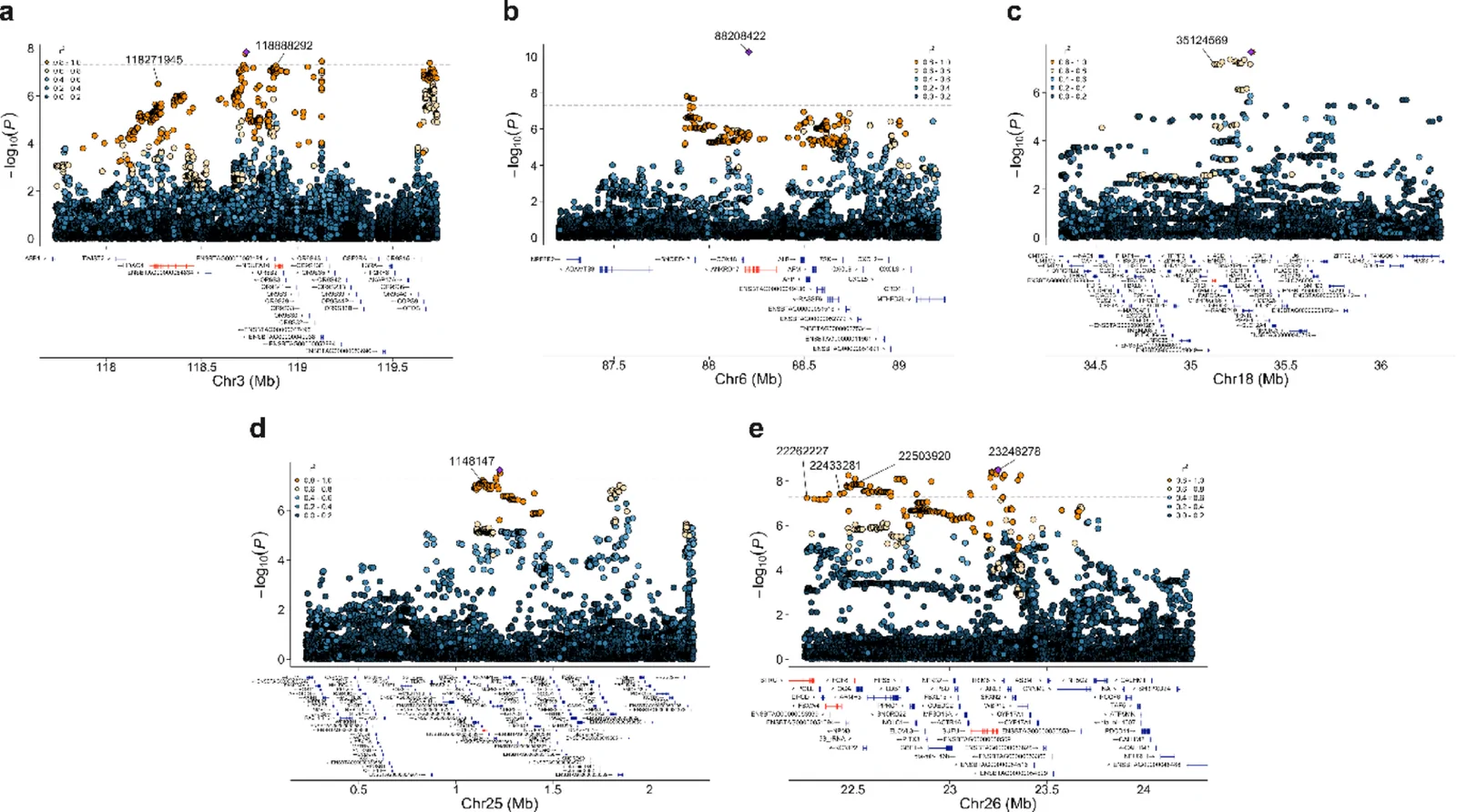
Fine-mapped QTL for hock quality in BS on chromosomes 3, 6, 18, 25, and 26. Regional association plots for HQU in BS within a ± 1 Mb window around the lead SNV. The dot colors indicating linkage disequilibrium (r^2^) relative to the lead SNV. The candidate variants are labeled, and the lead SNVs are shown as a violet diamond. Candidate genes are indicated in red. **a** Chromosome 3 under the additive model displaying candidate variants in *HDAC4* (chr3:g.118,271,945G > A) and *NDUFA10* (chr3:g.118,888,292 T > C). **b** Chromosome 6 under the dominant model displaying candidate variant in *ANKRD17* (chr6:g.88,208,422delTTT). **c** Chromosome 18 under the additive model displaying candidate variant in *RIPOR1* (chr18:g.35,124,569delC). **d** Chromosome 25 under the additive model displaying candidate variant in *CLCN7* (chr25:g.1,148,147G > A). **e** Chromosome 26 under the additive model displaying candidate variants in *BTRC* (chr26:g.22,262,227A > C), *FBXW4* (chr26:g.22,433,281 T > G), *FGF8* (chr26:g.22,503,920G > A), and *SUFU* (chr26:g.23,248,278G > A)

In BS for HQU, nine candidate variants emerged under the additive model (Fig. 6a–e). On chr 3, two candidates, an *HDAC4* intron variant at 118,271,945 bp (*P* = 3.12 × 10^–7^) and an *NDUFA10* intron variant at 118,888,292 bp (*P* = 5.38 × 10^–8^), were prioritized (Fig. 6a). A *RIPOR1* frameshift variant was found on chr 18 at 35,124,569 bp (*P* = 1.44 × 10^–8^) (Fig. 6c). On chr 25, a *CLCN7* synonymous variant located at 1,148,147 bp (*P* = 9.85 × 10^–8^) was selected (Fig. 6d). The variant prioritization for QTL on chr 26 revealed four candidates (Fig. 6e): a *BTRC* intron variant at 22,262,227 bp (*P* = 5.76 × 10^–8^), an *FBXW4* intron variant at 22,433,281 bp (*P* = 3.81 × 10^–8^), an *FGF8* missense variant at 22,503,920 bp (*P* = 1.44 × 10^–8^), and a variant downstream of *SUFU* at 23,248,278 bp (*P* = 3.24 × 10^–9^). Additionally, under the dominant model, an *ANKRD17* intron variant on chr 6 at 88,208,422 bp was noted (*P* = 5.42 × 10^–11^) (Fig. 6b). All fine-mapped candidate variants are listed in Table 3, which reports the estimated allele substitution effect and corresponding standard error, *P*-value, MAF, dosage R^2^, LD (r^2^) with the lead SNV, and the corresponding gene annotation and predicted functional impact.

### Recessive HYAL1 nonsense variant negatively impacts conformation, longevity, and production traits in HO cattle

The *HYAL1* nonsense variant occurred mostly in HO cattle with an allelic frequency of 4.44% in the Swiss HO and 8.37% in the French HO population (Table 4). This variant was found rarely in SI, SF and Vosgienne cattle and was almost absent in many other breeds (Additional file 5, Table S5). Additionally, inspection of the 1000 Bull Genomes Project genomes variant catalogue showed that this variant is almost HO specific (Table 4).

**Table 4:** Occurrence of the recessive *HYAL1* nonsense variant in cattle populations

Cohort	Total	ref/ref	ref/var	var/var	MAF
French HO	1,159,426	973,329	178,145	7952	0.084
Swiss HO	923	842	80	1	0.044
SF	140	138	2	0	0.007
Vosgienne	4816	4746	70	0	0.007
SI	713	712	1	0	0.001
Global cohort*	5055	4887	161**	7***	0.017

*1000 Bull Genomes Project Cohort

**147 Holstein

***All Holstein

ref/ref, homozygous for the reference allele

ref/var, heterozygous

var/var, homozygous for the alternative allele

*MAF* frequency of the alternative allele, *HO* Holstein, *Global* 1000 bulls genome cohort, *SF* Swiss Fleckvieh, *SI* Simmental

**Fig. 7:**
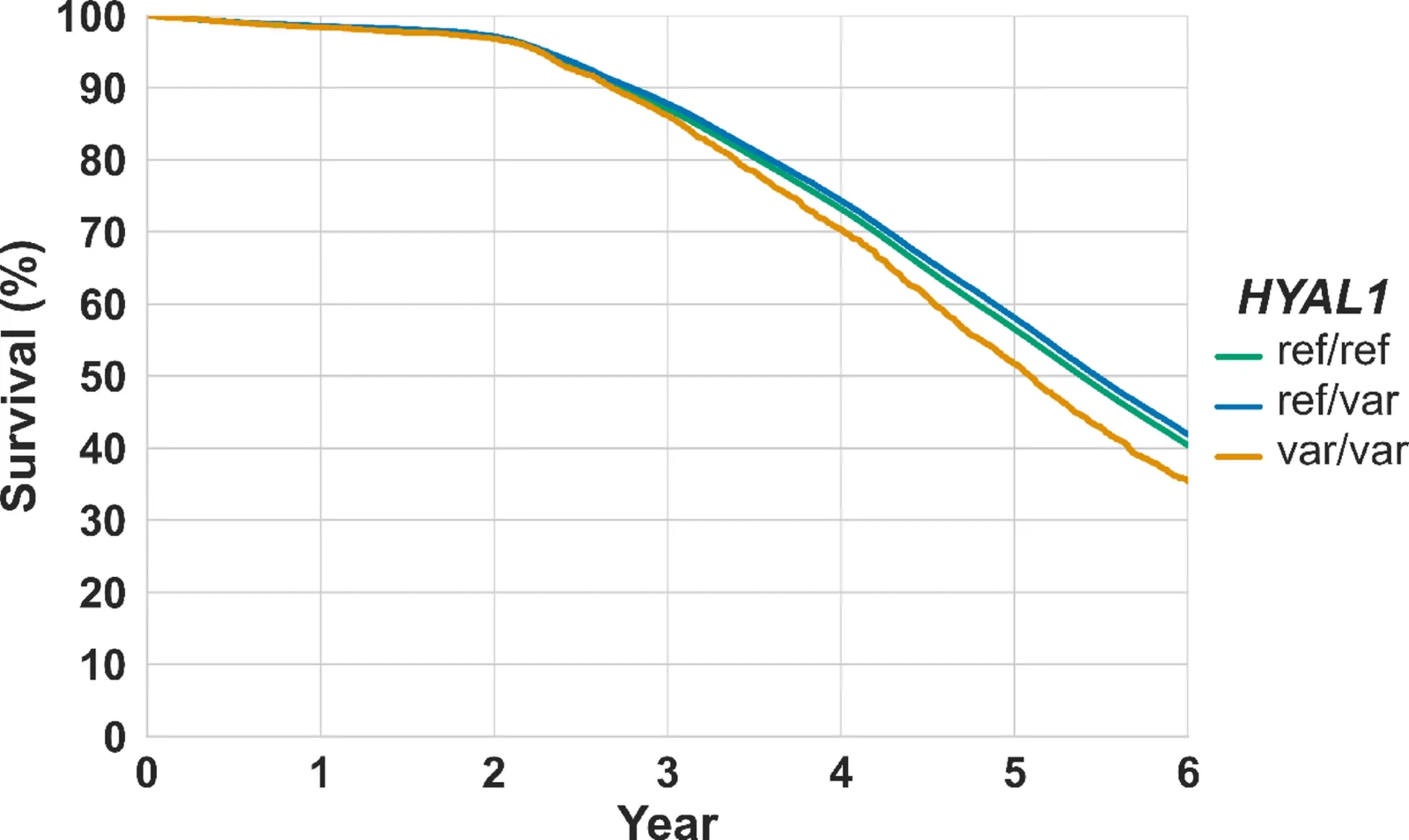
Six-year survival curves of French HO females by *HYAL1* genotype. Survival curves over 0–6 years for French HO females stratified by *HYAL1* genotype, showing reduced survival of var/var animals from ~ year 3 onward

The six-year survival analysis performed in the French HO population considering the different *HYAL1* genotypes showed that var/var animals had a significantly shortened lifespan from three years of age and older (Table 5, Fig. 7; Additional file 6, Table S6).

**Table 5:** Proportion of French HO females alive at year-end by *HYAL1* genotype over six years

Year	Total	ref/ref	ref/ref (%)	ref/var	ref/var (%)	var/var	var/var (%)	P-value
0	328,516	271,211	100	54,629	100	2665	100	NA
1	323,837	267,369	98.58	53,846	98.57	2622	98.39	0.66
2	318,840	263,193	97.04	53,067	97.14	2580	96.81	0.32
3	286,334	236,091	87.05	47,948	87.77	2295	86.12	9.99 × 10^–6^
4	240,856	198,407	73.16	40,576	74.28	1873	70.28	4.99 × 10^–6^
5	186,259	153,167	56.48	31,714	58.05	1378	51.71	4.99 × 10^–6^
6	133,118	109,329	40.31	22,847	41.82	942	35.35	4.99 × 10^–6^

ref/ref, homozygous for the reference allele

ref/var, heterozygous

var/var, homozygous for the alternative allele

Total, total number of females alive at the end of the corresponding year of life

*P*-value, Fisher’s exact test with Monte Carlo *P*-value estimation

Additionally, significant pleiotropic effects of the genotype in var/var *HYAL1* French HO animals were found for 14 out of 34 routinely recorded production and conformation traits that were available (Table 6; Additional file 7, Table S7). The strongest impacts were observed for LOC and RLV (− 0.96 and − 0.75 genetic standard deviations, respectively).

**Table 6:** Pleiotropic significant effects of the *HYAL1* homozygous (var/var) French HO females on production and confirmation traits

Trait	Trait GSD	Effect GSD	SE Effect GSD	q-value
FAN*	0.315	0.287	0.110	4.67 × 10^–2^
LOC*	0.317	− 0.956	0.110	1.58 × 10^–13^
RLR*	0.435	− 0.746	0.110	9.14 × 10^–9^
Rump angle*	0.504	0.186	0.062	2.39 × 10^–2^
Rump width*	0.604	− 0.30	0.064	1.53 × 10^–4^
Chest width*	0.453	0.287	0.081	4.49 × 10^–3^
Stature*	1.053	− 0.268	0.049	1.59 × 10^–5^
Fore udder attachment*	0.695	0.303	0.070	6 × 10^–4^
Udder cleft*	0.705	− 0.238	0.074	1.33 × 10^–2^
Udder balance*	0.434	0.253	0.076	9.5 × 10^–3^
Teat length*	0.757	− 0.329	0.057	2.85 × 10^–6^
Milk yield**	758.578	− 0.299	0.032	1.05 × 10^–13^
Milk fat yield***	31.205	− 0.255	0.033	2.93 × 10^–10^
Milk protein yield***	22.766	− 0.286	0.032	1.15 × 10^–12^

*n = 1030, number of *HYAL1* genotyped animals

**n = 3416, number of *HYAL1* genotyped animals

***n = 3409, number of *HYAL1* genotyped animals

*FAN* foot angle, *LOC* locomotion, *RLR* rear leg rear view, *Trait GSD* genetic standard deviation of the trait from the national genomic evaluation parameters, *Effect GSD* effect expressed in genetic standard deviation units, *SE Effect GSD* effect expressed in genetic standard deviation units

q-value, Benjamini–Hochberg false discovery rate–adjusted p-value

### HYAL1 nonsense variant associated with a recessive form of polysynovitis in HO cattle

Experimental PCR-based genotyping confirmed the *HYAL1* Gln93* homozygosity in all seven predicted var/var carriers. Clinically, all animals exhibited hock enlargement bilaterally involving multiple joints, including the tibio-tarsal, proximal intertarsal, distal intertarsal, and tarsometatarsal joints (Fig. 3d). On palpation in cases 1 to 6 these joints were enlarged, soft, non-warm, and non-painful, and with decrease range of motion consistent with joint effusion. Case 7, in addition to the affected hock joints, presented enlargement of the metacarpophalangeal/metatarsophalangeal (fetlock) joints bilaterally (Fig. 3e) and displayed lameness (score 3 out of 5). In this animal, the affected hock and fetlock joints were enlarged, soft, non-warm, but painful on palpation and with marked decrease range of motion. The remainder of the physical examination was unremarkable. Ultrasonography of the affected joints revealed abundant anechogenic content within the joint cavities in all animals, consistent with synovial effusion (Fig. 3f). The joint capsules appeared distended, and the synovial lining was mildly thickened. The clinical and ultrasonographic findings were consistent with a novel form of polysynovitis involving effusion in the hock joints.

## Discussion

This study provides the first high-resolution characterization of the genetic architecture of hoof and leg conformation traits in Swiss HO and BS cattle, which have low to moderate heritabilities. This was achieved by integrating imputed WGS data, additive and non-additive GWAS models, and Bayesian fine-mapping. Using the additive model, we identified multiple QTL characterised by polygenic signals of small effect, while non-additive analyses revealed a candidate non-additive, recessive *HYAL1* nonsense variant on chr 22 in HO for BST. Extensive genotyping data from the French HO population demonstrated that the *HYAL1* variant is associated with reduced survival from the age of three years and has pleiotropic negative effects on other traits, including hoof and leg conformation traits such as FAN, LOC and RLR. Clinical assessment of var/var HO females further corroborated the association between the *HYAL1* nonsense variant with clinical findings consistent with a novel form of polysynovitis in the hock joints. Together, these results demonstrate the value of combining dense genomic data with multiple inheritance models to analyse hidden Mendelian single-gene disorders and contribute to our understanding of the complex genetic basis of hoof and leg conformation traits.

Across traits and populations, genomic ℎ^2^ estimates ranged from low to moderate and were largely consistent between Swiss HO and BS cattle. In HO, the highest ℎ^2^ was observed for BST, whereas in BS it was observed for HQU. While these are not directly equivalent traits, both reflect structural aspects of limb conformation within their respective breed-specific scoring schemes: BST describes limb fineness, whereas HQU describes the hock joint. In contrast, HDE consistently exhibited low ℎ^2^ in both populations. FAN and rear leg traits showed moderate ℎ^2^ with similar estimates across breeds. Overall, the ℎ^2^ estimates obtained in this study were supported by their consistency with national genetic evaluations and fall within the ranges previously reported in dairy cattle [6, 12, 16, 22, 23, 28, 74–77], further supporting the validity of the phenotype correction approach.

For LOC in HO cattle, we identified two QTL on chr 5 and chr 11. The chr 5 locus mapped to 107.8–109.8 Mb and overlaps with regions previously reported for hoof and leg conformation traits. Abdalla et al. [16] reported a QTL for RLS at approximately 108.2 Mb in Chinese HO and proposed *ADIPOR2* as a candidate gene, and this association was also reported by Long et al. [22]. In addition, Wu et al. [23] detected a signal for overall conformation score at 110.15 Mb and highlighted *ANKRD54*. At this locus, we prioritised an intronic variant in *CACNA1C*, which encodes the L-type calcium channel Cav1.2 involved in excitation–contraction coupling and neuromuscular signalling [78, 79]. Pathogenic variants in *CACNA1C* cause Timothy syndrome in humans (OMIM 601005), a congenital multisystem disorder characterised by syndactyly, cardiac disease, immune deficiency, hypoglycemia, and cognitive abnormalities [80]. Given its essential role in skeletal muscle contraction and neuronal excitability, *CACNA1C* therefore represents a functional plausible candidate for bovine LOC-related traits. The second LOC-associated region was located on chr 11, where we identified a candidate missense variant in *GCC2* at 44.8 Mb. *GCC2* encodes a Golgi-associated protein involved in vesicular trafficking and Golgi organisation [81]. This gene is listed in aggregated disease databases among genes associated with Charcot-Marie-Tooth disease, axonal, type 2Z, a human hereditary peripheral neuropathy characterised by distal lower-limb weakness and impaired perception [82, 83]. In addition, *GCC2* is moderately expressed in limb muscle and bone in adult mice [84]. Taken together, these observations provide supportive evidence for considering *GCC2* as a candidate gene for LOC in HO cattle.

In HO cattle, several BST-associated regions were detected under the additive model on chr 5, 6, 7, 14 and 18. Fine-mapping of the additive BST signals did not yield candidate variants meeting our criteria, as no SNV passed all filtering steps. In several regions, variants with strong association signals and high LD with the lead SNV were present, but were not retained because they were not included in the credible set, indicating a diffuse association signal rather than a single high-confidence candidate. In other cases, the LD or association thresholds were not met. Nevertheless, several of these regions overlap with previously reported QTL for hoof and leg conformation traits, supporting their biological relevance at the locus level. The region on chr 5 (~ 104.2–105.8 Mb) corresponds to a QTL reported by Sousa Junior et al. [6] in Canadian HO, who identified a LOC-associated variant at 105.74 Mb and highlighted *FGF23*, a gene involved in phosphate homeostasis and bone mineralisation [85]. The region on chr 7 (~ 90.8 Mb) is located in proximity to a QTL identified by Abo-Ismail et al. [25] at 93.24 Mb, where *ARRDC3* was proposed as a candidate gene for BST and chest width. *ARRDC3* has previously been associated with growth- and conformation-related traits in cattle [86, 87]. Variants in LD with this region showed strong association signals in our data, including a variant at 90.86 Mb, although no variant within *ARRDC3* was retained in the credible set. The region on chr 14 (22.82–24.33 Mb) lies close to a QTL reported by Abdalla et al. [16] at 22.61 Mb associated with BST, where *XKR4* was proposed as a candidate gene and has been previously linked to growth-related traits in cattle [88]. Taken together, these results indicate that BST is influenced by multiple genomic regions with consistent signals across studies, but without a single additive high-confidence causal variant, supporting a polygenic architecture characterised by multiple small-effect loci.

For HDE in BS cattle, we identified a candidate additive *TPX2* synonymous variant on chr 13 and a cluster of additive *TELO2*, *MAPK8IP3*, *TRAF7* and *ABCA3* non-coding variants on chr 25. *TPX2* plays a central role in cell proliferation through its involvement in mitotic spindle assembly and is expressed in developing limb tissues [84, 89], supporting a potential contribution to distal limb morphology. The QTL on chr 25 comprises genes involved in growth signalling, neuronal transport and limb development. Specifically, *TELO2* contributes to growth-factor signalling and normal development [90, 91], *MAPK8IP3* is required for axonal transport [92] and has been linked to neurodevelopmental disorders in humans [93], and *TRAF7* regulates NF-κB and MAPK pathways [94] and is associated with distal limb malformations [95]. Although *ABCA3* is best known for its role in pulmonary function [96], it is also expressed during limb development [84]. In summary, these genes converge on pathways that are involved in tissue growth, the patterning of limbs, and the development of the neuromuscular system. This supports the idea that this genomic region could contribute to variation in HDE in a biologically plausible way.

In BS cattle, nine candidate variants associated with HQU were identified under the additive and dominant models, encompassing both coding and regulatory regions. The *HDAC4* and *NDUFA10* intron variants are both located within a previously reported leg conformation QTL on chr 3 in BS [28]. *HDAC4* is a key regulator of chondrocyte hypertrophy and endochondral ossification [97], and altered dosage causes skeletal abnormalities in humans and mice [98, 99], supporting its relevance for joint morphology underlying HQU in cattle. *NDUFA10*, which encodes a mitochondrial complex I subunit, has been linked to neuromuscular and movement disorders in humans [100, 101], suggesting that changes in cellular energy metabolism may influence phenotypic expression of HQU in cattle. An additional candidate non-additive, dominant *ANKRD17* variant was identified in the QTL found on chr 6. In humans, heterozygous loss-of-function variants in this gene cause a neurodevelopmental syndrome (OMIM 619504) characterised by gait instability and joint hypermobility [102], suggesting a possible involvement in hock soft tissue integrity and LOC also in cattle. On chr 18, a *RIPOR1* loss-of-function variant was identified as a strong candidate additive variant for HQU. *RIPOR1* regulates RhoA–actin signalling [103, 104], a pathway essential for osteoclast function and bone remodelling [105], linking cytoskeletal organisation and bone turnover to hock joint structure. A *CLCN7* synonymous variant maps to a region on chr 25 that has previously been reported to be associated with body and leg conformation in BS [28]. The encoded chloride channel protein is essential for osteoclast-mediated bone resorption. Pathogenic *CLCN7* variants cause different forms of osteopetrosis in humans (OMIM 602727) and cattle (OMIA 001887–9913) [106–108], which support the importance of this gene in shaping joint morphology. Finally, a cluster of variants in *BTRC*, *FBXW4*, *FGF8* and *SUFU* on chr 26 affect candidate genes with well-established but distinct roles in limb development and skeletal patterning [109–116]. Fang and Pausch (2019) mapped a QTL for leg conformation to this same chr 26 region and proposed *BTRC* as a candidate gene. *BTRC* encodes the F-box protein β-TrCP, a regulator of Wnt/β-catenin and NF-κB pathways involved in limb and skeletal development [109–111]. In humans, expression analyses at the *SHFM3* locus show that *SUFU* is overexpressed together with *BTRC* in patients with split hand/foot malformation carrying 10q24 duplications [110]. Among the prioritised variants at this locus, the *FGF8* missense variant was the only protein-changing variant. *FGF8* encodes a key apical ectodermal ridge morphogen required for limb-bud outgrowth and proximodistal patterning [117], and inactivation of *Fgf8* in mouse limbs causes severely shortened or absent limb elements [118]. Taken together, the presence of multiple developmentally relevant genes, the previously reported leg conformation QTL in BS, and the prioritisation of a missense variant in *FGF8* support the chr 26 region as a biologically plausible locus influencing HQU.

The *HYAL1* nonsense variant, which is associated with the non-additive QTL for BST, was largely confined to the HO background. Its widespread occurrence was confirmed in both Swiss and French HO populations, but it was almost absent in other Swiss and French breeds. Furthermore, independent evidence suggests that var/var carriers experience unfavourable consequences. In the French HO population, *HYAL1* homozygotes exhibited reduced longevity and consistent detrimental effects across various production and conformation traits, including impaired LOC, adverse rear-leg conformation, and decreased milk production. Importantly, the observed reduction in survival from approximately three years of age may reflect early culling at the end of the first lactation rather than later-life mortality. This interpretation is consistent with standard dairy management practices, as HO cows typically calve for the first time at around two years of age, followed by a 305-day lactation period, after which animals with impaired functional traits are commonly removed from the herd. Together, these findings support a pleiotropic effect of the *HYAL1* variant, influencing both structural soundness and productive performance and thereby increasing the risk of early culling. This prompted an examination of the underlying clinical phenotype and possible biological mechanism. A targeted clinical examination of var/var animals revealed signs consistent with polysynovitis. Interestingly, *HYAL1* encodes a lysosomal hyaluronidase, which plays a pivotal role in hyaluronan degradation and joint homeostasis [119]. In humans, pathogenic *HYAL1* variants cause mucopolysaccharidosis type IX (OMIM 601492), a recessively inherited disorder characterised by recurrent joint effusion, proliferative synovitis and periarticular soft-tissue masses [119–121]. In mouse models, disruption of *Hyal1* leads to reduced bone mineral density, shorter long bones and altered hyaluronan turnover, further supporting its role in skeletal integrity [122]. Although this gene has not previously been associated with bovine conformation traits, studies have reported its contribution to reproductive performance in Nellore cattle [123] and identified signs of positive selection in Zaosheng cattle [124], which supports its biological relevance in cattle populations. Taken together, these findings provide the first evidence of a *HYAL1* variant that is most likely pathogenic and associated with BST in HO cattle. The combination of genetic evidence and consistent clinical abnormalities in homozygous affected HO cattle strongly suggests that impaired hyaluronan degradation disrupts the regulation of joint fluid and compromises the maintenance of joint tissues. Chronic joint alterations of this kind are expected to reduce structural stability and increase susceptibility to lameness, which has negative consequences for LOC, longevity, and overall productivity, particularly under housing conditions that impose high mechanical stress on joints [125].

Although the significant results presented addressed the aims of our study, we are aware of several limitations. First, HDE was measured in millimetres, whereas the remaining hoof and leg conformation traits were scored on a 1–9 scale. Because each trait was analysed separately, this difference in measurement scale is unlikely to have affected the detection of significant associations. However, the absolute magnitude of SNV effect estimates should not be directly compared across traits measured on different scales. In addition, our GWAS used residuals from MLMA fitted with lme4 to treat these ordinal scores as continuous variables for association testing. While linear models are computationally attractive for large-scale genomic analyses, they do not fully reflect the discrete, ordered nature of the underlying scale [126]. Threshold models are theoretically more appropriate for ordinal traits and are widely used for categorical traits in dairy cattle [127]. In addition, nuisance effects such as age and lactation stage were modelled as fixed categorical factors. Random regression approaches using Legendre polynomials are commonly applied to model longitudinal trajectories across lactation and could provide a more flexible correction for non-linear time-dependent effects. This would reduce residual variance and improve the ability to detect subtle genetic signals [128]. An additional limitation is that the association analyses based on imputed WGS variants were restricted to chromosomal regions identified in the HD-subset GWAS and were not performed genome-wide. Therefore, additional association signals that were not captured or did not reach suggestive significance in the HD-subset analysis may have been missed. The Bayesian fine-mapping analysis with PAINTOR also involved several methodological compromises. Firstly, the analysis was restricted to 2 Mb windows to focus on the local signal, capture SNVs in strong LD with the lead SNV, and reduce noise from more distant SNVs, which could introduce unrelated patterns and complicate the prioritisation of local candidate variants. However, given the extensive LD structure in cattle, this window size may still have excluded relevant variants located just outside the predefined boundaries [129, 130]. The algorithm's high computational intensity further supported the use of a restricted window size. Secondly, PAINTOR is designed to integrate functional annotation and to improve prioritisation of candidate causal variants. Although FAETH scores were used as one of the priors, FAETH annotations are only available for around 17 million sequence variants. In contrast, our imputed datasets contained more SNVs [63]. Consequently, some biologically relevant variants likely lacked FAETH scores and were given less weight than fully annotated variants, which could have biased credible sets towards well-annotated SNVs. Beyond the fine-mapping step, another methodological limitation concerned the handling of multiple genetic models in the GWAS analysis. We used GCTA-MLMA, which required separate additive, dominant and recessive analyses [59]. In regions where additive and non-additive signals overlapped, prioritising the additive model for interpretation may have biased the inference against loci where dominance or recessively would be more biologically appropriate. A more principled approach would involve MAX-type tests across additive, dominant and recessive coding [131], or a mixed model that estimates additive and dominance effects jointly [132]. Finally, the detection of non-additive effects remains challenging in general, particularly for recessive signals that require enough homozygous minor-allele carriers. Furthermore, ascertainment bias may further reduce detectability if severely affected animals are excluded from the study due to prior selection, resulting in the lower phenotypic tail being censored. Although the association between the *HYAL1* nonsense variant and polysynovitis was supported by targeted clinical examinations, the validation cohort comprised only seven var/var females, which limited the precision of penetrance and phenotypic variability. Further follow-up studies including heterozygous carriers are required to characterise the spectrum of lameness risk over time. Similarly, the remaining 16 candidate causal variants prioritised by our fine-mapping approach rely on statistical and in silico evidence. Extended genotyping and multi-layered functional follow-up will be required for validation.

## Conclusions

This study provides the first high-resolution characterization of the genetic architecture underlying hoof and leg conformation traits in Swiss HO and BS cattle using both additive and non-additive GWAS models. We demonstrate that these traits are highly polygenic, with low-to-moderate heritability and contributions from numerous loci of small-to-moderate effect. We prioritised 17 candidate causal variants across 15 QTL through the integration of imputed WGS data with Bayesian fine-mapping. This highlighted additive protein-changing as well as non-coding variants in different functionally relevant genes.

Importantly, modelling non-additive genetic effects using proxy phenotypes revealed a recessive *HYAL1* nonsense variant associated with bone structure in HO cattle, which would have gone unnoticed under standard additive GWAS. Homozygous carriers exhibited markedly reduced survival from the age of three and pleiotropic effects on locomotion and rear leg conformation, as well as broader impacts on production traits. Therefore, mating of carriers should be avoided to prevent further losses. Independent clinical validation in HO confirmed this candidate variant as the harmful recessive allele underlying polysynovitis and joint effusion. This establishes a direct mechanistic link between genotype, pathology, performance, and longevity.

## Supplementary Information

Supplementary Material 1.

Supplementary Material 2.

Supplementary Material 3.

Supplementary Material 4.

Supplementary Material 5.

Supplementary Material 6.

Supplementary Material 7.

Supplementary Material 8.

## Data Availability

Imputed whole-genome sequence genotypes used in this study were generated using reference panels from the Animal Genomics group at ETH Zürich and the 1000 Bull Genomes Project, as detailed in the Methods section. Phenotypic records from routine linear scoring were provided by the breeding organisations Swissherdbook, Holstein Switzerland, and Braunvieh Switzerland.
