# Supplementary material for: Genomic dissection of hoof and leg conformation in Swiss dairy cattle populations reveals polygenic architecture and a recessive HYAL1 nonsense variant affecting longevity in Holstein cattle
Source: Genet Sel Evol. 2026 Jul 5;58:46. doi: 10.1186/s12711-026-01068-3 (PMC13491847; doi:10.1186/s12711-026-01068-3)

Additional file 4, Figure S1. Regional association plot and genotype-wise phenotype distribution for selected markers in LOC (HO), chromosome 5, under the additive model.

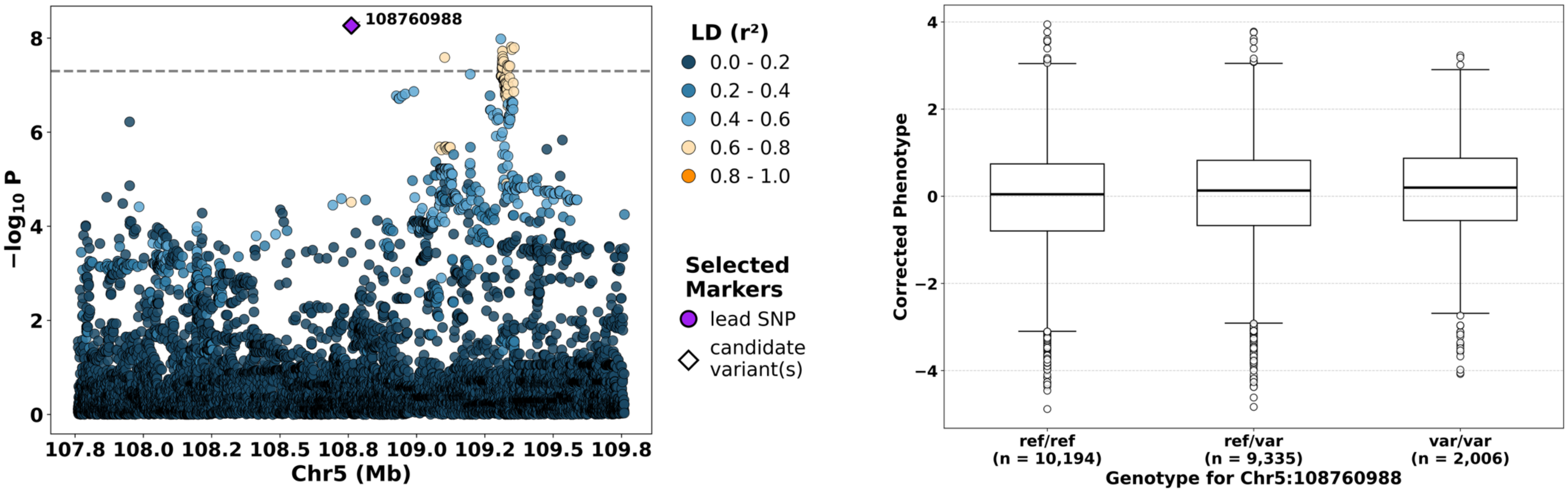

Additional file 4, Figure S2. Regional association plot and genotype-wise phenotype distribution for selected markers in LOC (HO), chromosome 11, under the additive model.

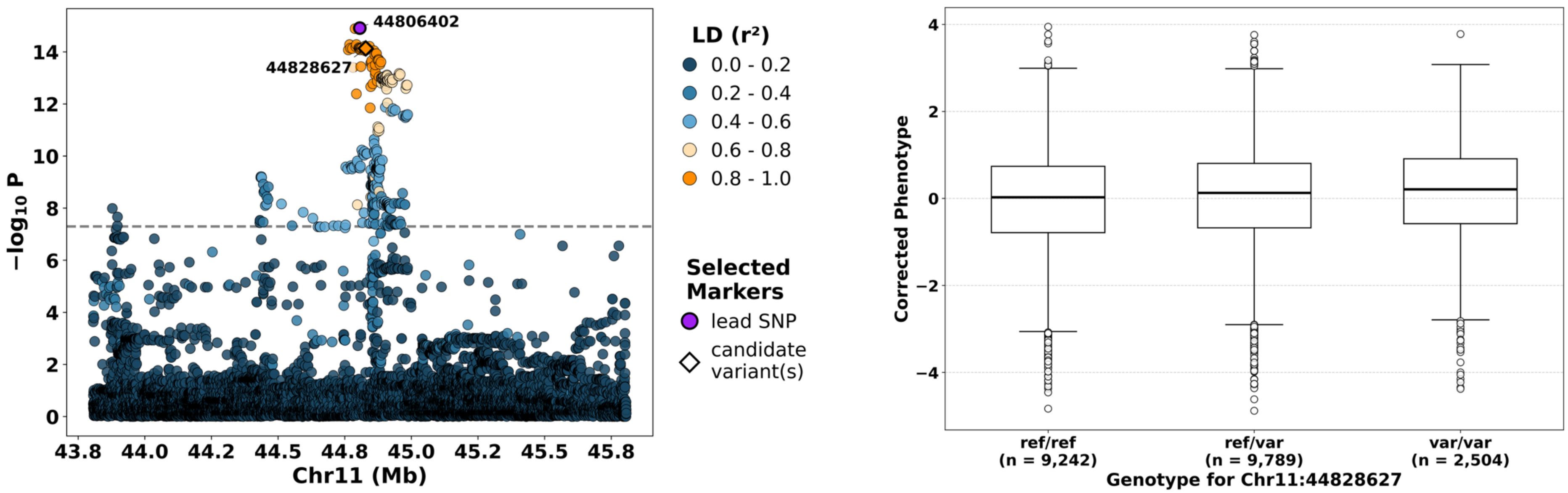

Additional file 4, Figure S3. Regional association plot and genotype-wise phenotype distribution for selected markers in LOC (HO), chromosome 11, under the recessive model.

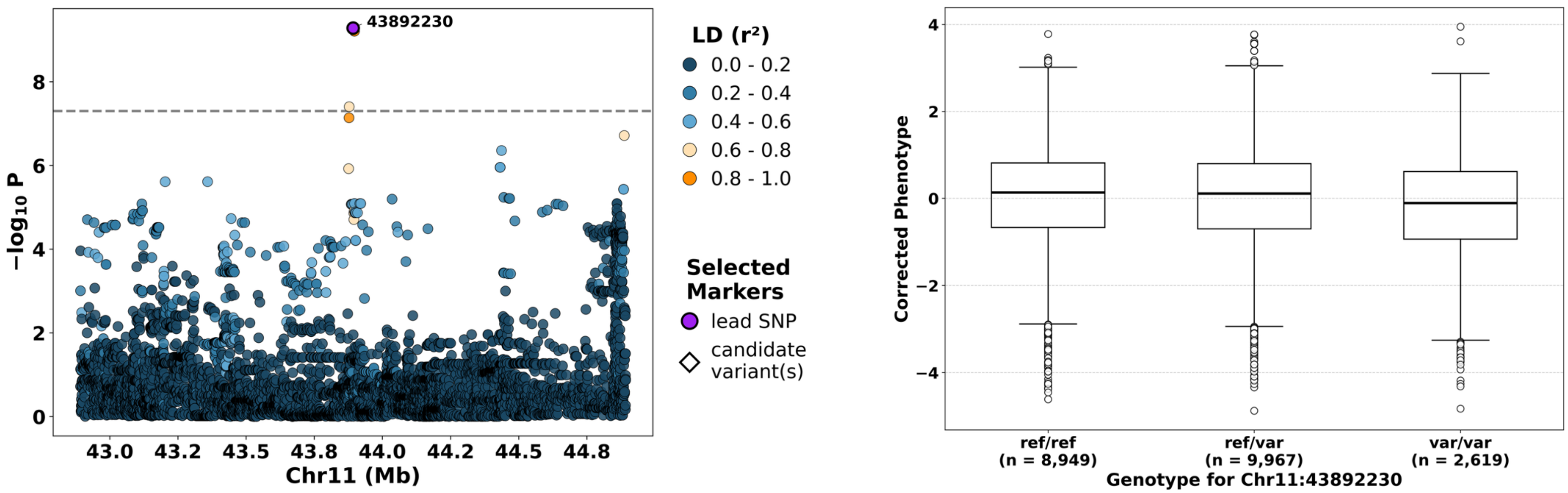

Additional file 4, Figure S4. Regional association plot and genotype-wise phenotype distribution for selected markers in LOC (HO), chromosome 5, under the dominant model.

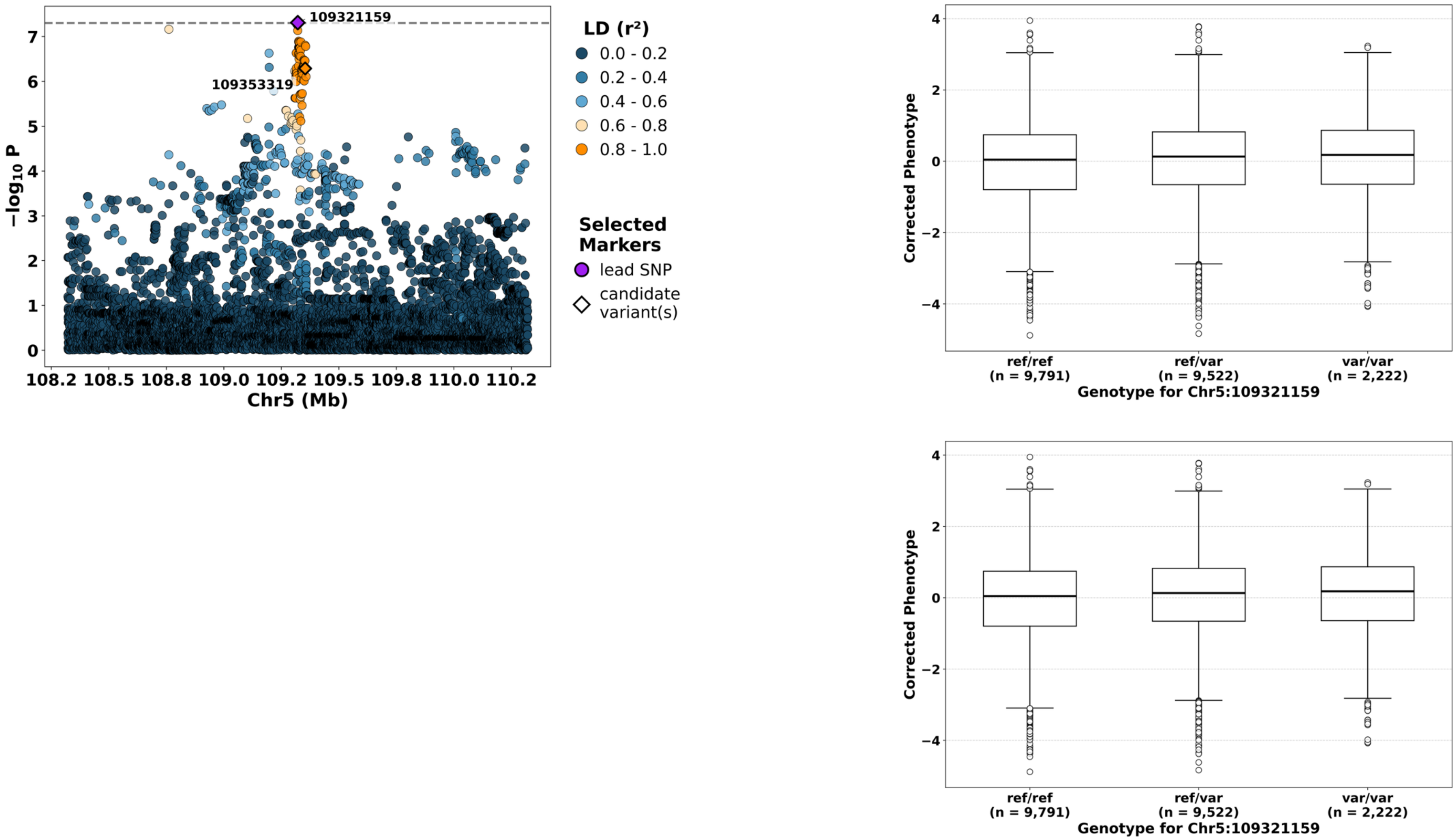

Additional file, Figure S5. Regional association plot and genotype-wise phenotype distribution for selected markers in BST (HO), chromosome 5, under the additive model.

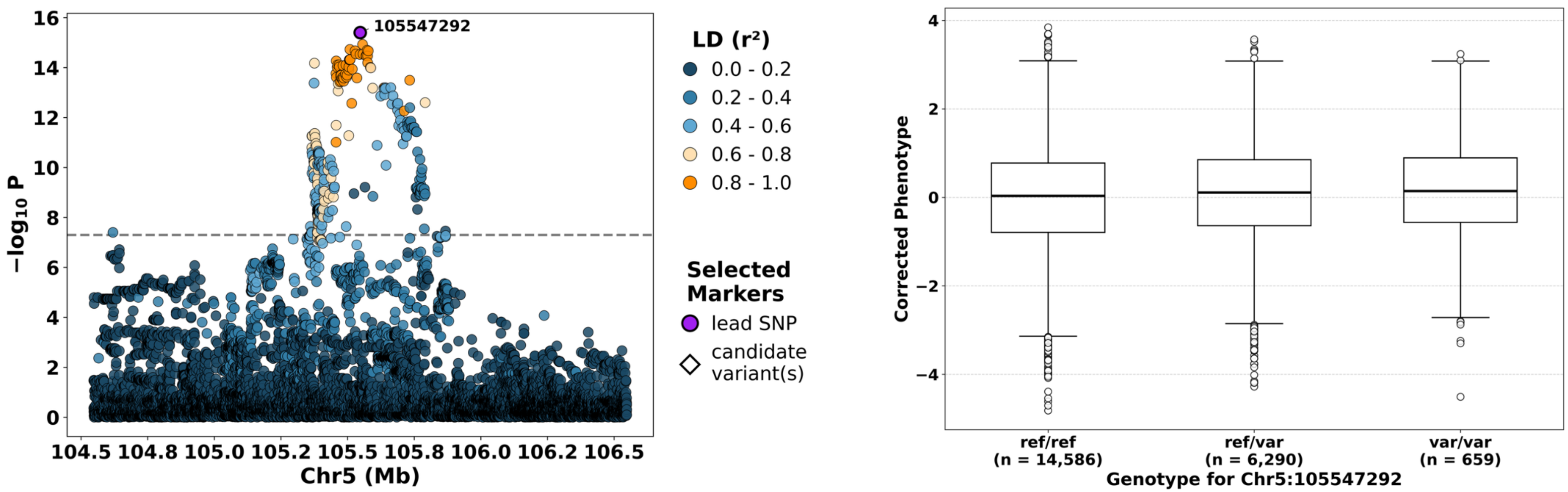

Additional file 4, Figure S6. Regional association plot and genotype-wise phenotype distribution for selected markers in BST (HO), chromosome 6 for the primary independent signal, under the additive model.

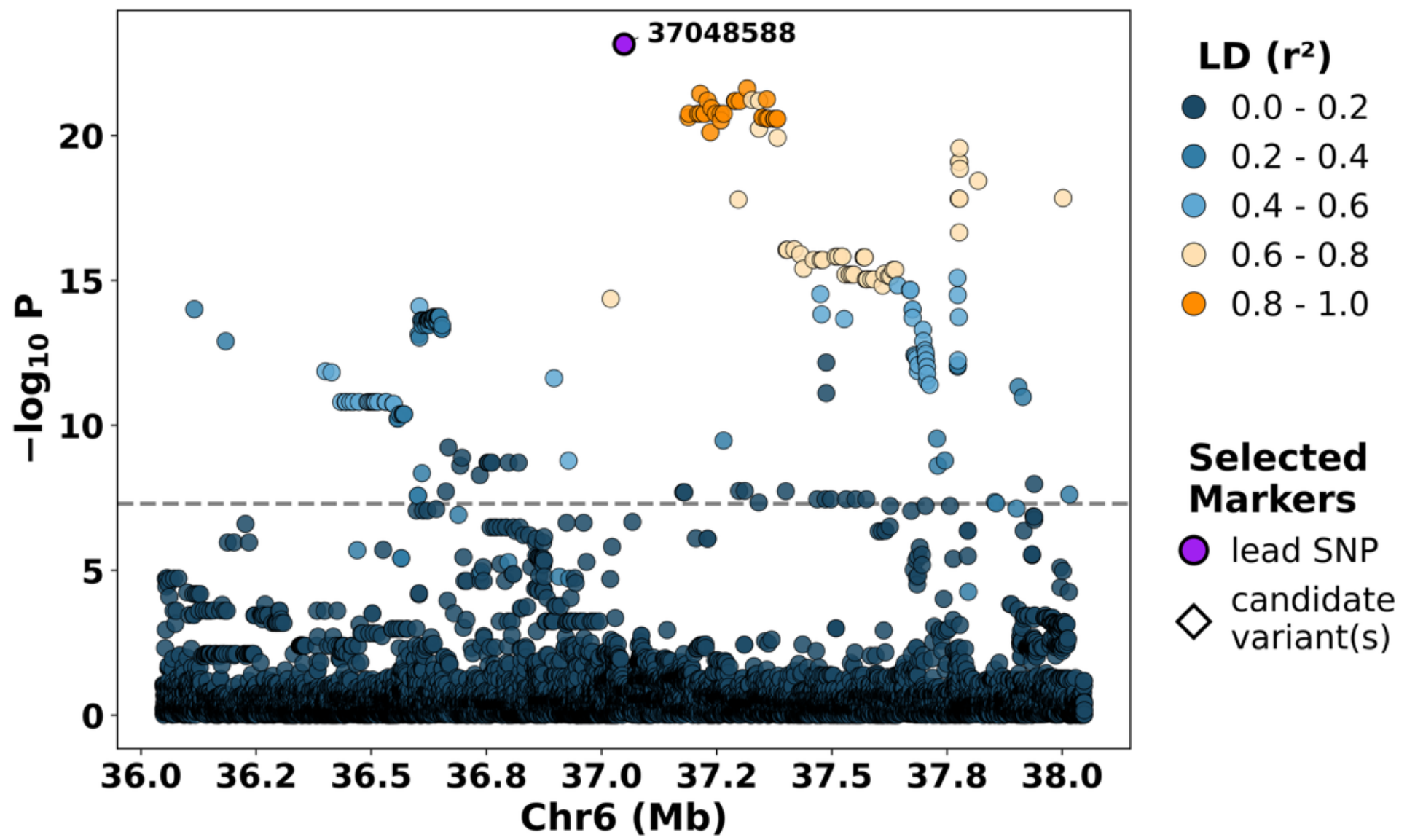

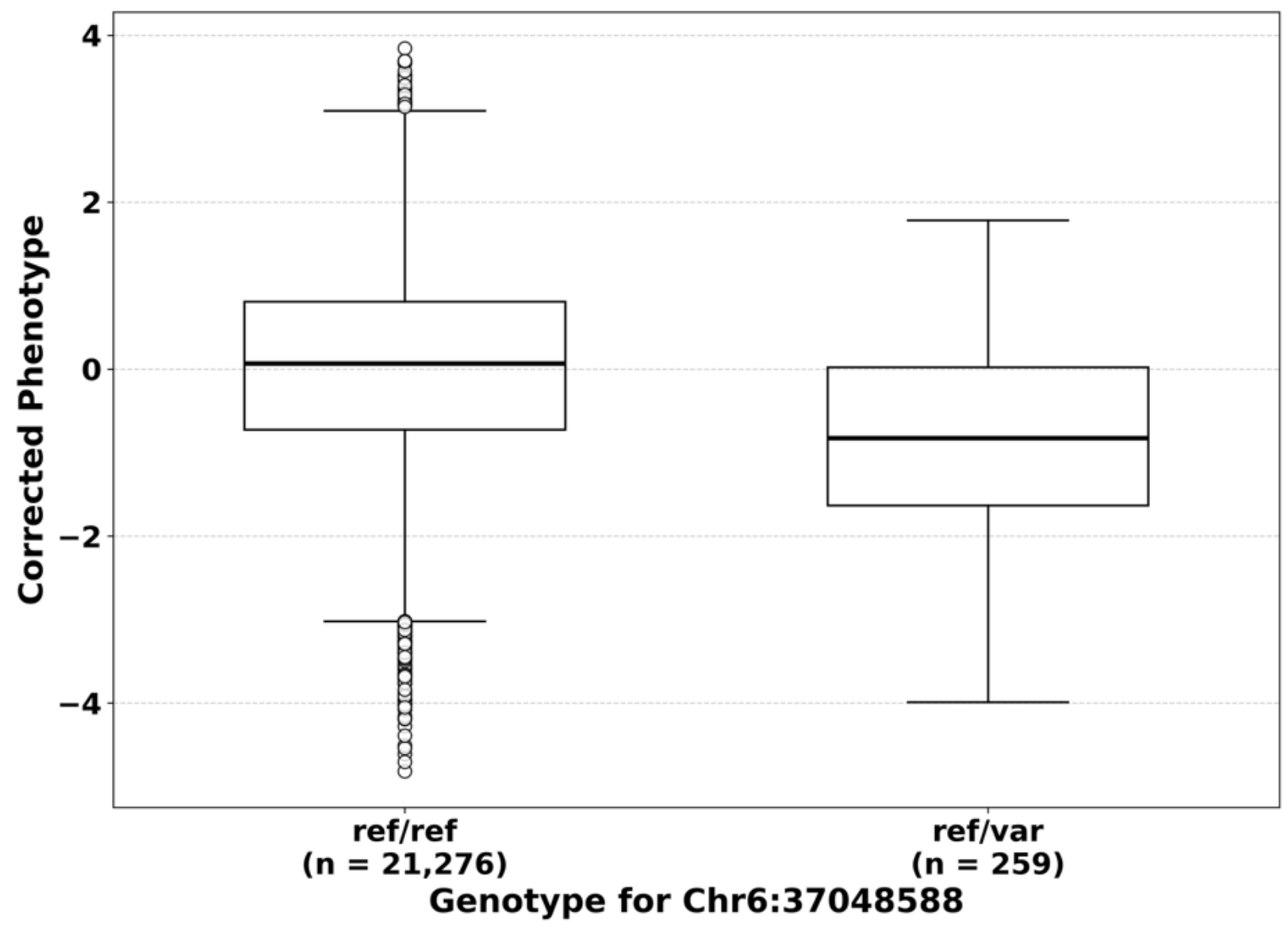

Additional file 4, Figure S7. Regional association plot and genotype-wise phenotype distribution for selected markers in BST (HO), chromosome 6 for the secondary independent signal, under the additive model.

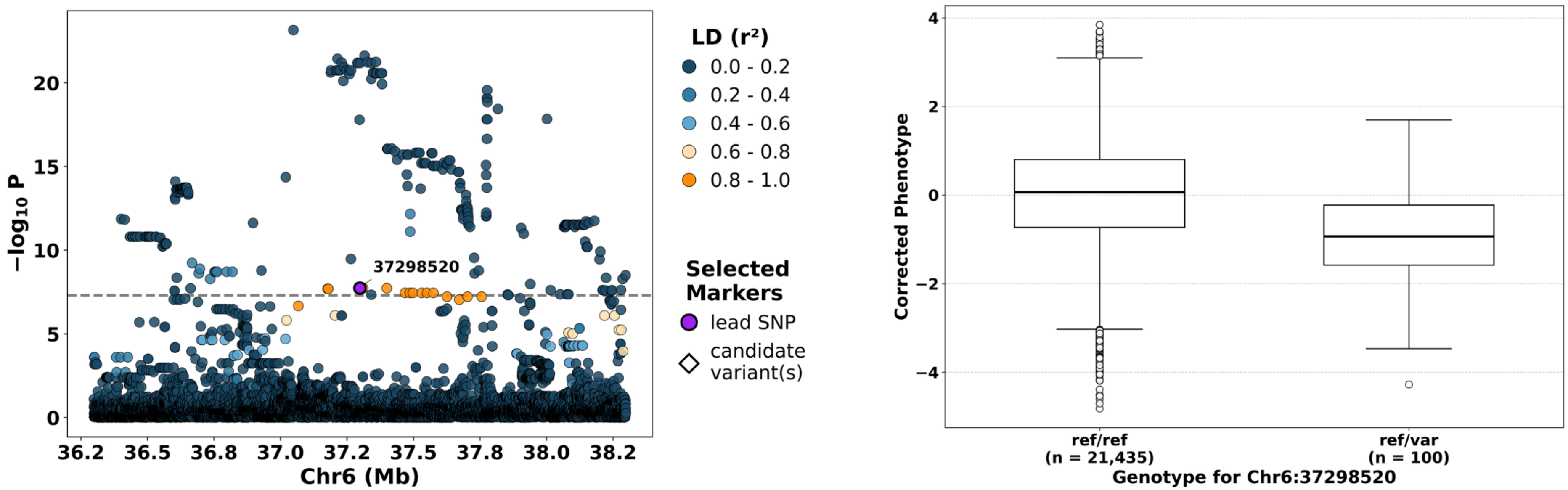

Additional file 4, Figure S8. Regional association plot and genotype-wise phenotype distribution for selected markers in BST (HO), chromosome 7, under the additive model.

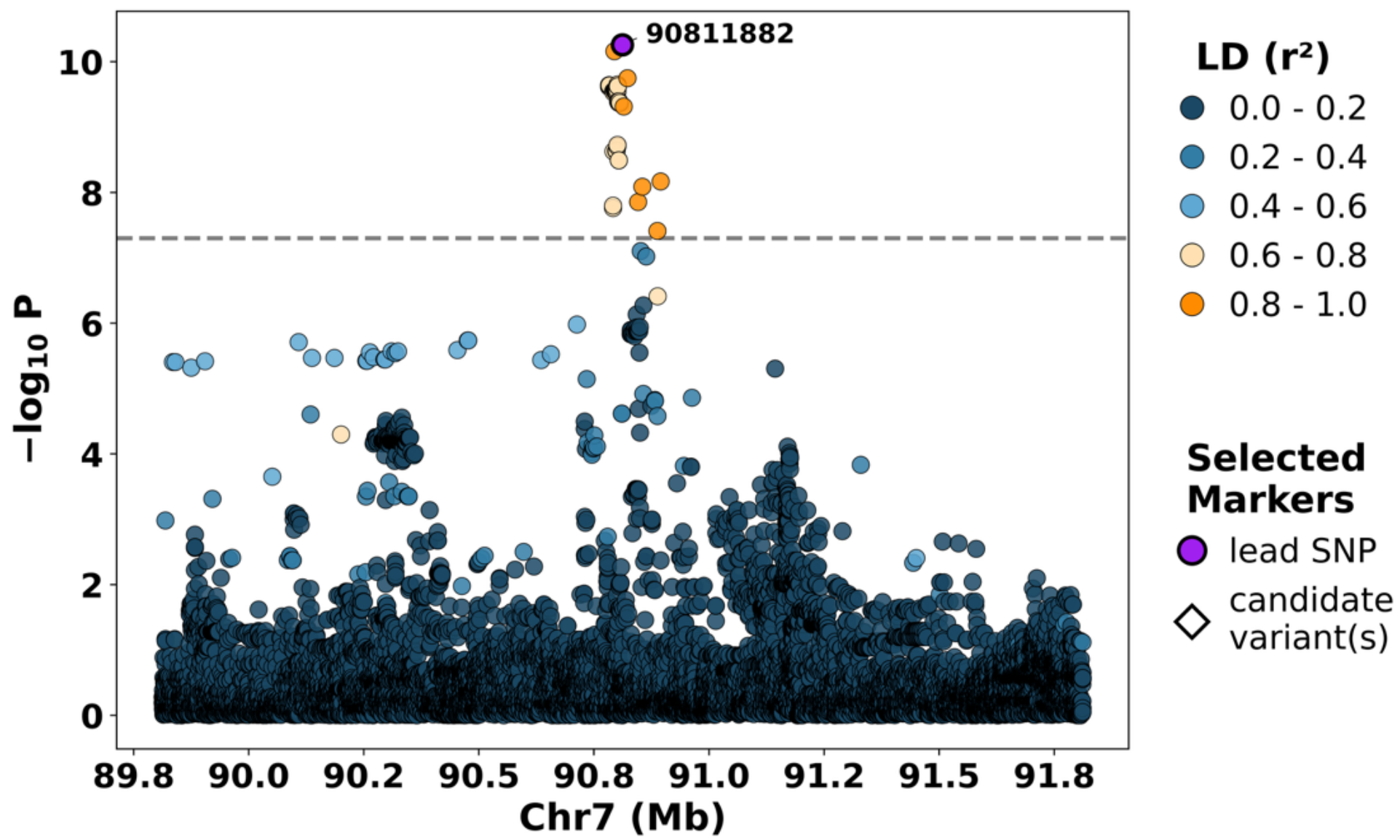

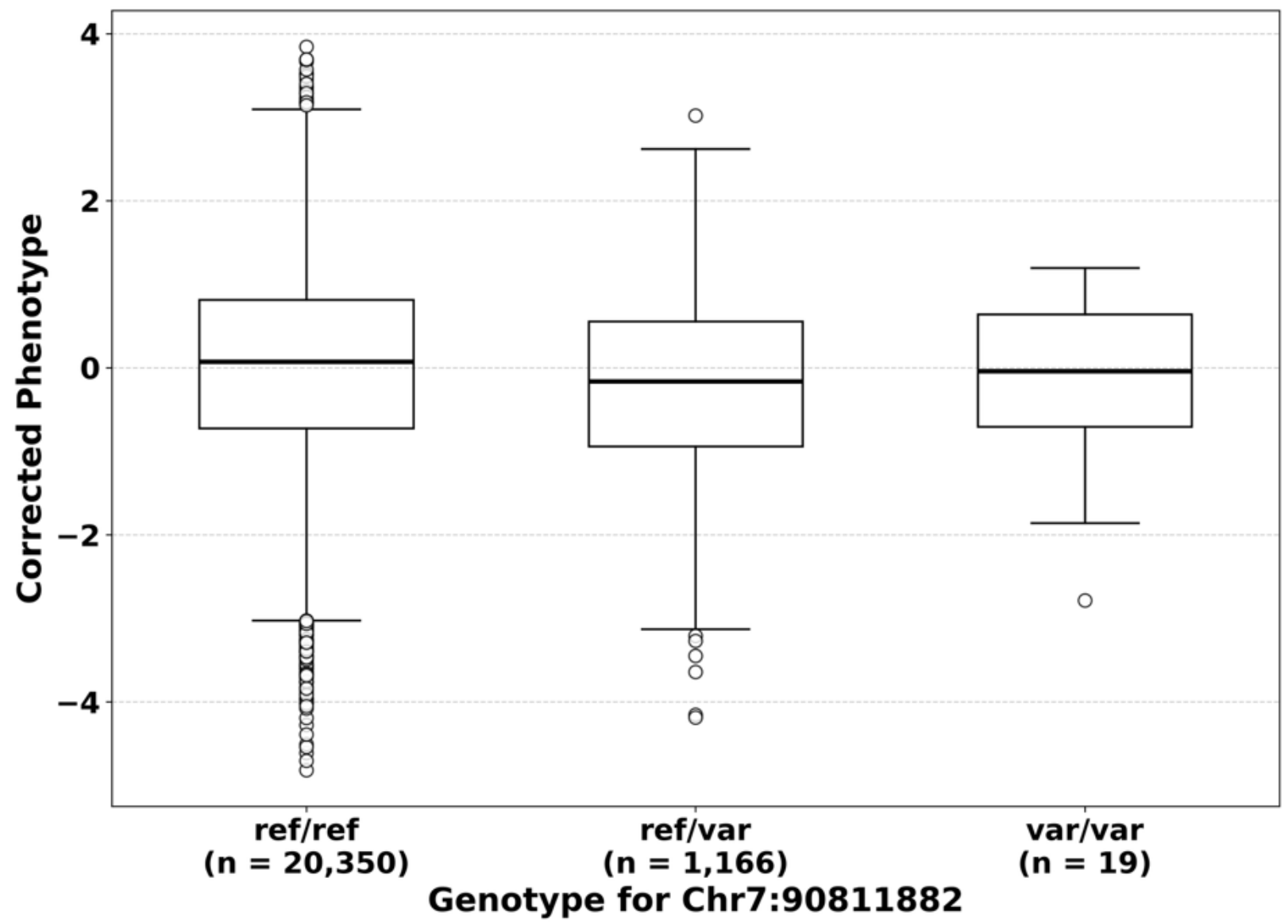

Additional file 4, Figure S9. Regional association plot and genotype-wise phenotype distribution for selected markers in BST (HO), chromosome 14, under the additive model.

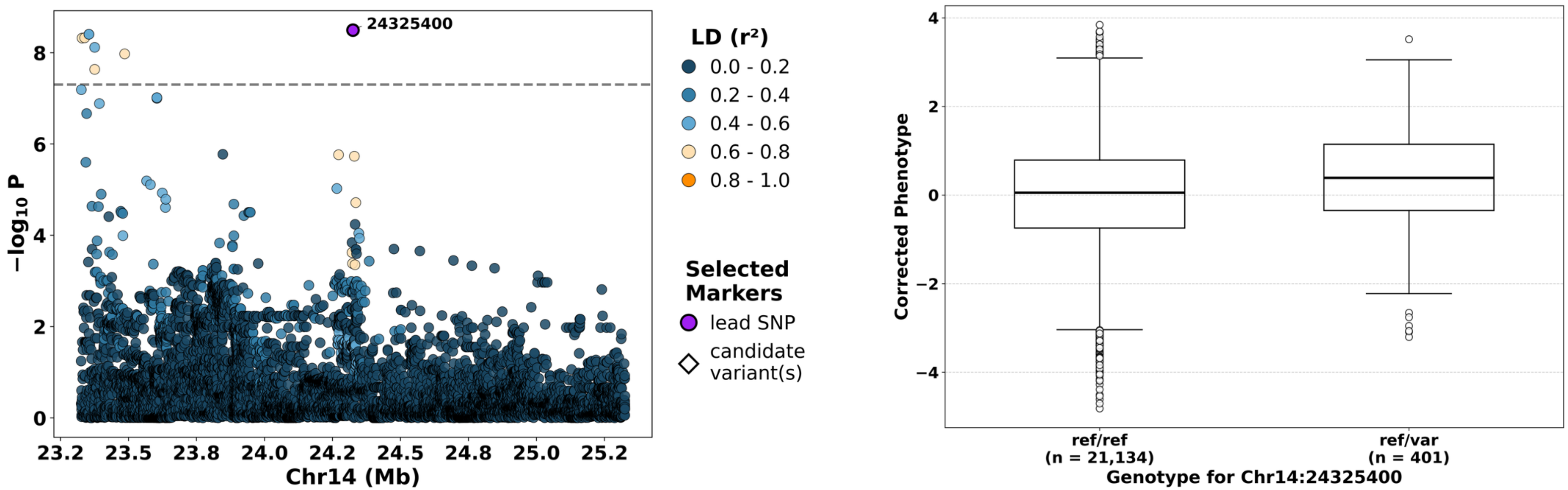

Additional file 4, Figure S10. Regional association plot and genotype-wise phenotype distribution for selected markers in BST (HO), chromosome 18, under the additive model.

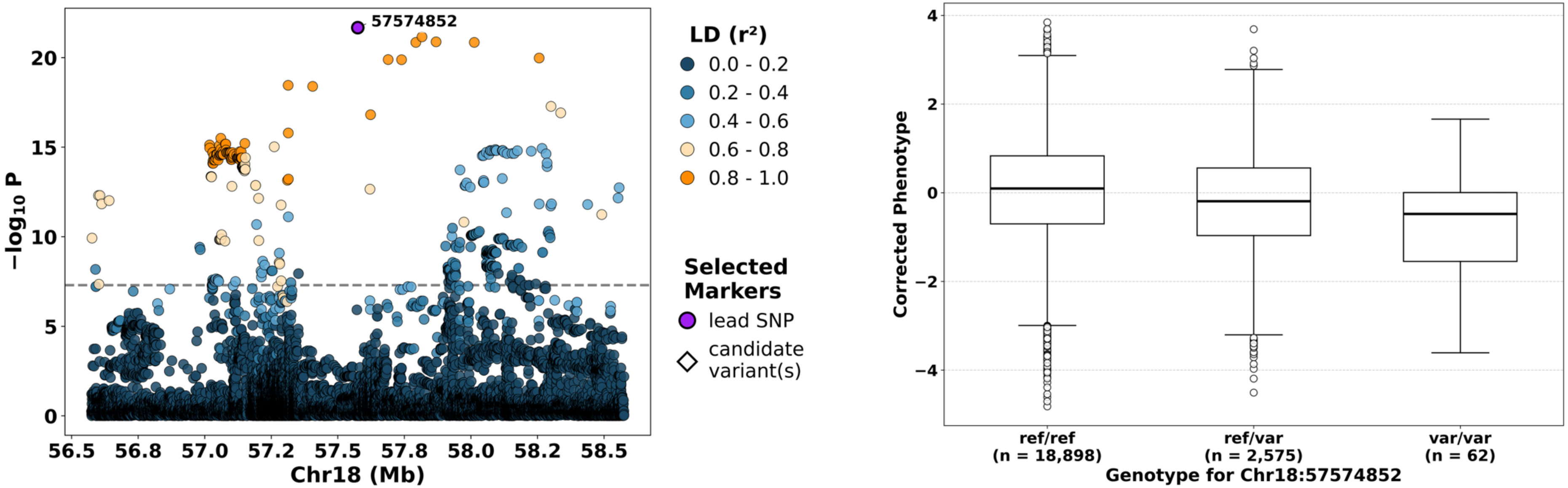

Additiona file 4, Figure S11. Regional association plot and genotype-wise phenotype distribution for selected markers in BST (HO), chromosome 5, under the dominant model.

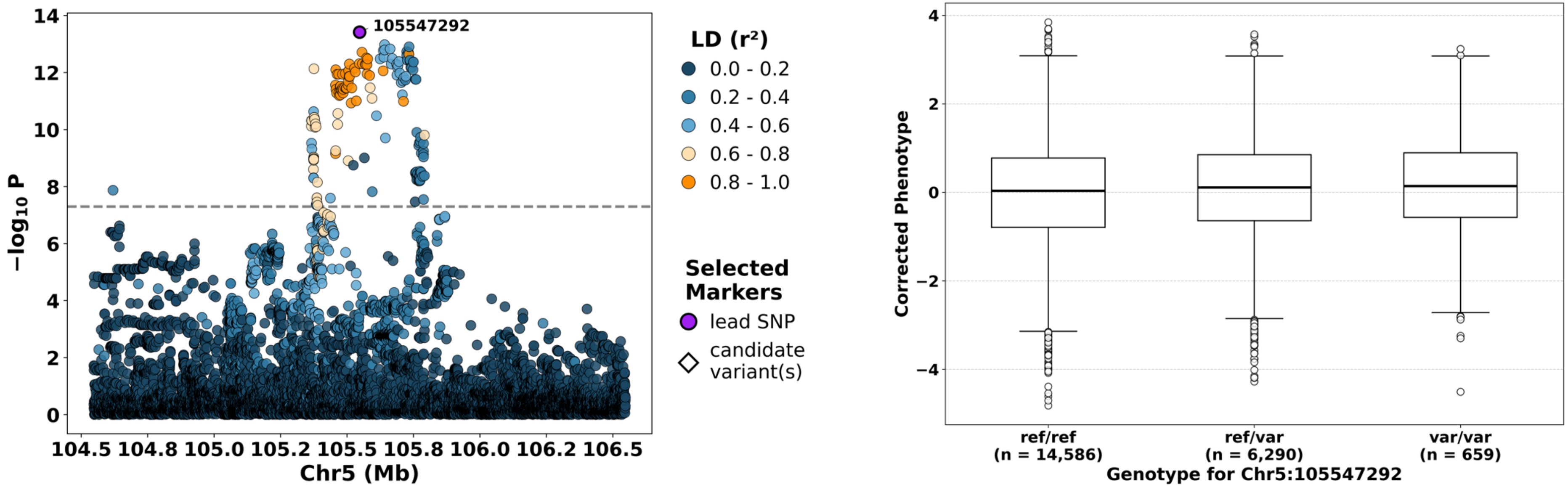

Additional file 4, Figure S12. Regional association plot and genotype-wise phenotype distribution for selected markers in BST (HO), chromosome 6 for the primary independent signal, under the dominant model.

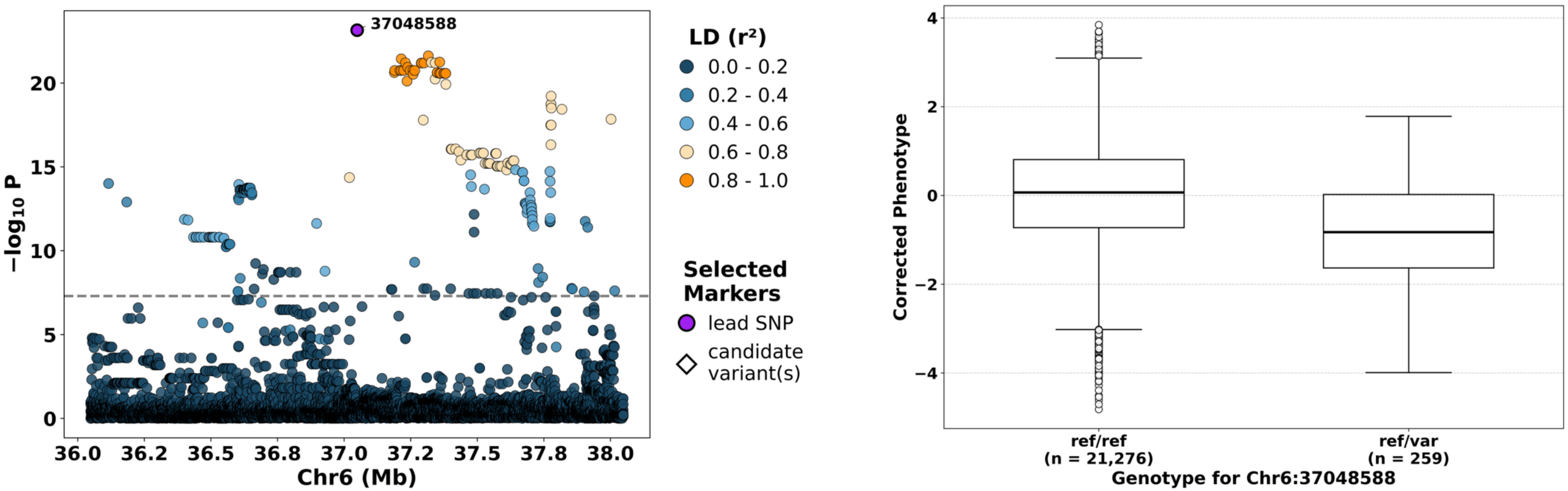

Additional file 4, Figure S13. Regional association plot and genotype-wise phenotype distribution for selected markers in BST (HO), chromosome 6 for the secondary independent signal, under the dominant model.

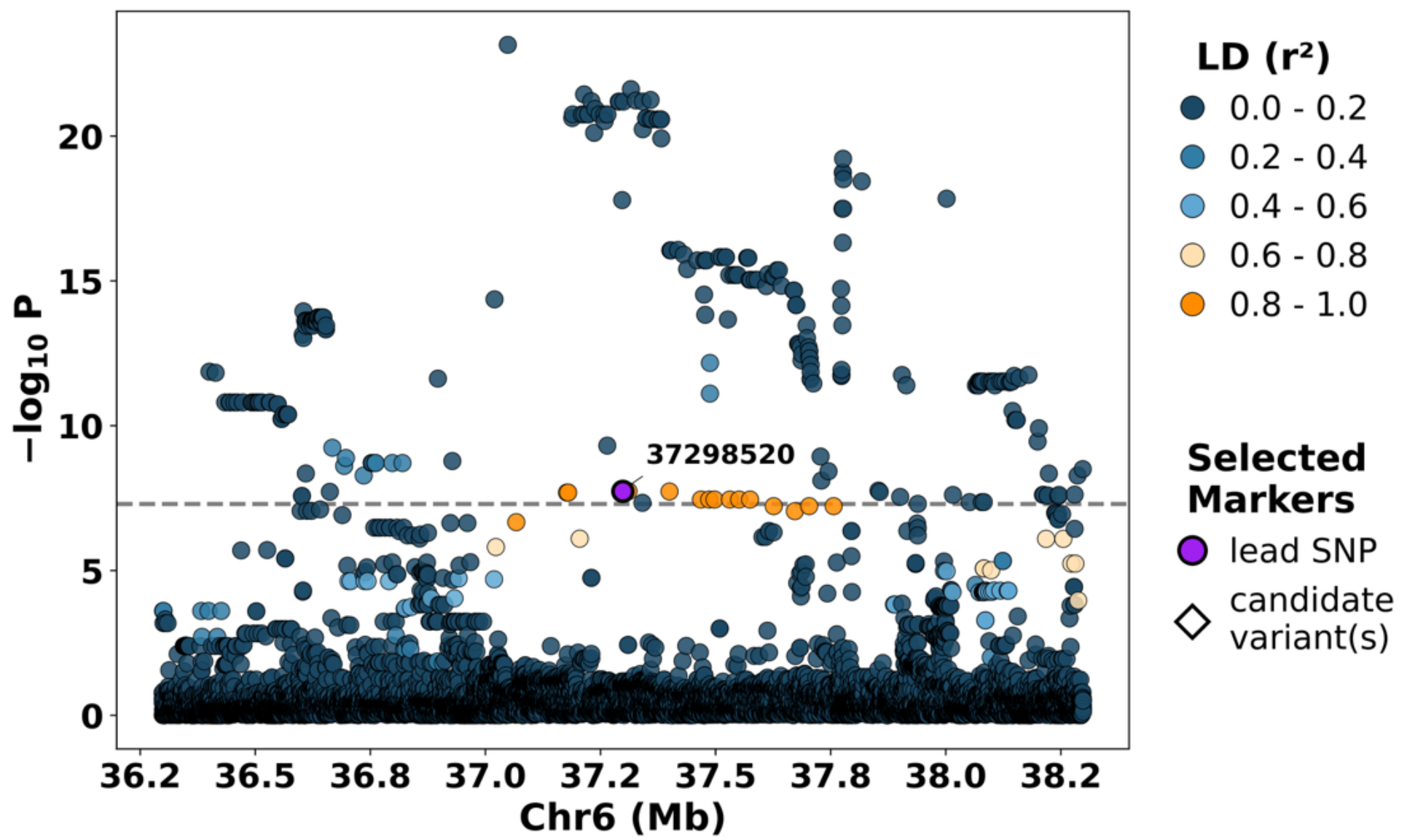

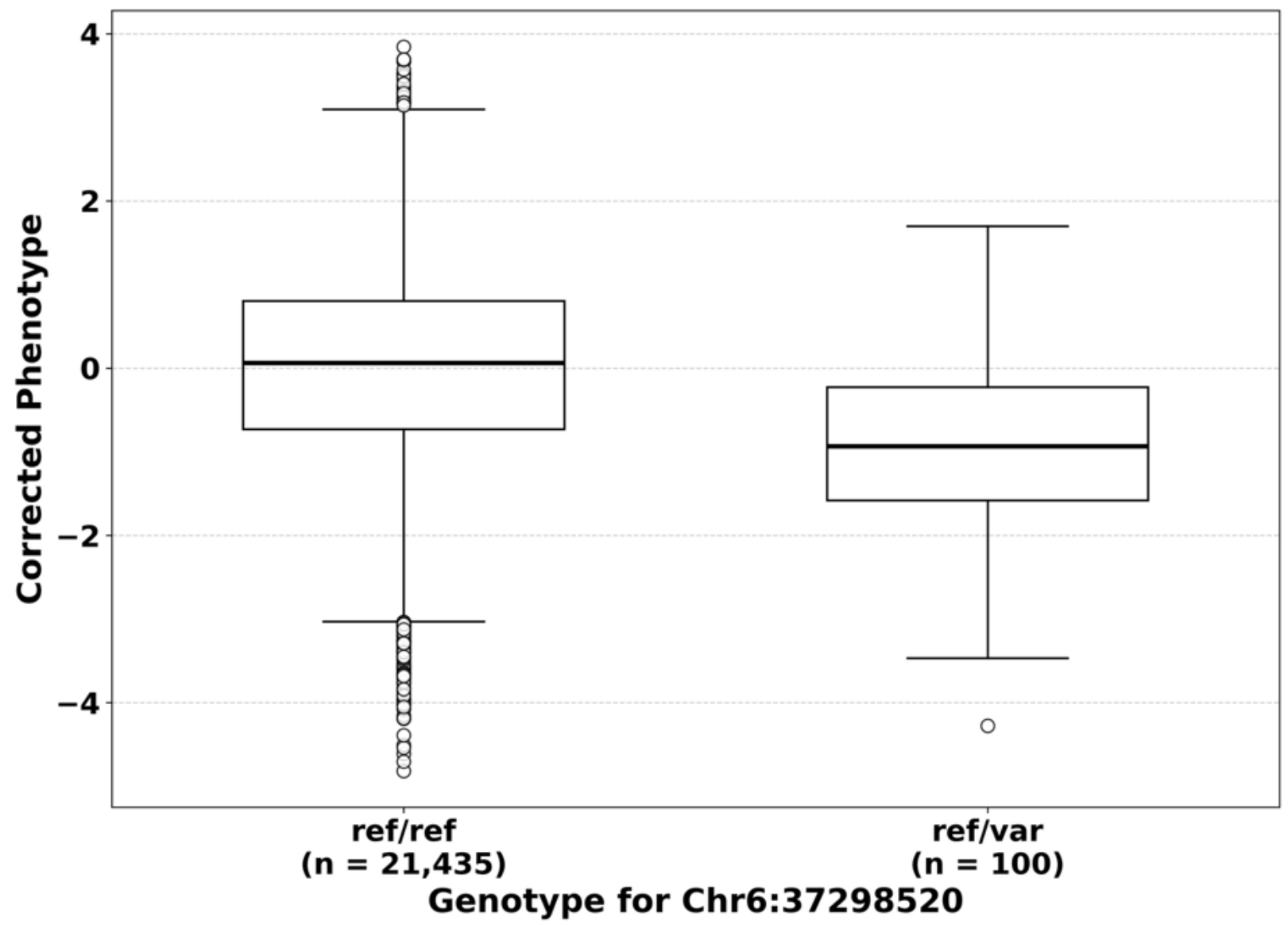

Additional file 4, Figure S14. Regional association plot and genotype-wise phenotype distribution for selected markers in BST (HO), chromosome 7, under the dominant model.

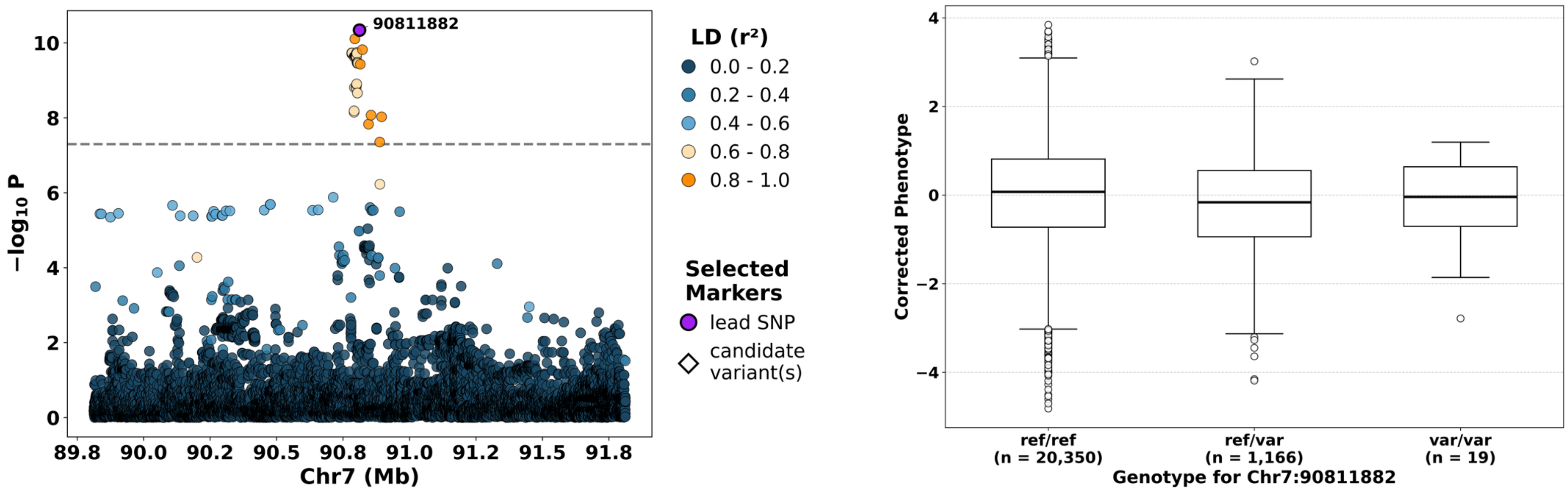

Additional file 4, Figure S15. Regional association plot and genotype-wise phenotype distribution for selected markers in BST (HO), chromosome 14, under the dominant model.

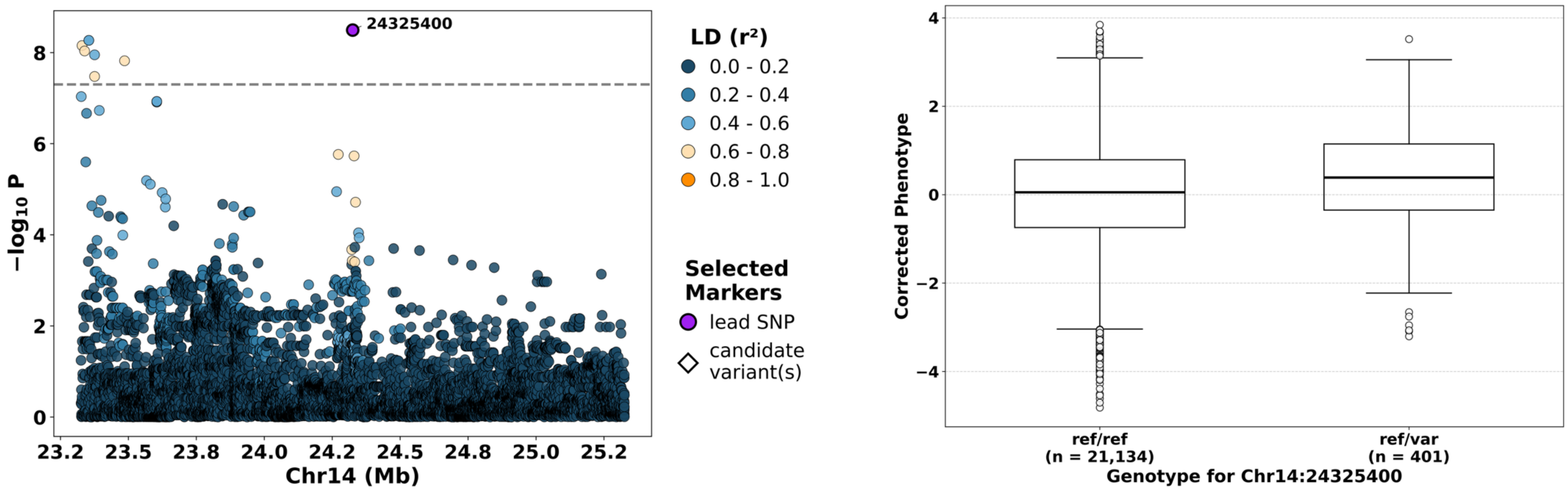

Additional file 4, Figure S16. Regional association plot and genotype-wise phenotype distribution for selected markers in BST (HO), chromosome 18, under the dominant model.

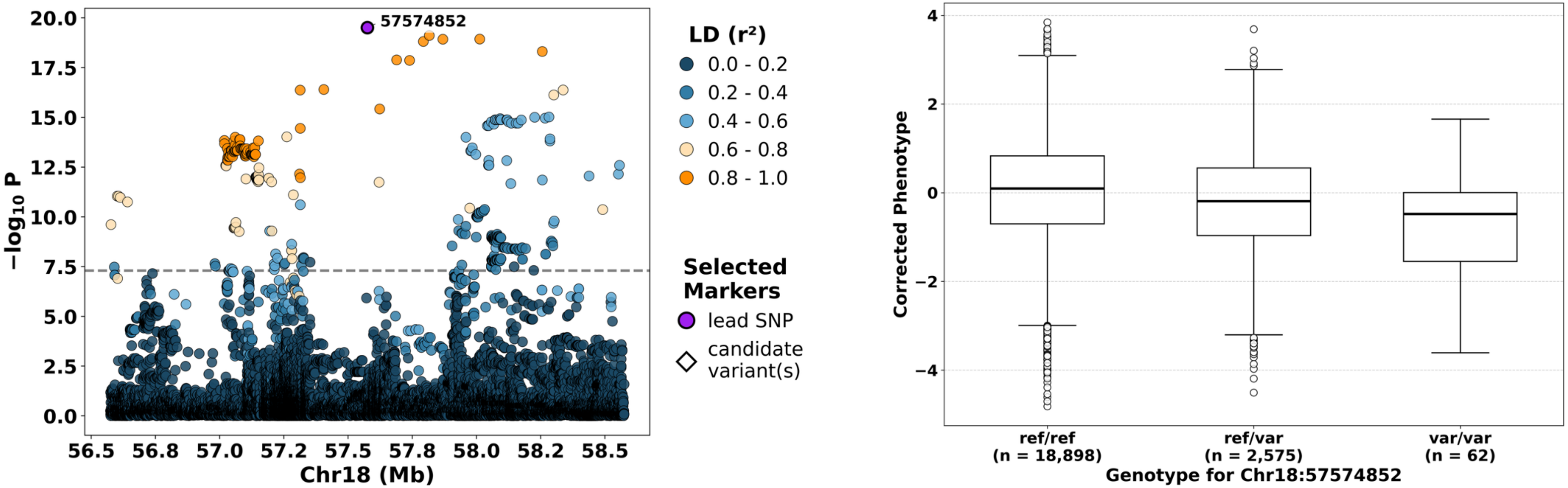

Additional file 4, Figure S17. Regional association plot and genotype-wise phenotype distribution for selected markers in BST (HO), chromosome 18, under the dominant model.

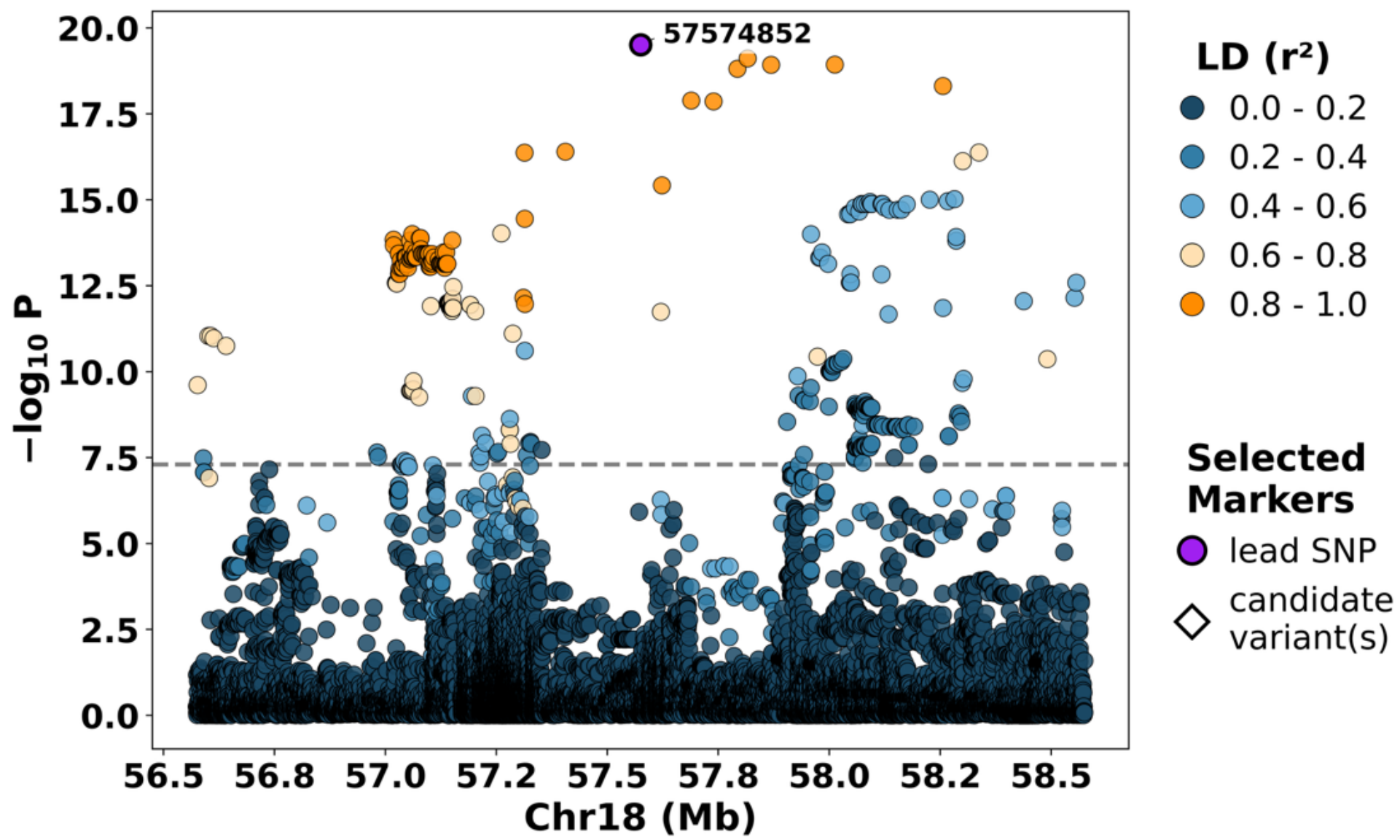

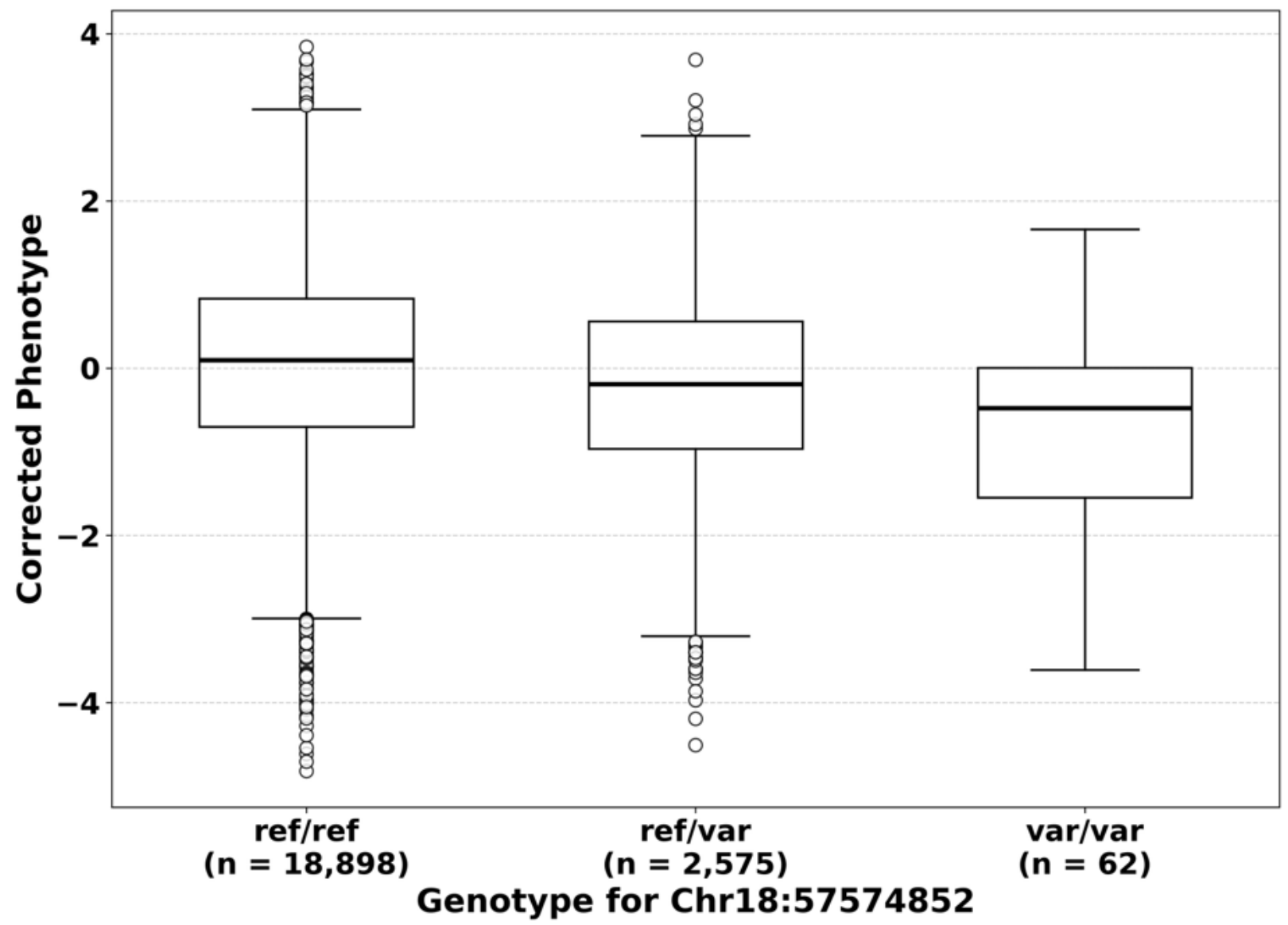

Additional file 4, Figure S18. Regional association plot and genotype-wise phenotype distribution for selected markers in DHE (BS), chromosome 13, under the additive model.

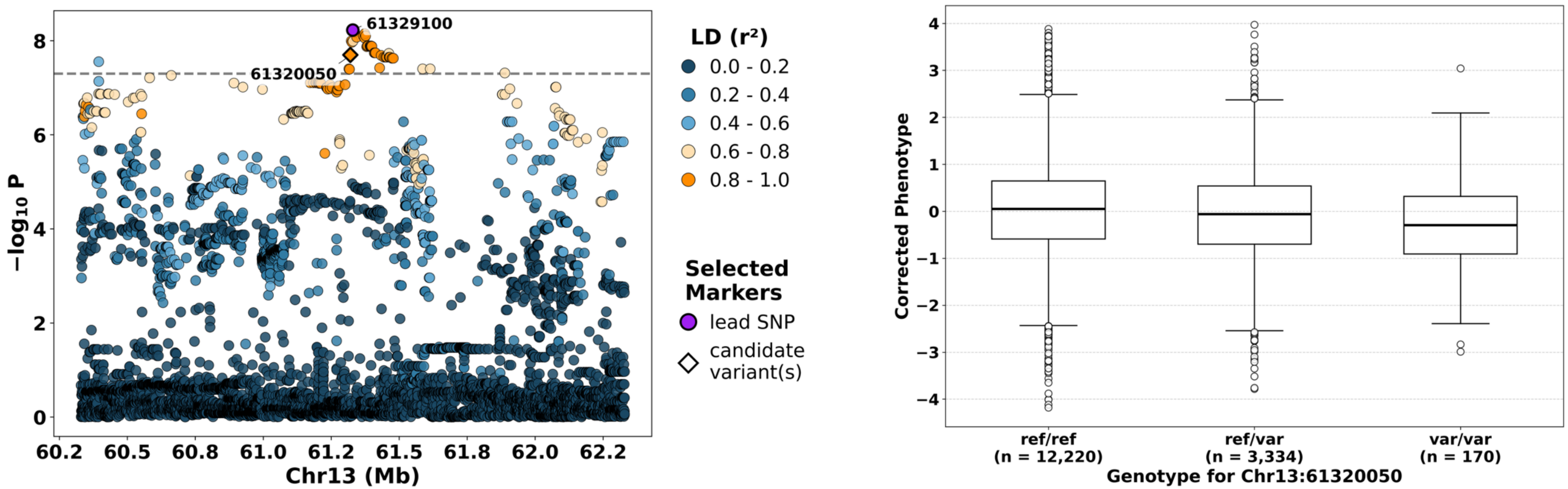

Additional file 4, Figure S19. Regional association plot and genotype-wise phenotype distribution for selected markers in DHE (BS), chromosome 25, under the additive model.

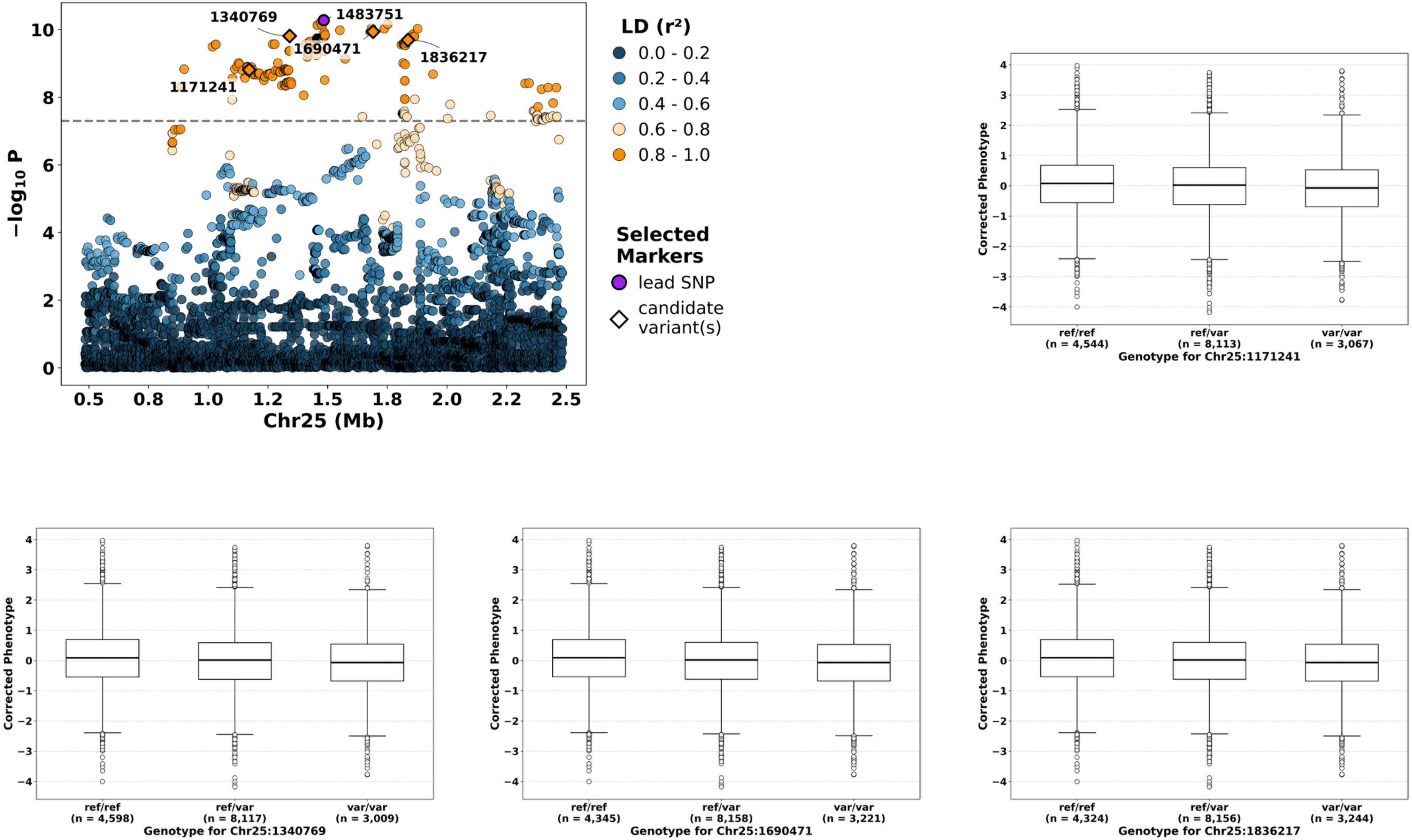

Additional file 4, Figure S20. Regional association plot and genotype-wise phenotype distribution for selected markers in DHE (BS), chromosome 13, under the dominant model.

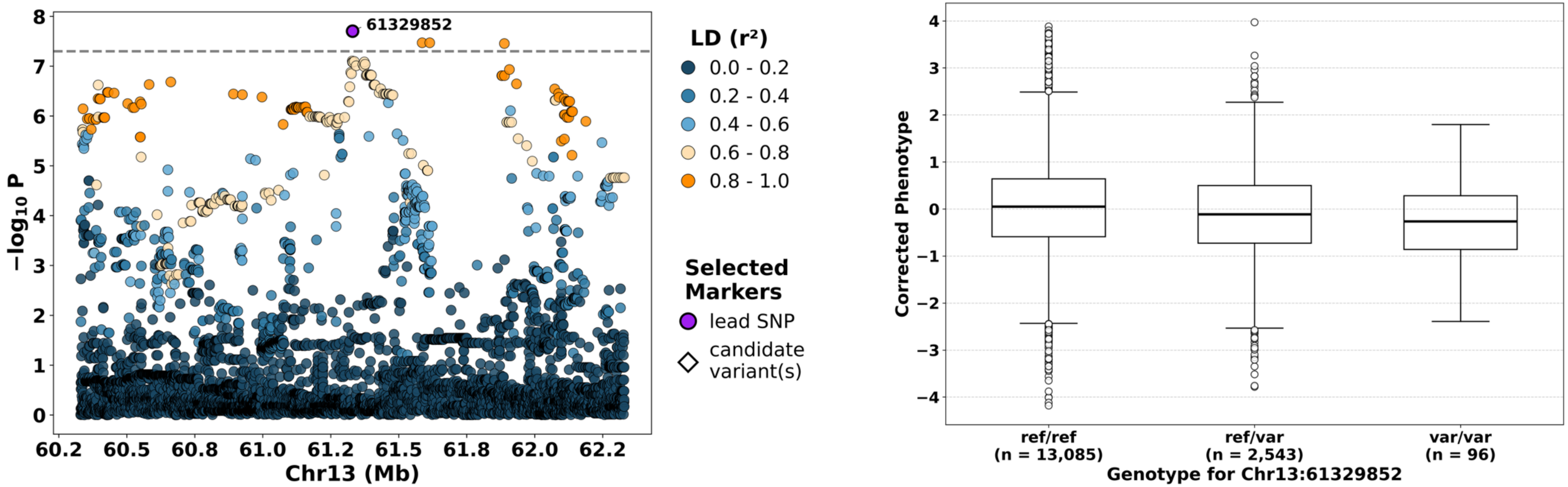

Additional file 4, Figure S21. Regional association plot and genotype-wise phenotype distribution for selected markers in DHE (BS), chromosome 25, under the dominant model.

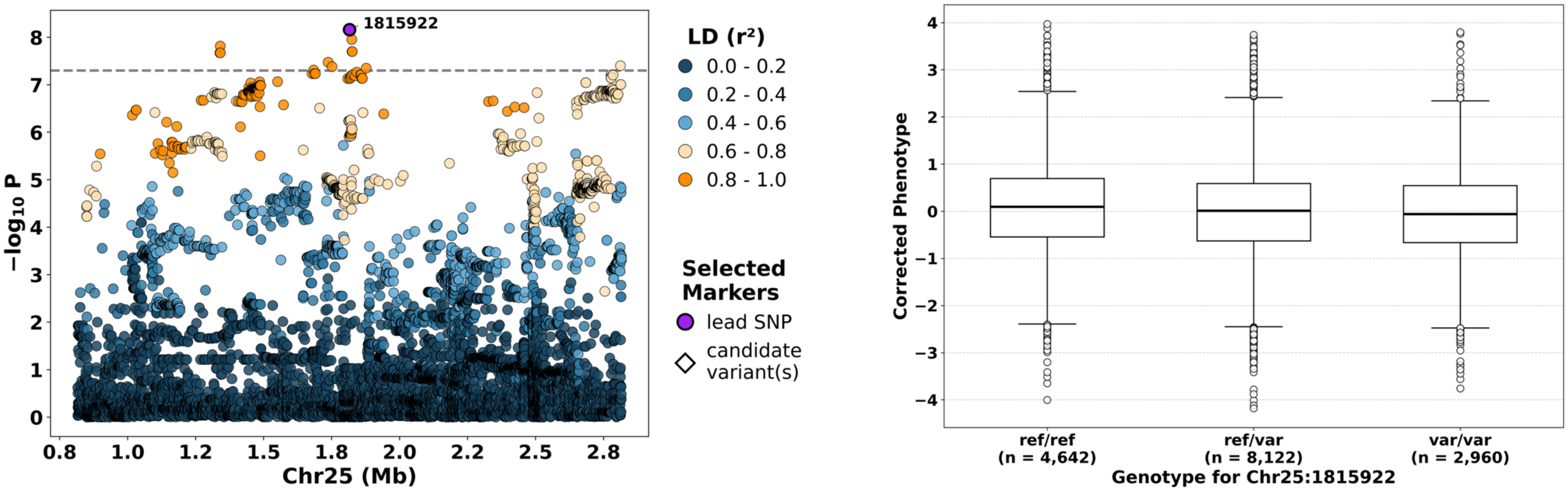

Additional file 4, Figure S22. Regional association plot and genotype-wise phenotype distribution for selected markers in HQU (BS), chromosome 3, under the additive model.

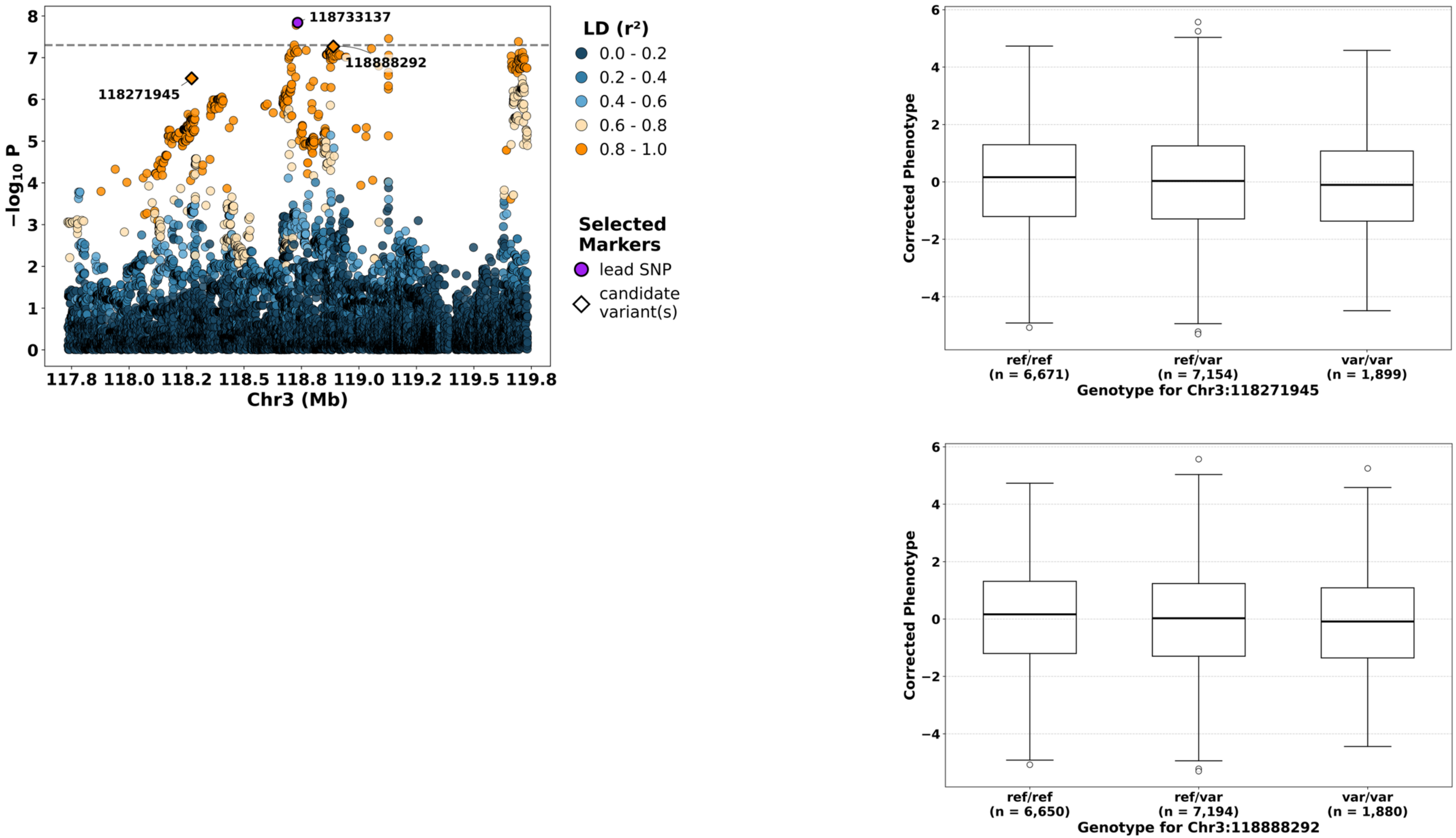

Additional file 4, Figure S23. Regional association plot and genotype-wise phenotype distribution for selected markers in HQU (BS), chromosome 18, under the additive model.

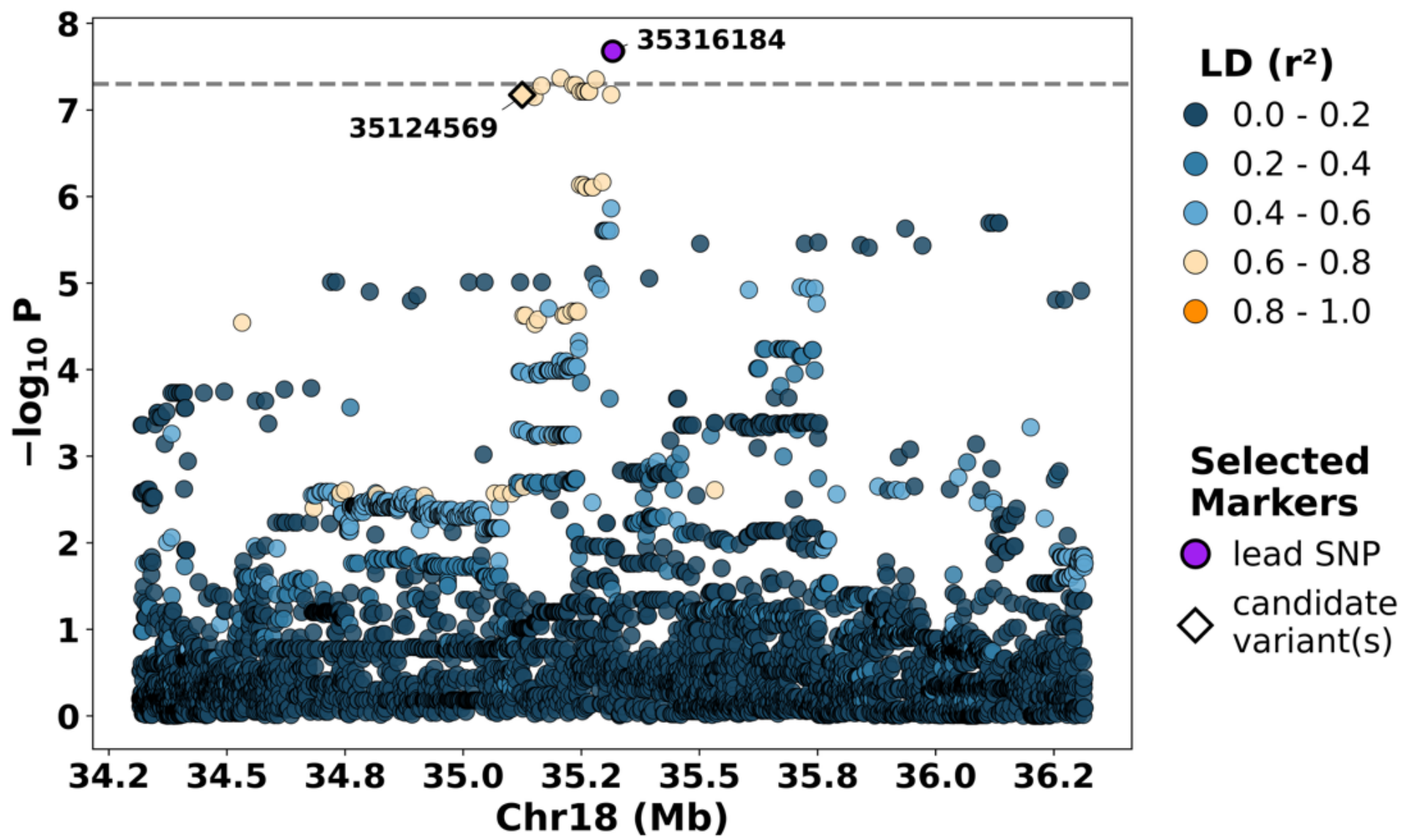

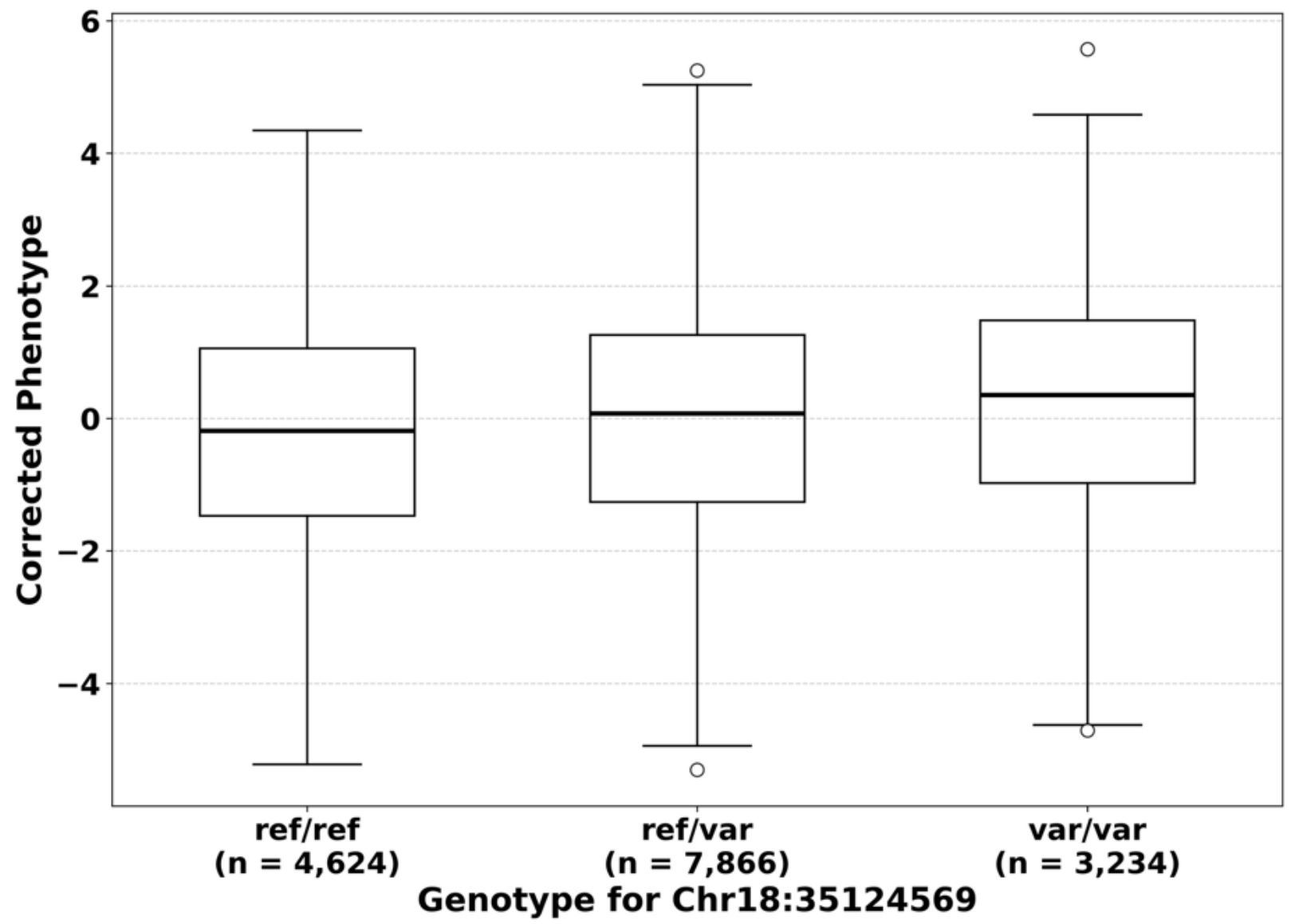

Additional file 4, Figure S24. Regional association plot and genotype-wise phenotype distribution for selected markers in HQU (BS), chromosome 25, under the additive model.

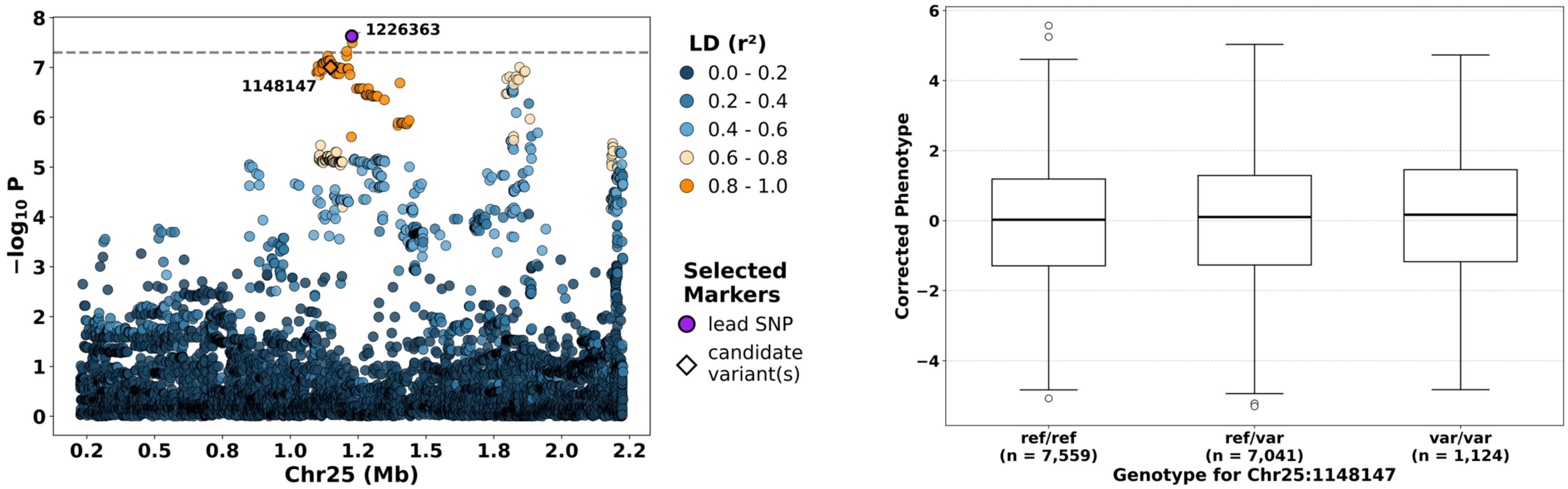

Additional file 4, Figure S25. Regional association plot and genotype-wise phenotype distribution for selected markers in HQU (BS), chromosome 26, under the additive model.

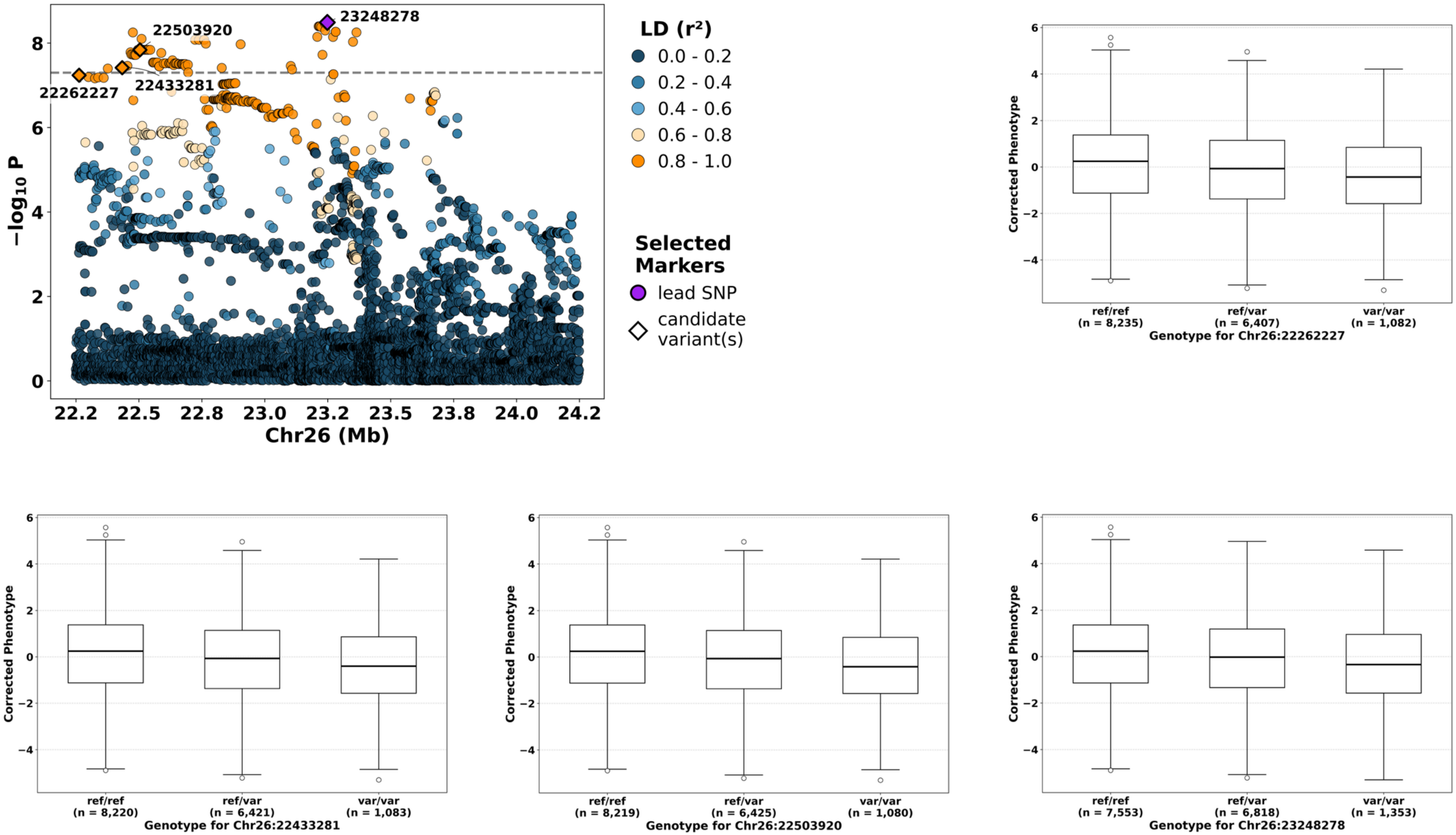

Additional file 4, Figure S26. Regional association plot and genotype-wise phenotype distribution for selected markers in HQU (BS), chromosome 6, under the dominant model.

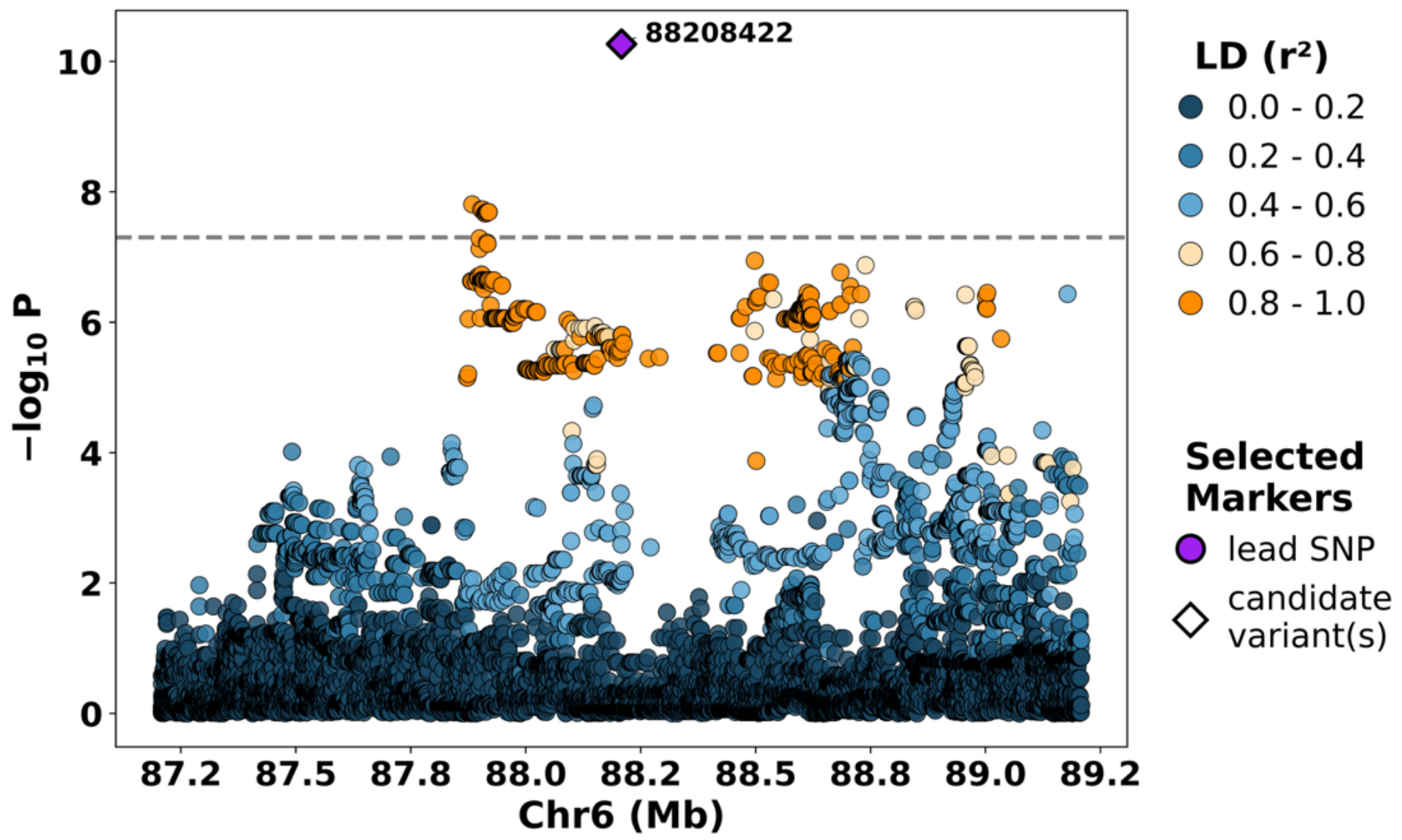

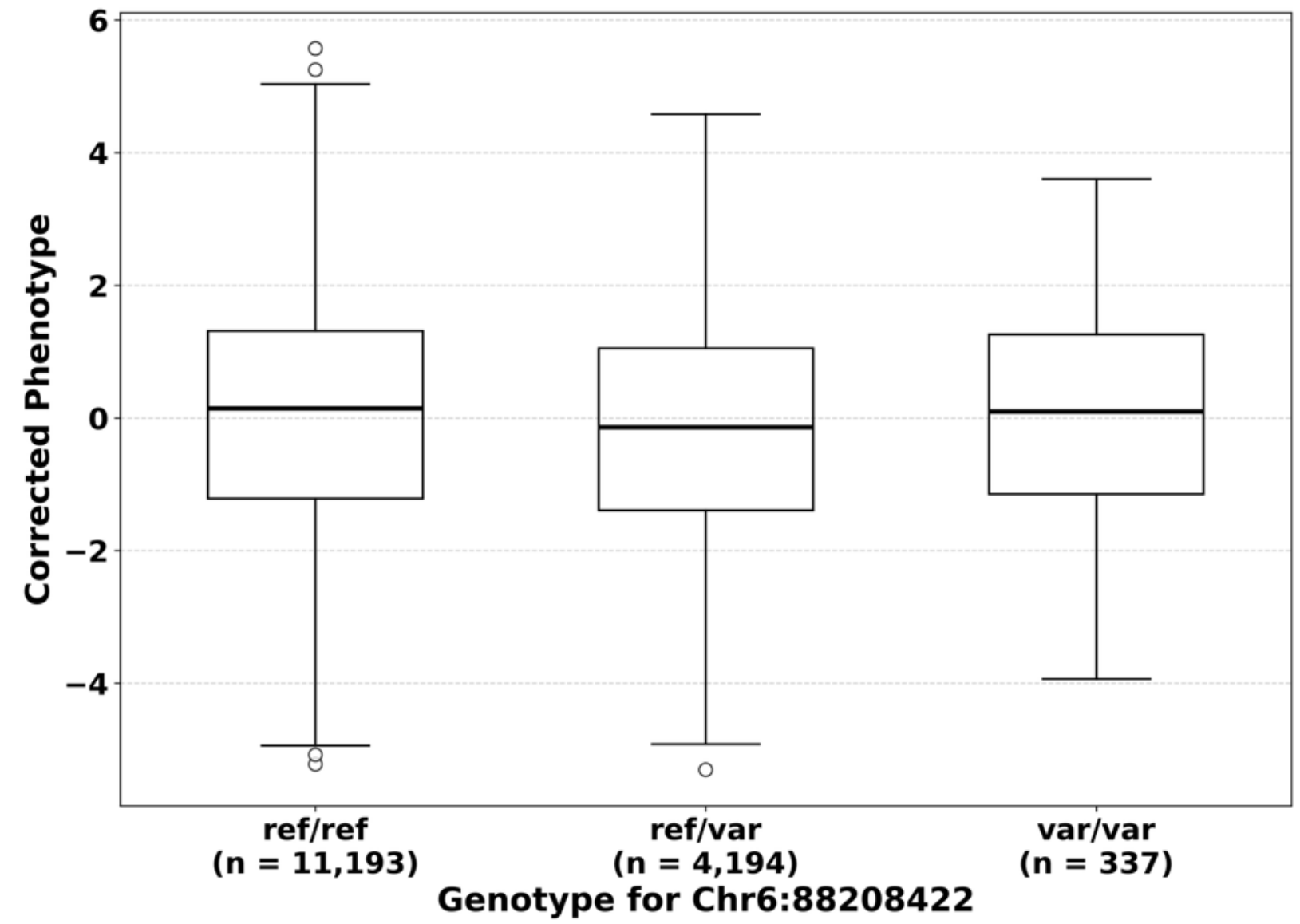

Additional file 4, Figure S27. Regional association plot and genotype-wise phenotype distribution for selected markers in HQU (BS), chromosome 18, under the dominant model.

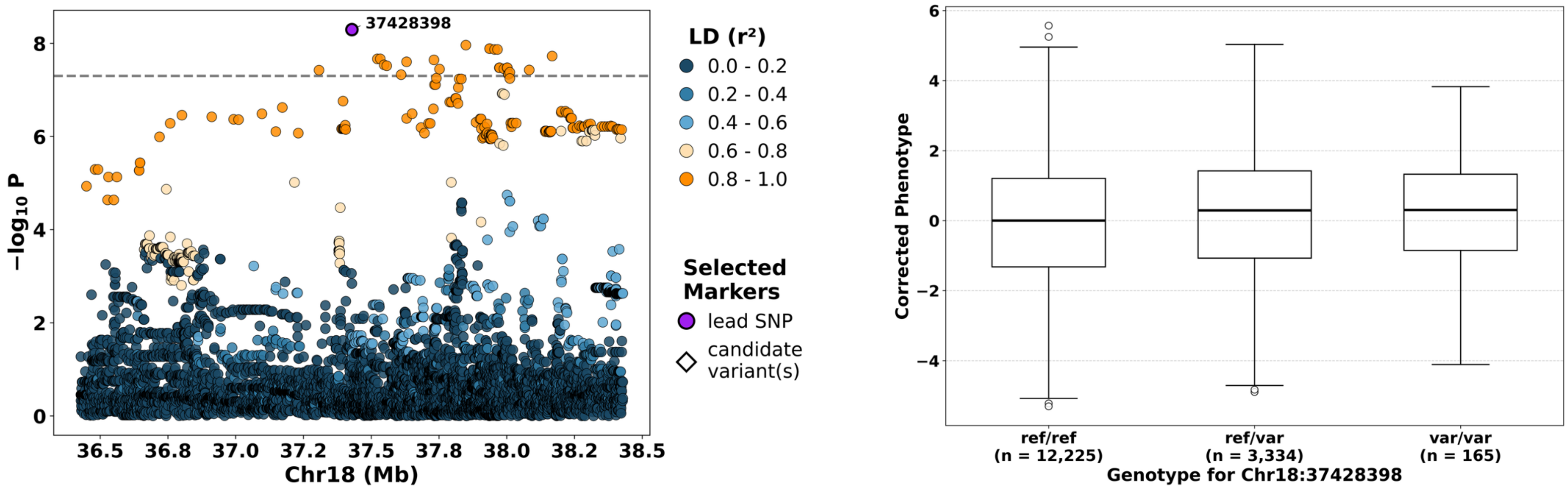

Supplement: Supplementary file 1 [file 12711_2026_1068_MOESM4_ESM.pdf]
