# Supplementary material for: Genomic dissection of hoof and leg conformation in Swiss dairy cattle populations reveals polygenic architecture and a recessive HYAL1 nonsense variant affecting longevity in Holstein cattle
Source: Genet Sel Evol. 2026 Jul 5;58:46. doi: 10.1186/s12711-026-01068-3 (PMC13491847; doi:10.1186/s12711-026-01068-3)

Additional file 8, Figure S28. Quantile–quantile plots for BST in HO under additive (a), dominant (b), and recessive (c) models, with the genomic inflation factor (λ) reported in each panel.

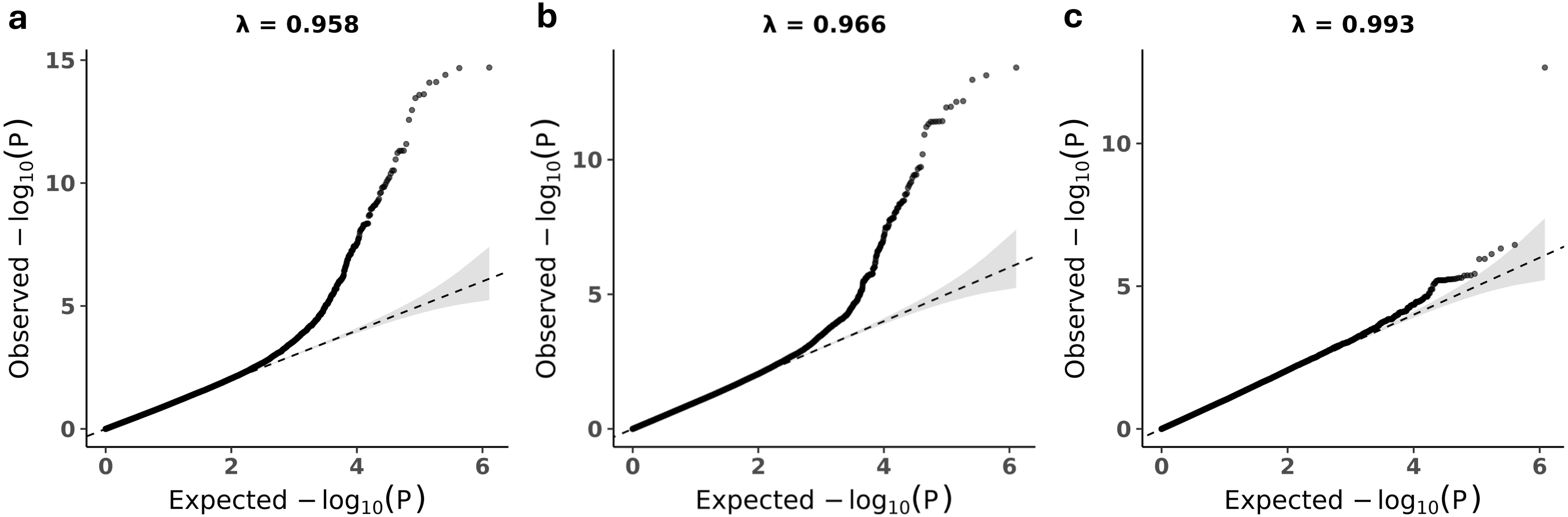

Additional file 8, Figure S29. Quantile–quantile plots for HDE in HO under additive (a), dominant (b), and recessive (c) models, with the genomic inflation factor (λ) reported in each panel.

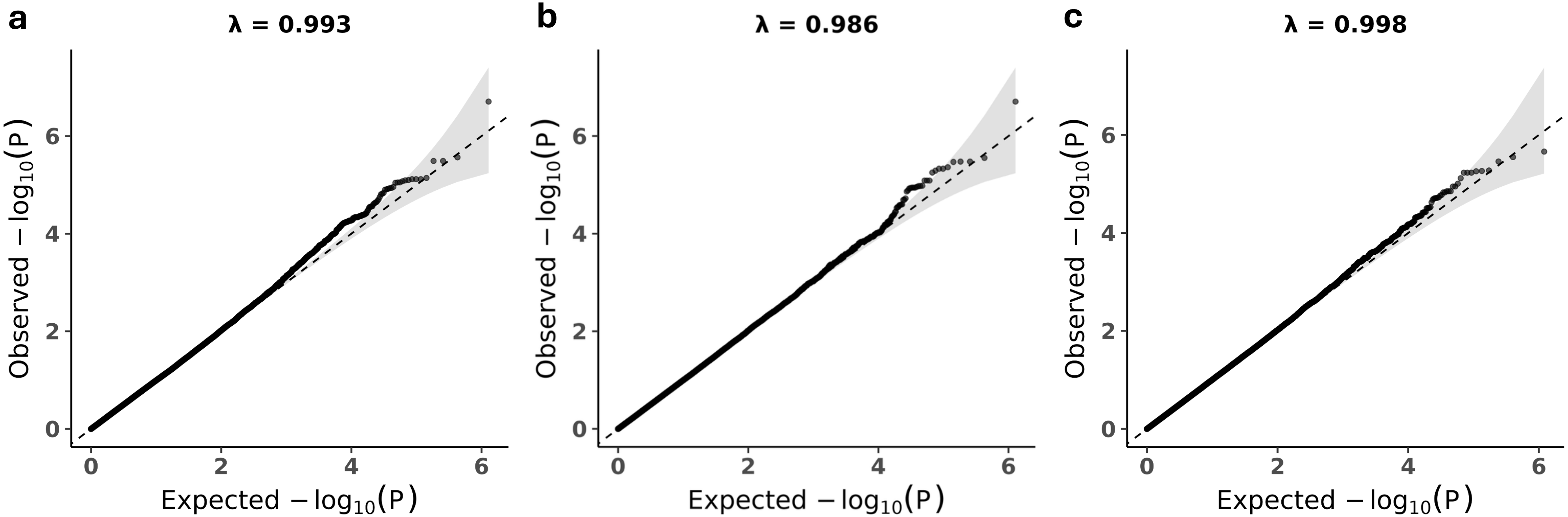

Additional file 8, Figure S30. Quantile–quantile plots for FAN in HO under additive (a), dominant (b), and recessive (c) models, with the genomic inflation factor (λ) reported in each panel.

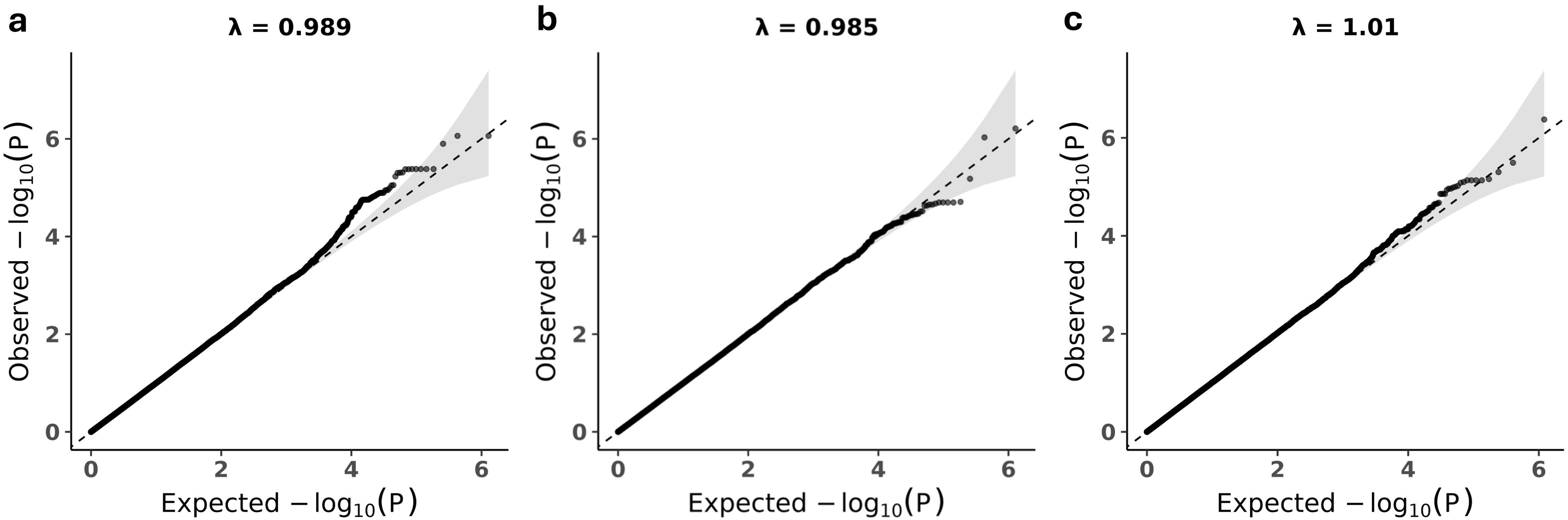

Additional file 8, Figure S31. Quantile–quantile plots for LOC in HO under additive (a), dominant (b), and recessive (c) models, with the genomic inflation factor (λ) reported in each panel.

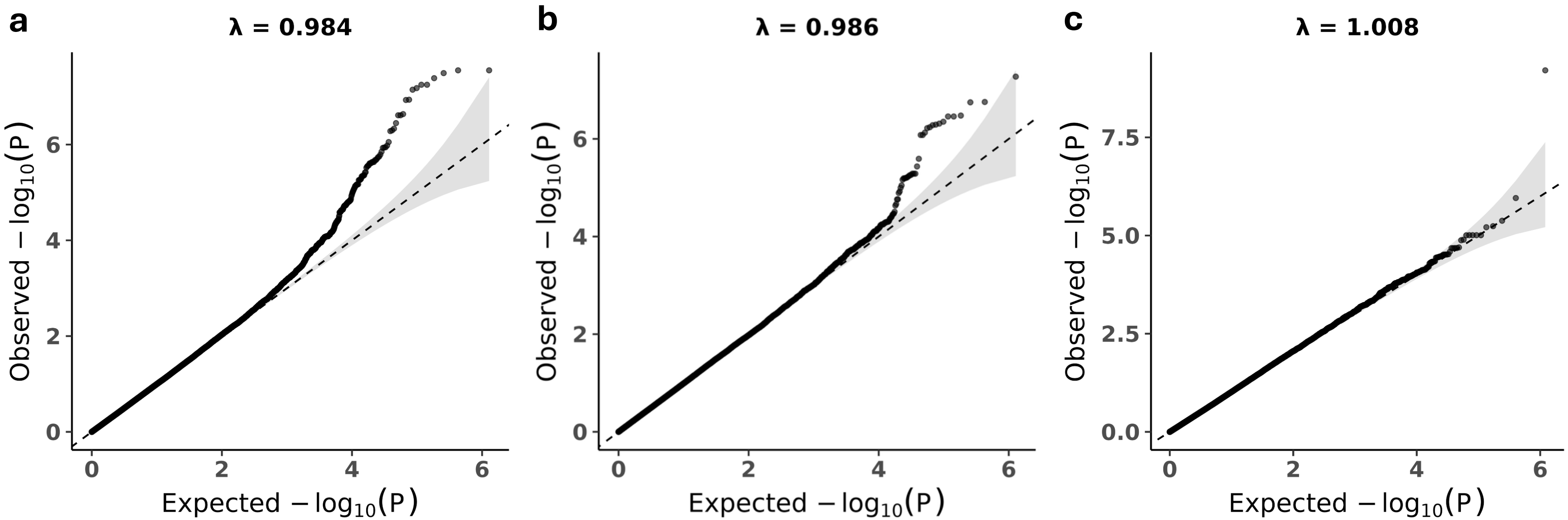

Additional file 8, Figure S32. Quantile–quantile plots for RLR in HO under additive (a), dominant (b), and recessive (c) models, with the genomic inflation factor (λ) reported in each panel.

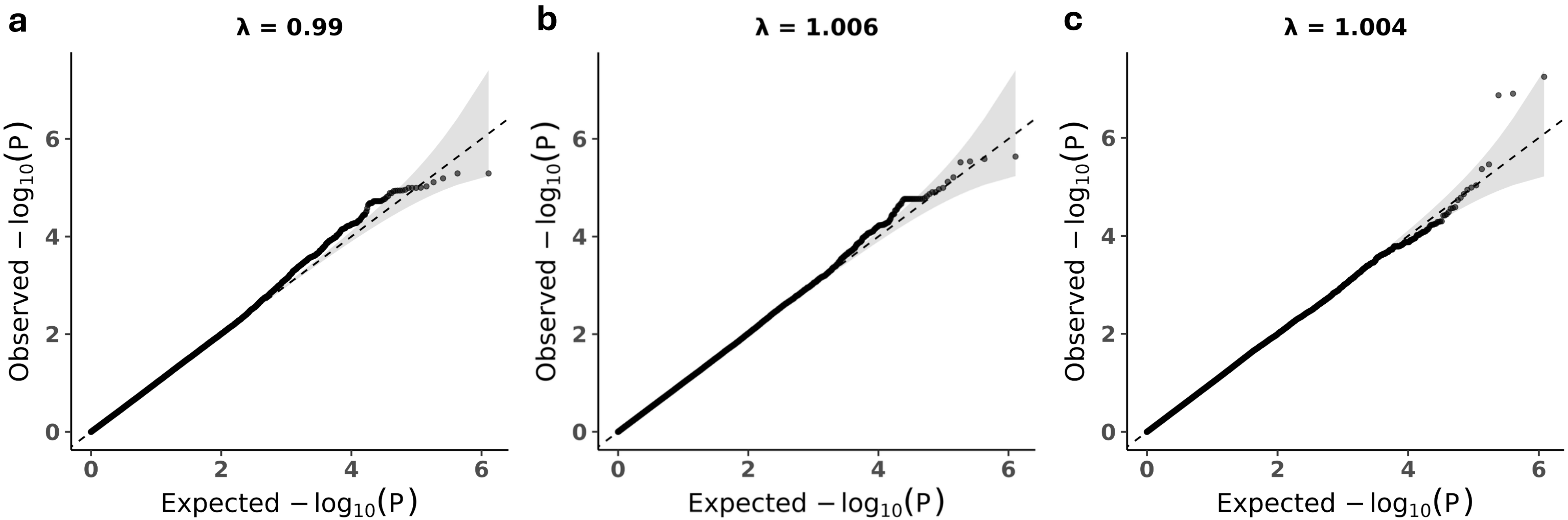

Additional file 8, Figure S33. Quantile–quantile plots for RLS in HO under additive (a), dominant (b), and recessive (c) models, with the genomic inflation factor (λ) reported in each panel.

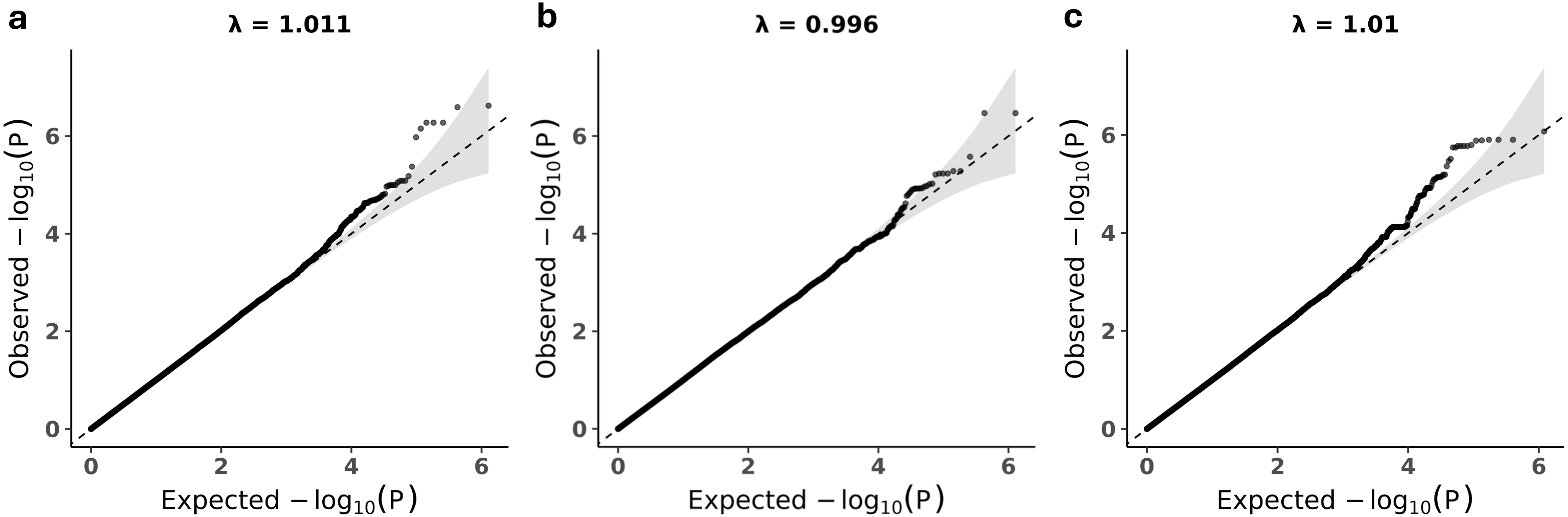

Additional file 8, Figure S34. Quantile–quantile plots for HDE in BS under additive (a), dominant (b), and recessive (c) models, with the genomic inflation factor (λ) reported in each panel.

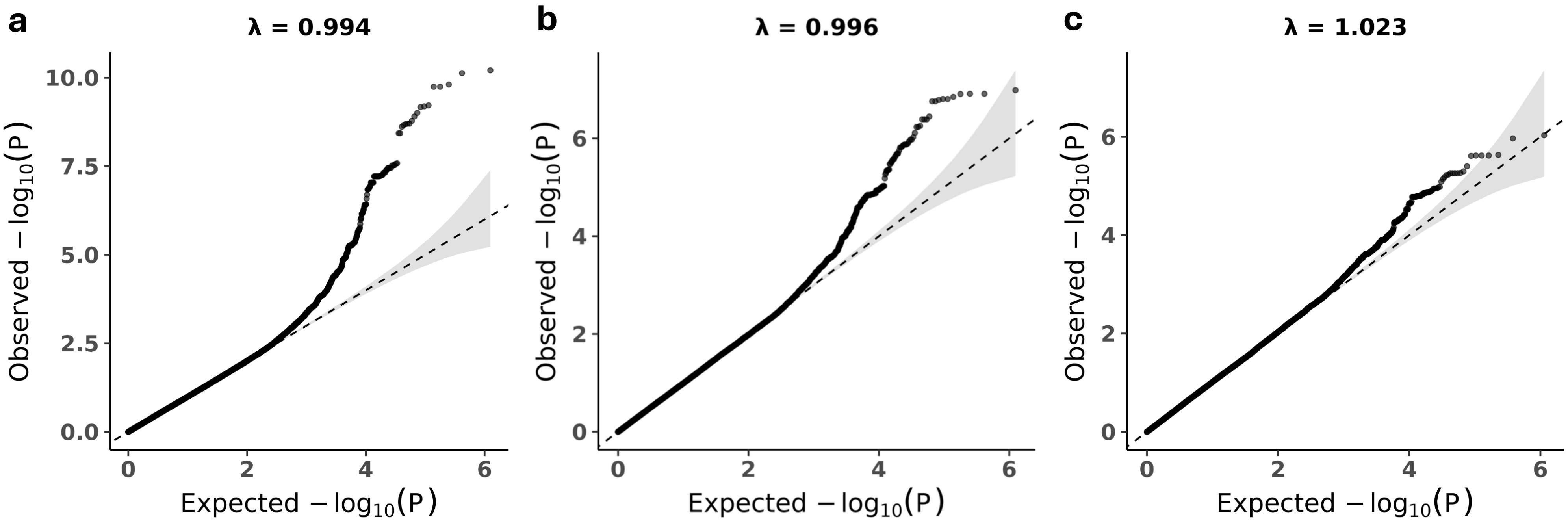

Additional file 8, Figure S35. Quantile–quantile plots for FAN in BS under additive (a), dominant (b), and recessive (c) models, with the genomic inflation factor (λ) reported in each panel.

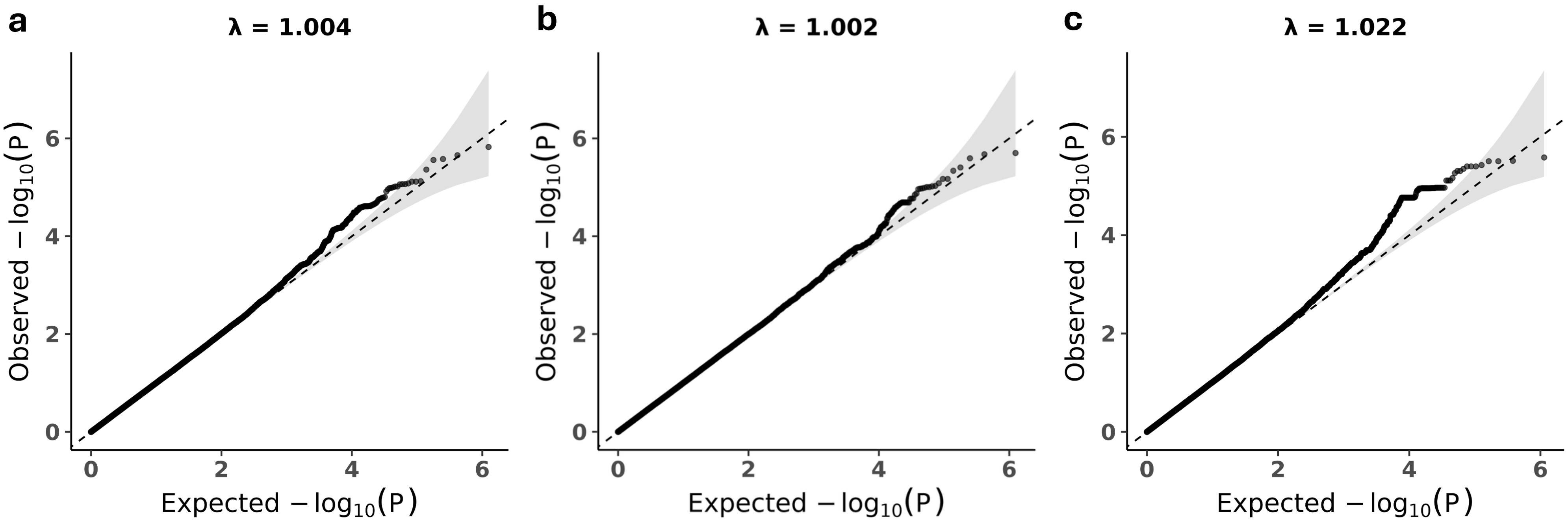

Additional file 8, Figure S36. Quantile–quantile plots for HQU in BS under additive (a), dominant (b), and recessive (c) models, with the genomic inflation factor (λ) reported in each panel.

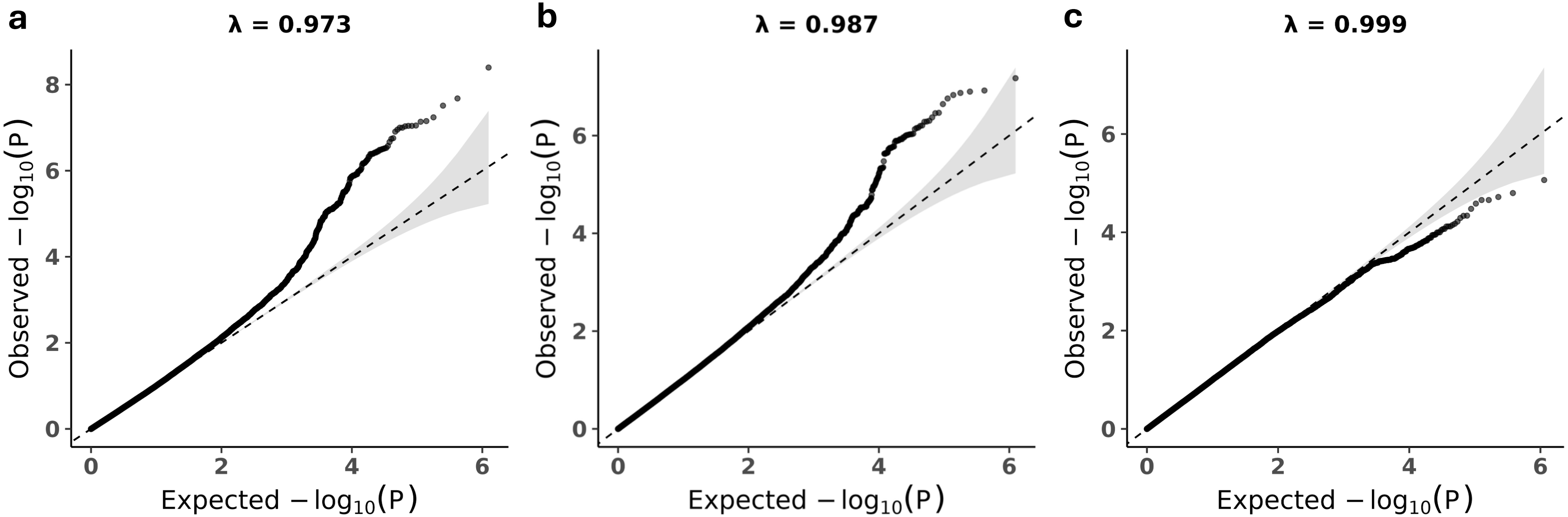

Additional file 8, Figure S37. Quantile–quantile plots for RLV in BS under additive (a), dominant (b), and recessive (c) models, with the genomic inflation factor (λ) reported in each panel.

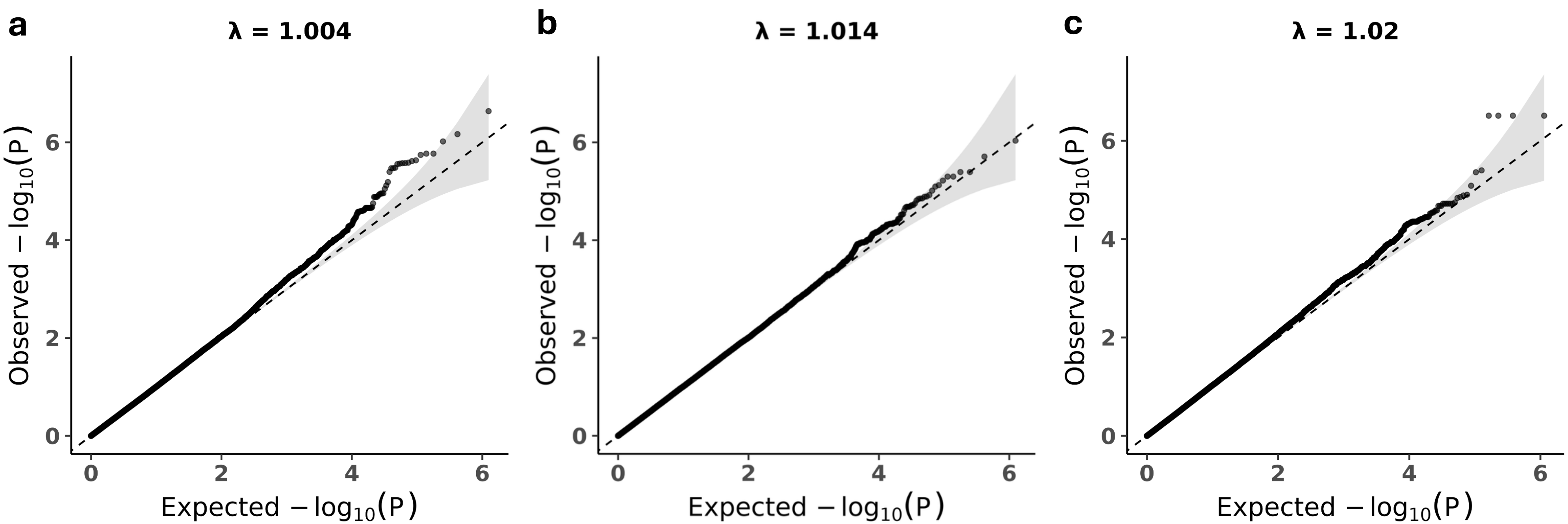

Supplement: Supplementary file 2 [file 12711_2026_1068_MOESM8_ESM.pdf]
